# Transcriptional differentiation of UV‐B protectant genes in maize landraces spanning an elevational gradient in Chiapas, Mexico

**DOI:** 10.1111/eva.12954

**Published:** 2020-04-03

**Authors:** Matthew A. Kost, Hugo Perales, Saranga Wijeratne, Asela J. Wijeratne, Eric J. Stockinger, Kristin L. Mercer

**Affiliations:** ^1^ Department of Horticulture and Crop Science The Ohio State University Wooster OH USA; ^2^ Departamento de Agricultura, Sociedad y Ambiente El Colegio de la Frontera Sur San Cristóbal de Las Casas Chiapas Mexico; ^3^ Molecular and Cellular Imaging Center Ohio Agricultural Research and Development Center The Ohio State University Wooster OH USA; ^4^ Department of Biological Sciences Arkansas State University Jonesboro AR USA; ^5^ Department of Horticulture and Crop Sciences The Ohio State University Columbus OH USA

**Keywords:** adaptation, elevational gradient, landrace, maize, UV‐B

## Abstract

Globally, farmers cultivate and maintain crop landraces (i.e., traditional varieties). Landraces contain unique diversity shaped in part by natural and human‐mediated selection and are an indispensable resource for farmers. Since environmental conditions change with elevation, crop landraces grown along elevational gradients have provided ideal locations to explore patterns of local adaptation. To further probe traits underlying this differentiation, transcriptome signatures can help provide a foundation for understanding the ways in which functional genetic diversity may be shaped by environment. In this study, we returned to an elevational gradient in Chiapas, Mexico, to assess transcriptional differentiation of genes underlying UV‐B protection in locally adapted maize landraces from multiple elevations. We collected and planted landraces from three elevational zones (lowland, approximately 600 m; midland, approximately 1,550 m; highland approximately 2,100 m) in a common garden at 1,531 m. Using RNA‐seq data derived from leaf tissue, we performed differential expression analysis between maize from these distinct elevations. Highland and lowland landraces displayed differential expression in phenylpropanoid and flavonoid biosynthesis genes involved in the production of UV‐B protectants and did so at a rate greater than expected based on observed background transcriptional differentiation across the genome. These findings provide evidence for the differentiation of suites of genes involved in complex ecologically relevant pathways. Thus, while neutral evolutionary processes may have played a role in the observed patterns of differentiation, UV‐B may have also acted as a selective pressure to differentiate maize landraces in the region. Studies of the distribution of functional crop genetic diversity across variable landscapes can aid us in understanding the response of diversity to abiotic/biotic change and, ultimately, may facilitate its conservation and utilization.

## 1 INTRODUCTION

Genetic diversity is the material on which natural selection occurs. Crop landraces (i.e., traditional varieties) grown by farmers possess unique functional genetic diversity that is of great use, while also enhancing our understanding of evolutionary and agroecological processes. In situ (i.e., on‐site) conservation of landraces can contribute to long‐term agricultural sustainability in the face of change (Bellon et al., 2018; Isakson, 2014; Mercer, Perales, & Wainwright, 2012; Mercer et al., 2019; Olson, Morris, & Méndez, 2012; Perales, 2016; Vigouroux, Barnaud, Scarcelli, & Thuillet, 2011). In order to inform targeted in situ conservation strategies that maintain genetic diversity on the landscape, by and for farmers, we must better understand the distribution of functional or adaptive diversity across the landscape (e.g., Mercer & Perales, 2019). Such information may prove indispensable in empowering landrace farmers to identify and secure genetic resources pertinent to future growing conditions, especially in a time of climate change (Mercer & Perales, 2010). Crops such as maize (*Zea mays* ssp. *mays*) are well suited for this sort of study.

The symbiotic relationship between maize and humanity has aided in the movement of both species into diverse geographical locations that differ in latitude, elevation, climate, soil composition, ecosystem diversity, and human cultural practices (Brush & Perales, 2007; Grobman, Salhuana, & Sevilla, 1961; Mir et al., 2013; Perales, Benz, & Brush, 2005; Wellhausen, Roberts, Hernández, & Mangelsdorf, 1952). The spread of maize cultivation following its domestication in the lowlands of western Mexico ~9,000 years ago (van Heerwaarden et al., 2011; Matsuoka et al., 2002) led to the explosion of ~350 races (i.e., broad maize categories) in the Americas alone (Vigouroux et al., 2008). Maize expansion into these distinct geographical locations followed by geographical and cultural isolation has been a major driver in maize diversification (Brush & Perales, 2007; Orozco‐Ramírez, Ross‐Ibarra, Santacruz‐Varela, & Brush, 2016; Pressoir & Berthaud, 2004a; Ureta, González‐Salazar, González, Álvarez‐Buylla, & Martínez‐Meyer, 2013). This expansion required the evolution and diversification of morphological, phenological, and physiological characteristics of maize landraces and shaped their underlying functional genetic diversity (Mir et al., 2013; Navarro et al., 2017; Swarts et al., 2017; Vigouroux et al., 2008; Westengen, Berg, Kent, & Brysting, 2012).

Light in the ultraviolet‐B range (UV‐B; 280–320 nm) increases with increasing elevation (Godar, 2005; McKenzie, Johnston, Smale, Bodhaine, & Madronich, 2001) and increases of 5%–20% per 1,000 m increase in elevation have been documented (Lomax et al., 2012). Higher UV‐B exposure can negatively affect biological function and fitness in plants. Thus, UV‐B has the potential to act as a selective pressure to shape the genetic diversity in plants cultivated along elevational gradients (Sullivan, Teramura, & Ziska, 1992). UV‐B targets the photosynthetic machinery, proteins, membranes, and DNA of plants, which can affect plant development, physiological function and, ultimately, plant fitness (Frohnmeyer & Staiger, 2003; Gao et al., 2004; Jansen, Gaba, & Greenberg, 1998). Plants have evolved many mechanisms to reduce the deleterious effects of UV‐B, including DNA repair, free radical scavenging of reactive oxygen species (ROS), repair or replacement of photosynthetic machinery, and the generation of phenolic compounds such as flavonoids and hydroxycinnamic acids (HCAs) (Jansen et al., 1998; Sonbol et al., 2009). An increase in lignin content in leaf tissue following UV‐B exposure can occur in plants, which suggests that alterations to the cell wall and epidermal layer may be induced by and, ultimately, reduce the negative effects of UV‐B damage (Hilal et al., 2004; Rozema, Staaij, Björn, & Caldwell, 1997). It is plausible that maize landraces sourced along elevational gradients may be genetically differentiated at loci underlying these adaptations. Thus, studying their gene expression profiles when grown in a common garden setting, where the environmental contribution to phenotypic difference is reduced, may provide insight into the ways UV‐B shapes maize landrace diversity at a regional scale.

Of particular interest are the roles flavonoids and HCAs play in reducing the negative effects of UV‐B in plants. Flavonoids, a diverse group of specialized compounds derived from the phenylpropanoid biosynthesis pathway (Hahlbrock & Grisebach, 1979), have six main classes: anthocyanins, condensed tannins, chalcones, flavandiols, flavones, and flavonols. In addition, phlobaphenes, 3‐deoxyanthocyanins, and 3‐deoxy flavonoids are important in grasses (Winkel‐Shirley, 2001). Flavonoids perform a diverse array of functions in plants including pollinator attraction, symbiotic signaling, defense, male sterility, and UV‐B protection (Grotewold, 2006; Stafford, 1991; Winkel‐Shirley, 2001). In maize, flavonoids protect DNA from UV‐B damage (Casati & Walbot, 2003; Stapleton & Walbot, 1994). A notable study comparing highland maize landraces to a maize line deficient in anthocyanins identified increased levels of *C*‐glycosyl flavone content after UV‐B exposure in the former suggesting flavones aid highland landraces in coping with UV‐B (Casati & Walbot, 2005). Righini et al. (2018) demonstrated that *O*‐glycosyl flavones also play a role in reducing UV‐B damage and that maize contains at least two functional flavone synthase (*FNS*) genes. Additionally, two flavonol synthase (*FLS*) genes, the entry point into flavonol biosynthesis, have been described in maize and respond to UV‐B (Falcone Ferreyra, Casas, et al., 2012; Falcone Ferreyra et al., 2010). These findings in combination with the known protective role of flavonols against UV‐B damage (Emiliani, Grotewold, Ferreyra, & Casati, 2013) suggest they may be an important UV‐B protectant in maize. While several of these studies suggest that UV‐B may have shaped landrace diversity, without making comparisons to landraces from other elevations, we cannot discern the degree to which differentiation of genes related to UV‐B protection occurs across varied landscapes.

Like flavonoids, the HCAs also absorb UV‐B irradiance in many plants and are involved in protecting them against UV‐B damage (Burchard, Bilger, & Weissenböck, 2000; Graf, 1992; Lichtenthaler & Schweiger, 1998; Sheahan, 1996). In the grasses (Poaceae), HCAs are primarily composed of ferulates and *p*‐coumarates (Hatfield & Marita, 2010). However, caffeic acid and sinapic acids, and their derivatives, are also present in grasses to a lesser extent (Bouvier d’Yvoire et al., 2013; Hagerman & Nicholson, 1982). Grasses are the main group of plants to integrate HCAs into the cell wall (Hatfield & Marita, 2010); however, non‐cell wall bound HCAs have also been documented in grasses and have been shown to increase in plants experiencing increased UV‐B (Ruhland, Xiong, Clark, & Day, 2005). Several studies in grasses suggest HCAs shield UV‐B irradiance (Burchard et al., 2000; Lichtenthaler & Schweiger, 1998). The integration of HCAs into the cell wall of grasses can lead to cross‐linking of the cell wall matrix (Bunzel et al., 2003; Grabber, Ralph, Lapierre, & Barrière, 2004; Hatfield & Marita, 2010). This cross‐linking may lead to a UV‐B shielding effect by restricting the expansion of leaf cells (Ruhland et al., 2005) and, hence, make it more difficult for UV‐B light to penetrate and cause damage to mesophyll cells (Lichtenthaler & Schweiger, 1998). However, the presence of HCAs without flavonoids may be insufficient to eliminate DNA damage (Schmitz‐Hoerner & Weissenböck, 2003), suggesting a synergistic role of HCAs and flavonoids in preventing the negative effects of UV‐B on plant function. Thus, both flavonoids and HCAs are ideal candidates to study UV‐B induced transcriptional differences in maize landraces along elevational gradients.

In this study, we explore the differentiation of gene expression patterns of loci related to UV‐B protection in maize landraces originating along an elevational gradient in the state of Chiapas in southern Mexico. In past work in this system, we have found that these landraces tend to be locally adapted (Mercer, Martínez‐Vásquez, & Perales, 2008), in part due to underlying developmental, phenological (Mercer & Perales, 2019), and global transcriptional (Kost et al., 2017) differentiation. Here, we used RNA sequencing (RNA‐seq) data from 190 UV‐B protectant genes generated in Kost et al. (2017) from a common garden experiment to explore transcriptional differentiation among 15 landraces representing three distinct elevational zones (lowland, 600 m; midland, 1,550 m; highland, 2,100 m). As RNA is among the earliest of plant phenotypes, conducting hypothesis‐driven differential expression studies in common gardens can increase understanding of how evolutionary pressures and processes have shaped landrace genetic diversity.

This study allowed us to ask whether there are overarching patterns among landraces in their mRNA accumulation of genes involved in pathways that produce many UV‐B protectants, such as the general phenylpropanoid, flavonoid, lignin, and nucleotide‐sugar interconversion biosynthesis pathways. Furthermore, we were able to explore the degree to which differential expression patterns varied by elevation of landrace origin. Given current understanding about the influence of UV‐B light on plants, we anticipated many genes in these pathways to be DE in ways relevant to the UV‐B light intensity experienced by maize landraces from different elevational zones. Identified DE genes and the transcription factors regulating them may prove to be strong candidates for subsequent DNA‐based studies focused on unraveling the ways natural selection, neutral evolutionary processes, and phenotypic plasticity influenced the observed patterns of differential expression. This type of information will likely prove invaluable in crop landrace in situ conservation efforts and in the strategic acquisition of genetic materials by farmers and breeders to tolerate future climate conditions.

## 2 MATERIALS AND METHODS

### 2.1 Study location and germplasm collection

We carried out the field portion of our study in Chiapas, Mexico. The elevational drop between the central highlands of the Sierra Madre de Chiapas mountain range (~2,700 m) and the border of Guatemala (~600 m) is accompanied by abiotic and biotic gradients (Breedlove, 1978; Wolf & Alejandro, 2003). Chiapas is a center of diversity of maize and at least 21 races of maize have been collected over the years (Perales & Hernández‐Casillas, 2005). Crop races are defined as “…a group of related individuals with enough characteristics in common to permit their recognition as a group” (Anderson & Cutler, 1942, p. 71). These broad categories of diversity are assumed to be genetically similar and the term is often applied to the analysis of large maize groupings (e.g., Perales & Golicher, 2014). Elevation predicts the distribution of maize races in Chiapas (Brush & Perales, 2007; Perales & Hernández‐Casillas, 2005) and in the western highlands of Guatemala, south of our study location (van Etten & de Bruin, 2007; van Etten, López, Monterroso, & Samayoa, 2008). Landraces and races are not the same. A landrace is “… a crop population with historical origin, distinct identity and that lacks formal crop improvement; it is often genetically diverse, locally adapted and associated with traditional farming systems” (Perales & Golicher, 2014, p. 2; derived from Camacho Villa, Maxted, Scholten, & Ford‐Lloyd, 2005). Maize landraces all fall within a race, but some races consist of commercial varieties and landraces. Farmers of Chiapas maintain maize landraces in situ (Brush & Perales, 2007), even when “modern” germplasm is available, because landraces are locally adapted (Bellon & Brush, 1994; Mercer & Perales, 2019). Reciprocal common garden fitness studies employing maize landraces collected along the elevational gradient noted above have produced signals of local adaptation over multiple years (Mercer et al., 2008; Mercer & Perales, 2019).

In 2009, along our elevational transect, we collected nine maize landraces from within 100 m of each of three distinct elevations (27 landraces in total; highland: 2,100 m, midland: 1,550 m, and lowland: 600 m). Of the three elevations, the highland zone had the largest variation of collection elevations, reflected in the fact that two of the highland landraces had to be collected just outside that elevational band (Table 1). At each elevation, maize landraces were collected from three households in each of three communities. Each landrace consisted of a pooled sample of maize seed from 100 ears, which provided a representative sample of the diversity in the landrace. From these 27 landraces, we selected 15 for this study in such a way to maximize the diversity among landraces within a collection elevation, while also increasing the representation of landraces from areas where we planted common gardens (Table 1). Four maize races were represented: Comiteco, Olotillo, Olotón, and Tuxpeño*.* Lowland maize landraces were all Tuxpeño, midlands landraces were all Comiteco, and highland landraces consisted of one Olotillo and four Olotón (Table 1).

**Table 1:** Information on the 15 maize landraces from Chiapas, Mexico, used in this study

Elevation	Population ID	Municipality	Community	Latitude and longitude	Elevation (m)	Race
Lowland	1	Chicomuselo	Raizal	15.8939 N and 92.2537 W	648	Tuxpeño
	4	Frontera Comalapa	Benito Juárez	15.8229 N and 92.2042 W	563	Tuxpeño
	6	Frontera Comalapa	Benito Juárez	15.8229 N and 92.2042 W	563	Tuxpeño
	7	La Trinitaria	Juan Aldama	15.8554 N and 91.9377 W	598	Tuxpeño
	9	La Trinitaria	Nuevo Llano Grande	15.8390 N and 91.9363 W	595	Tuxpeño
Midland	10	La Trinitaria	El Rosario Tierra Blanca	16.0770 N and 91.7461 W	1,533	Comiteco
	12	La Trinitaria	Miguel Hidalgo	16.1056 N and 91.7780 W	1,524	Comiteco
	13	Comitán de Domínguez	San Francicsco El Ricón	16.2814 N and 92.1357 W	1,584	Comiteco
	17	Las Margaritas	Ignacio Zaragoza	16.3515 N and 91.9194 W	1,531	Comiteco
	18	Las Margaritas	Ignacio Zaragoza	16.3515 N and 91.9194 W	1,531	Comiteco
Highland	20	Comitán de Domínguez	Santa Elena Las Agujas	16.3612 N and 92.1792 W	2,089	Olotillo
	26	San Cristobal de Las Casas	Carrizal	16.6714 N and 92.6544 W	2,153	Olotón
	27	San Cristobal de Las Casas	Carrizal	16.6735 N and 92.6618 W	2,137	Olotón
	29	Teopisca	San Isidro Chichiuistán	16.5979 N and 92.5656 W	1,941	Olotón
	30	Teopisca	San Isidro Chichiuistán	16.6021 N and 92.5591 W	2,060	Olotón

Note

The 15 maize landrace populations used in this study, five populations from each of three elevational zones: highland zone (2,100 m), 1,941–2,153 m; midland zone (1,550 m), 1,524–1,584 m; and lowland zone (600 m), 563–648 m.

### 2.2 Common garden design and tissue collection

In 2011, as described in Kost et al. (2017), we planted a common garden, where we grew all 15 maize landraces together at the midland elevation (1,531 m; Quistajito, La Independencia; 16.3822 N and 92.0122 W). We planted seeds from the 15 landraces in a modified split‐plot design where main plots were elevation of landrace origin (highland, midland, or lowland) and randomized subplots were individual landraces from that given elevational zone (Figure S1). The planting together of landraces by elevation was necessary due to concerns that different adaptations among elevations could result in strong asymmetrical competition, thereby skewing RNA‐seq data. We planted each individual randomized landrace within its elevational main plot in a subplot row consisting of 10 *matas* (or planting locations), each of which contained three maize seeds for 30 seeds per landrace per block. We randomized the elevational main plots in each of three blocks (Figure S1). This common garden experimental design allowed us to identify transcriptional patterns specific to particular elevational zones due to genetic differences.

Over the course of the season, we collected leaf tissue five times; however, we only ran the first collection date (June 24, 2011) through RNA‐seq analysis due to differential insect herbivory later in the growing season. We collected leaf tissue between 9:00 a.m. and 1:00 p.m. from the youngest mature leaf with a collar (i.e., leaf collar method) from three randomly selected individuals per landrace, per block; the tissue was collected from plants at the V2 to V4 leaf stage. From this total of nine individuals per landrace, we immediately froze collected leaf tissue in liquid nitrogen, stored in a −80 C freezer until transport, and transported it in a liquid nitrogen dry shipper to ensure RNA integrity.

### 2.3 RNA extractions and RNA‐seq library preparation

As described in Kost et al. (2017), we performed RNA extractions with Qiagen RNeasy Plant Mini Kits and confirmed RNA integrity with the Agilent 2,100 Bioanalyzer. In total, we performed 135 RNA extractions (15 landraces × 9 individuals per landrace). To aid in RNA‐seq library construction, we assessed RNA concentrations using Qubit® 2.0 Fluorometer combined with the Qubit RNA Assay Kit. Before library construction, we pooled RNA from the three individuals sampled per landrace per block, resulting in 45 pooled RNA samples—one pooled sample per block × 15 landraces × three blocks. We performed RNA‐seq library construction using the strand‐specific library preparation method described in Zhong et al., (2011). Since we employed a multiplex sequence strategy, we assigned each library a barcode during library preparation. We randomized pooled RNA samples before library preparation to account for batch effects. Following library construction, we analyzed cDNA libraries using the Qubit DNA Assay Kit to determine concentration and we used the Agilent 2100 Bioanalyzer to determine library size.

### 2.4 Sequencing, library quality, trimming, and read mapping

We sequenced the 45 RNA‐seq libraries in four flow cell lanes of the Illumina HiSeq 2500 at the Genomics Resources Core Facility located at the Weill Cornell Medical College. We performed paired‐end sequencing at 50 bp. Since HiSeq 2500 normally produces ~120–130 million paired‐end reads per lane, we multiplexed 12 libraries per lane to produce ~10–11 million reads per library. In order to ensure balanced sequencing lanes, we ran 12 libraries in each lane. Since we only had 45 libraries, we sequenced one library in all four lanes, bringing the number of libraries in each lane to 12.

We employed a version of the Galaxy platform (Afgan et al., 2018) maintained by the Molecular and Cellular Imaging Center at the Ohio Agricultural Research and Development Center to visualize library quality and to preprocess reads for mapping to the maize genome. General library statistics and read quality were determined for each library before and after cleaning reads for mapping to the B73 RefGen_v2 5b.60 genome (Schnable et al., 2009) using *FastQC* version 0.10.1 (Andrews, 2010). To remove adaptor sequences and poly‐A/poly‐T tails, we used *cutadapt* version 0.9.5.a (Martin, 2011). Library adaptor sequences were removed using the 3′ adapters trim option with default settings selected with the exception of changing the minimum overlap setting to 6 bp. Poly‐A and Poly‐T tails were removed from libraries by selecting the 5′ or 3′ anywhere adapters option set to default with the exception of a 10 bp minimum overlap length. We then used *Trim the reads by quality* version 1.2.2 (Kofler et al., 2011) with default parameters specified except we altered the length threshold to 25 due to the short length (50 bp) of our original reads. Finally, we used *FastQC* to determine if Kmer content stabilized and to eliminate overrepresented sequences.

After confirming library quality, we mapped reads to the B73 RefGen_v2 5b.60 maize genome using *TopHat2* version 2.0.10 (Kim et al., 2013). We downloaded the maize B73 RefGen_v2 5b.60 maize genome from the Phytozome website (www.phytozome.net). We kept a majority of the *TopHat2* settings as default with the following exceptions. First, we set the mean inner distance between mate pairs and the standard deviation for distance between mate pairs to 150 bp. Second, we set the minimum and maximum intron lengths to 70 bp and 20,000 bp, respectively. Finally, we set the minimum and maximum intron length during split‐segment searches to 50 bp and 20,000 bp. We left mismatches set to the default setting of two. To ensure that mapping percentage did not differ among landraces from different elevations, which would indicate differences in divergence from the B73 RefGen_v2 5b.60 maize genome, we performed a second mapping run where we allowed zero mismatches. Any observed differences when allowing zero mismatches would indicate differential mapping, which would complicate data interpretation. Once we mapped reads to the maize B73 RefGen_v2 5b.60 maize genome, we used *htseq‐count* version 0.6.1 (Anders, Pyl, & Huber, 2015) in combination with the Phytozome gene‐specific gff3 annotation file to calculate the number of reads mapping to each gene. We then compiled the counts generated for each gene in each sample into a counts matrix, which we subsequently used to perform the differential expression analysis.

### 2.5 Differential expression analysis

We used *DESeq2* version 2.13 (Anders & Huber, 2010) to perform a multi‐factor differential global gene expression analysis. We imported raw counts for each library into *DESeq2* in the form of a counts matrix, and data were normalized using the median of ratios method. Since we had multiple factors in the experiment, elevation of landrace origin and block, we employed a multi‐factor model that included both factors. Our main interest was in how elevation of landrace origin influenced gene expression so we set elevation of landrace origin as the second factor in the equation enabling us to observe gene expression differences due to this factor. To be able to conduct pairwise comparisons among the three elevations of landrace origin, we used the “contrasts” function. We sorted DE gene sets for each pairwise comparison of elevation of landrace origin by false discovery rate (FDR) p‐values. We maintained loci having FDR *p*‐values of 0.05 or less; we considered them DE and retained gene lists containing DE genes for subsequent analysis. Observed differential expression among elevational zones is indicative of genetic differentiation, which could be due to adaptation or neutral evolutionary processes.

### 2.6 General phenylpropanoid, flavonoid, lignin, and nucleotide‐sugar interconversion analysis

To determine if elevation has shaped general phenylpropanoid, flavonoid, and lignin biosynthesis, as well as nucleotide‐sugar interconversion in the maize landraces in the region, we conducted a hypothesis‐driven analysis where we compared our DE gene lists to an established list of confirmed/predicted genes involved in the biosynthesis of phenolic compounds (Table S1). To generate a comprehensive list, we combined genes reported in the literature (Fornalé et al., 2010; Penning et al., 2009; Sekhon et al., 2011), at http://cellwall.genomics.purdue.edu, genes obtained by NCBI BLAST searches, and those recently identified in the literature. In addition, we identified several genes putatively involved in flavonoid biosynthesis while visualizing our data on the *MaizeCyc* metabolome (Monaco et al., 2013). We used specific maize gene names when appropriate, and for predicted or putative maize genes, we used general names and/or the naming scheme defined in Yang et al. (2017); predicted and putative gene names are described as such. In all, our gene list consisted of 190 genes (Table S1).

We employed three subsequent analyses approaches on the phenolic compound biosynthesis genes we identified as DE among distinct elevational zones to better understand the ways UV‐B irradiance along our study gradient may have influenced maize landrace transcriptomes in the region.

First, in order to gain an overall view of the expression profiles of these genes across the landraces and among elevational zones, we used *GENE‐E* from the Broad Institute (https://software.broadinstitute.org/GENE‐E/) to perform hierarchical clustering analyses on variance stabilized and normalized counts. We began by performing hierarchical clustering analysis on all 45 libraries using count data from 179 of the 190 genes from the full gene list; genes with no expression values across samples were excluded. Settings were adjusted to one minus Spearman rank correlation distances, 18 clusters, 1,000 sampling iterations, and average linkage, and clustering was visualized through heat map and tree generation. We also performed hierarchical clustering and heat map generation on the DE genes identified during the three pairwise comparisons of elevation origin. For these analyses, we used one minus Pearson correlation distances with remaining settings set to default to visualize the overarching patterns of gene co‐expression at the level of elevational zone of landrace origin. In addition to elucidating patterns of gene co‐expression, these latter heat maps provide the maize GRMZM gene IDs (Gene_Name) to facilitate the cross‐referencing of our results to the major maize databases (e.g., MaizeGDB) for those seeking additional gene‐specific information.

Second, we performed enrichment analyses to see if our phenolic biosynthesis gene set was overrepresented in our DE gene lists when compared to what would be expected when looking at all genes expressed in leaf tissue. Overrepresentation of focal gene sets in this type of analysis is consistent with, but does not confirm, that local adaptation may underlie the observed patterns. As our differential expression analysis was performed on leaf tissue spanning the V2 to V4 stages, we generated a list of genes expressed during these developmental stages. To do so, we accessed tissue‐specific expression data from the gene atlas presented in Hoopes et al. (2019); data accessed at maize.plantbiology.msu.edu. We focused on genes from the gene atlas that were expressed during the V1 to V5 stages. Utilizing the APGv3 to AGPv4 maize gene name conversion file, maize.v3tov4.geneIDhistory.txt (ftp.gramene.org), we refined the number of genes in the leaf background. Finally, we added genes that were DE in our elevational pairwise comparisons but that were absent in the leaf tissue data of the gene atlas.

In preparation for our enrichment analyses, we ensured the distributions of normalized gene counts of our leaf background gene set matched that of our phenolic biosynthesis candidate set by following the subsampling procedure described in Steige, Laenen, Reimegård, Scofield, and Slotte (2017). In preparation for subsampling, we removed genes showing no expression from our candidate gene list. We then divided the candidate gene list into 10 equally sized bins based on average expression levels over all landraces. Next, we identified the proportion of genes present in each bin. Before subsampling our leaf background gene list, we removed bins with two or fewer genes having >3,000 counts, 175 genes remained in our candidate gene list. We subsampled from the leaf background lists until the distributions had the same proportion of genes per bin as our candidate gene list. For each pairwise comparison, we included in the leaf background gene lists all of the differentially expressed genes for the comparison along with the phenolic candidate genes. We employed the statistical software R to conduct Yates’ chi‐square tests on the candidate gene and leaf background distributions to ensure they were the same. Finally, we generated a series of boxplots in the statistical software R to visualize changes in the distributions of our candidate gene and leaf background lists before and after subsampling.

We performed the enrichment analyses using Yates’ chi‐square tests in the statistical software R. In the analyses, we tested whether or not the phenolic compound biosynthesis gene set was enriched in our DE gene lists when compared to what would be expected in the leaf background. For example, in our highland–lowland experimental comparison, there were 29 DE genes from our phenolic compound biosynthesis gene list consisting of 175 genes (see below). In the highland–lowland comparison, there were 813 genes DE and the leaf background consisted of 11,656 genes (see below). Hence, we performed a Yates chi‐square test on the ratios of 175/11,656 to 29/813 to see if there were differences between what was observed and expected. We employed the same approach for the other two pairwise comparisons (highland–midland and midland–lowland).

Third, to visualize the pathway‐specific phenolic compound biosynthesis DE patterns among landraces from the three distinct elevational zones, we used *OMIX Visualization* (www.omix‐visualization.com). The three pathways visualized were general phenylpropanoid, flavonoid, and lignin biosynthesis. Our *OMIX Visualization* pathways consisted of genes underlying enzymatic steps and metabolic intermediates. Genes up‐regulated in highland landraces are represented by circles with blue while those up‐regulated in the lowlands are represented by red. A fully filled circle represents a log2fold change of 2.11 (i.e., expression level 4.32 times higher).

## 3 RESULTS

### 3.1 Library read counts and mapped reads

Raw read counts from the 45 RNA‐seq libraries ranged from approximately 5 million to 21 million per library with the majority of libraries containing nine million to 14 million reads (Table S2). *FastQC* analyses on raw reads confirmed reads were of high quality. Trimming adapter sequences and poly‐A/poly‐T tails and removing low‐quality bases eliminated overrepresented sequences and stabilized Kmer content without influencing read counts. After trimming reads, paired‐end reads ranged from five million to 20 million (Table S2).

All preprocessed RNA‐seq libraries mapped with high percentage to the RefGen_v2 5b.60 maize reference genome and the number of mapped reads per library was not influenced by elevation of landrace origin. With the exception of the second replicate of landrace six (i.e., sample 6.2), between 70 and 80 percent of the initial raw reads from each library uniquely mapped to the maize genome when we allowed a two base pair mismatch (calculated from Table S2 as uniquely mapped reads/raw reads). By comparison, approximately 66% of reads from lowland sample 6.2 mapped under those conditions. When we allowed zero mismatches, the library mapping percentage declined to 61–72 percent (calculated from Table S2 as uniquely mapped reads/raw reads) and average mapping percentages for each elevational zone did not differ significantly at a 0.05 alpha significance level (data not shown; highland, 66.87%; midland, 67.41%; lowland, 67.12%). Therefore, during the remainder of our analyses, we used results obtained from the two base pair mismatch mapping run, the default parameter value in *TopHat2*.

### 3.2 Hierarchical clustering of libraries

**Figure 1:**
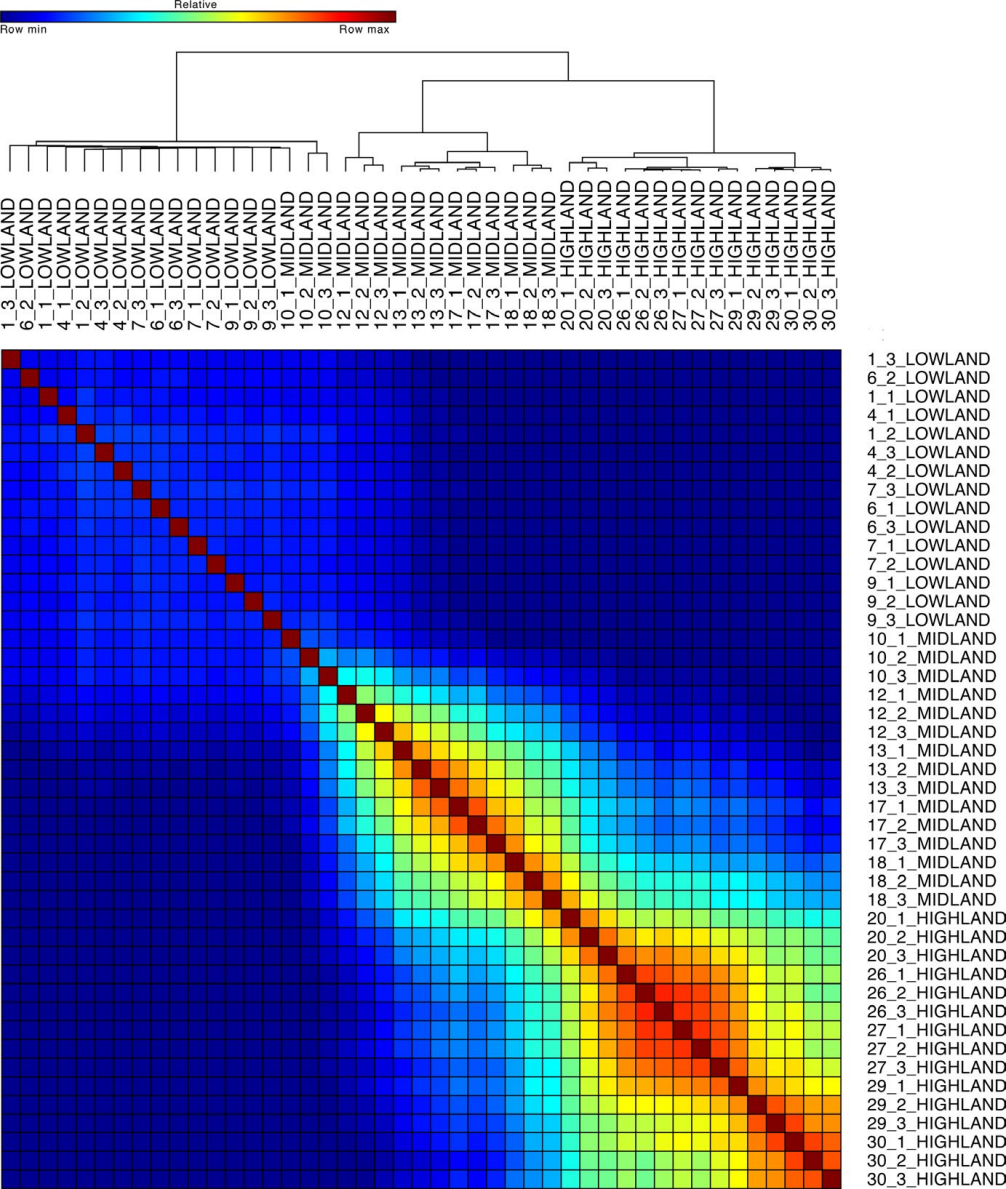
Hierarchical clustering of 45 maize landrace RNA‐seq libraries using gene expression of 179 genes encoding enzymes involved in phenolic compound biosynthesis. Consensus clustering heat map based on one minus Spearman rank correlation distances between all maize RNA‐seq libraries using 18 clusters, 1,000 resampling iterations, and average linkage on variance stabilized and normalized counts. The first number in each sample is the population and the second is the replicate. Elevation of population origin is specified. Dark red represents stronger correlation, while blue represents weaker correlations. Dendrogram above the heat map shows relations among RNA‐seq libraries

The hierarchical clustering analysis using 179 of the genes included in our phenolic compound biosynthesis enzyme gene list (Table S1) showed that elevation of landrace origin influenced overall expression patterns (Figure 1). Hierarchical clustering led to the formation of two primary branches: lowland landraces as one cluster and midland and highland as a second (Figure 1). The lowland cluster also included the three libraries from midland landrace 10. Within the lowland cluster, we identified two secondary clusters. The first consisted of two libraries from midland landrace 10, while the second was composed of all of the lowland landraces and the third library from landrace 10 (Figure 1). The second primary cluster, composed of midland and highland landraces, consisted of two secondary clusters. The first was composed of the remaining 12 midland libraries, while the second secondary cluster consisted of all 15 highland libraries. Importantly, highland landraces exhibited the strongest clustering (i.e., correlations) followed by those from midland environments. Comparatively, lowland landraces clustered more weakly (Figure 1).

### 3.3 Expression patterns and enrichment of phenolic compound biosynthesis genes

**Figure 2:**
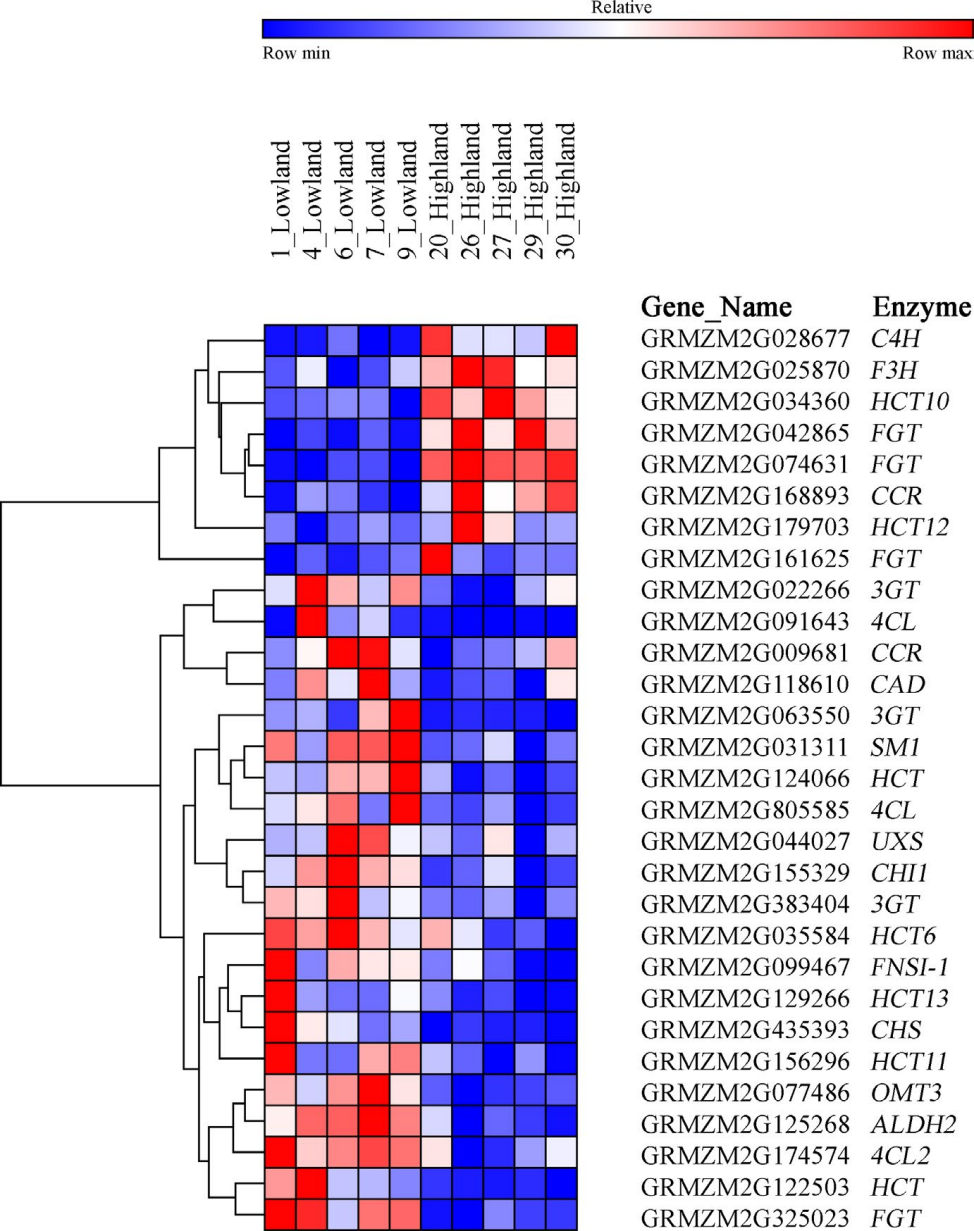
Highland and lowland landrace relative expression levels of normalized counts for differentially expressed (DE) phenylpropanoid, flavonoid, lignin, and nucleotide‐sugar interconversion genes. DE genes exhibited a FDR of 0.05 (Benjamini–Hochberg). Relative expression was determined for each row with red representing the population with the highest expression and blue representing the population with the lowest. We used the hierarchical clustering algorithm based on 1‐Pearson correlation distances to group genes with similar expression profiles across landraces. Enzyme gene names are as follows: *ALDH2,* aldehyde dehydrogenase 2; *CAD*, cinnamyl alcohol dehydrogenase; *CCR*, cinnamoyl‐CoA reductase; *CHI1*, chalcone isomerase; *CHS*, chalcone synthase; *C4H*, cinnamate‐4‐hydroxylase; *FGT*, flavonol glucosyltransferase; *FNSI‐1*, flavone synthase type 1; *F3H*, flavonoid‐3‐beta‐hydroxylase; *HCT,* hydroxycinnamoyl CoA transferase*; HCT6*, hydroxycinnamoyl CoA transferase 6; *HCT10*, hydroxycinnamoyl CoA transferase 10; *HCT11*, hydroxycinnamoyl CoA transferase 11; *HCT12*, hydroxycinnamoyl CoA transferase 12; *HCT13*, hydroxycinnamoyl CoA transferase 13; *OMT3,* Caffeoyl CoA O‐methyltransferase 3; *SM1*, salmon silk 1; *UXS,* UDP‐xylose synthase; *3GT*, flavonoid 3‐*O* glucosyltransferase; *4Cl*, 4‐coumarate: CoA ligase; *4CL2*, 4‐coumarate: CoA ligase 2

Forty‐nine of the 190 genes from our gene list were DE in at least one of the pairwise elevational comparisons. The comparison between highland and lowland landraces produced 29 DE genes (Figure 2; Table S3a), 18 genes were DE in the highland and midland comparison (Table S3b), and 18 genes were identified as being DE in the midland and lowland comparison (Tables S3c). Overarching patterns of gene co‐expression specific to elevational zones of landrace origin were observed during all three pairwise comparisons of DE genes (Figure 2 and Figures S2 and S3). For the highland–lowland comparison, a few salient patterns emerged. First, two main gene clusters formed that correlated with elevation of landrace origin. One consisted of eight genes that were consistently up‐regulated in the highland landraces while the remaining 21 genes were up‐regulated in the lowland landraces. Second, within each cluster were additional sub‐clusters that grouped transcriptional expression patterns. Finally, not all of the genes that we expected to be co‐expressed were—for example, three *FGT* genes grouped in the cluster up‐regulated in the highland landraces while the other *FGT* gene grouped in the cluster up‐regulated in the lowland landraces.

In preparation for our enrichment analyses, we determined that the leaf background gene lists for each of the three pairwise comparisons, before subsampling, consisted of around 29,000 genes (Table S4). The leaf background for the three pairwise comparisons was as follows: (a) highland–lowland, 28,867; (b) highland–midland, 28,681; (c) midland–lowland, 28,763 (see Table S4 for calculation details).

During subsampling, we determined a majority of the candidate genes in our experimental list were assigned to the first bin (i.e., fewest counts) and that the number of genes in the leaf background for all three pairwise comparisons was substantially reduced. The average normalized counts for the 179 candidate genes across all landraces ranged from 0.02 to 7,600, 156 (89%) of the candidate genes were located in the first bin (0.02–760.01 counts), and 15 (9%) were in the second bin (760.01–1,520 counts; Table S4). The reduction in the number of leaf background genes for all pairwise comparisons was influenced by genes not falling into the candidate gene defined bins. Approximately 1,500 genes from each comparison had counts outside of the defined bins (data not shown). Subsampling further reduced the leaf background lists, for bins one through four, from 27,073 to 11,656 in the highland–lowland comparison, 26,887 to 11,656 in the highland–midland comparison, and from 26,968 to 11,656 in the midland–lowland comparison (Table S4). Our chi‐square test comparing our newly formed leaf background distribution to that of the phenolic candidate gene list demonstrated that the distributions were the same (Table S5; Chi‐square value 3.725e‐05, *p*‐value 1). This similarity in distributions can be seen in the spread of data before and after subsampling (Figures S4 and S5).

Our enrichment analyses demonstrated that genes involved in biosynthesis of UV‐B protectants were overrepresented or enriched in the DE gene lists obtained during all pairwise elevational zone comparisons when compared to what would be expected in the leaf background (Table 2; highland–lowland *p*‐value .000023, highland–midland *p*‐value .028, midland–lowland *p*‐value .046).

**Table 2:** Enrichment analyses of differentially expressed maize phenolic compound biosynthesis genes

Comparison	Chi‐square statistic	*p*‐value	Background	DE list	Enriched
Highland–lowland	17.94	.000023	175/11656	29/813	Yes
Highland–midland	4.84	.028	175/11656	18/669	Yes
Midland–lowland	4.00	.046	175/11656	18/703	Yes

Note

Enrichment analyses for phenolic compound biosynthesis for all three pairwise comparisons (highland–lowland; highland–midland; and midland–lowland). *p*‐values <.05 signify that the DE gene list for that particular pairwise comparison was enriched for overall phenolic compound biosynthesis. Background column—number of genes in the phenolic biosynthesis gene list/ number of genes expressed in the maize genome. Both of these numbers were after adjusting for leaf tissue specificity and gene expression distribution (see Section 2). DE list column—number of phenolic compound biosynthesis genes DE/ total number of genes DE for that comparison. Enriched (yes/no) column—describes if the corresponding pairwise comparison was enriched. Chi‐square statistic *p*‐values were after Yates correction.

### 3.4 Genes involved in general phenylpropanoid, anthocyanin, and lignin biosynthesis

**Figure 3:**
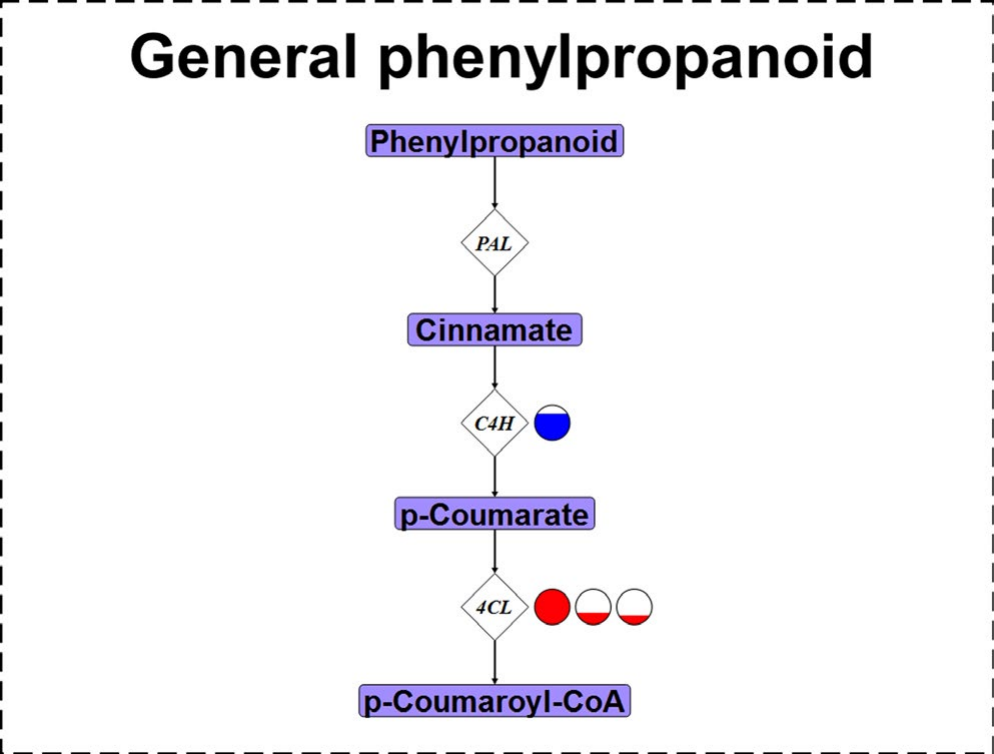
Differentially expressed genes between highland and lowland maize landraces encoding enzymes involved in general phenylpropanoid biosynthesis. The general phenylpropanoid biosynthesis pathway consisting of three genes underlying enzymatic steps (white diamonds) and four metabolic intermediates (light purple rectangles). Enzyme gene names are as follows: *PAL*, phenylalanine ammonia lyase; *C4H*, cinnamate‐4‐hydroxylase; *4Cl*, 4‐coumarate: CoA ligase. Genes up‐regulated in highland landraces are represented by circles with blue while genes up‐regulated in lowland landraces are represented by red. A full circle represents a log2fold change of 2.11 (i.e., 4.32 times higher expression); partially filled circles represent up‐regulation to a portion of 4.32 times. If multiple circles are present to the right of an enzyme gene name, then multiple genes encoding that type of enzyme are up‐regulated—cross‐reference Figure 2 and Table S3 for associated gene names and log2fold change values, respectively. Genes were considered differentially expressed when significant at a 0.05 FDR rate using a *Benjamini–Hochberg correction*

Highland and lowland landraces exhibited differential expression for several genes encoding enzymes involved in general phenylpropanoid biosynthesis (Figures 2 and 3). A putative *C4H* gene was highly expressed in highland landraces when compared to lowland landraces (Figures 2 and 3, Tables S3a, S6a, and S7a). Similarly, the same *C4H* gene was more highly expressed in midland than in lowland landraces (Figures S3 and S9, Tables S3c, S6c, and S7c), but expression was equivalent in the highland and midland landraces (Figures S2 and S6, Tables S3b, S6b, and S7b). Three genes putatively encoding 4‐coumarate: CoA ligases (4CLs) were more highly expressed in lowland landraces when compared to those from the highlands (Figures 2 and 3). In the midland–lowland and highland–midland comparisons, landraces originating from the lower of the two elevations also showed higher expression of putative *4Cl* genes (Figures S2, S3, S6, and S9).

**Figure 4:**
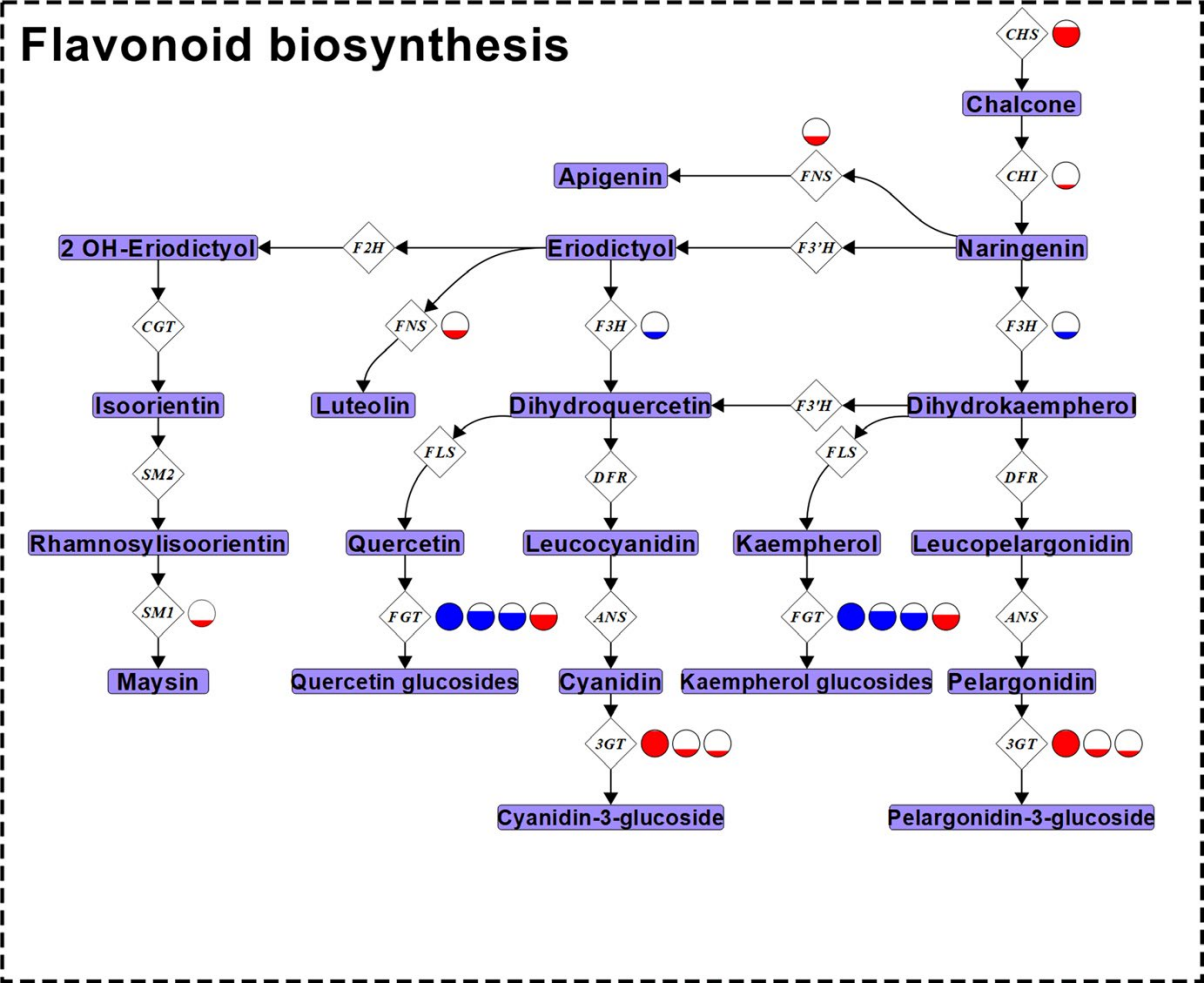
Differential expressed genes between highland and lowland maize landraces encoding enzymes involved in flavonoid biosynthesis. The flavonoid biosynthesis pathway for maize including genes underlying enzymatic steps (white diamonds) and metabolic intermediates (light purple rectangles). Multiple circles next to an enzyme gene name represent multiple genes encoding that enzyme type—cross‐reference Figure 2 and Figure S3 for associated gene names and log2fold change values, respectively. *p*‐coumaroyl‐CoA, the final product in the general phenylpropanoid biosynthesis pathway (Figure 3), is the entry point into the flavonoid biosynthesis pathway. Anthocyanins—cyanidin and pelargonidin; *C*‐glycosyl flavones—maysin; *O*‐glycosyl flavones—apigenin and luteolin; flavonols—kaempferol and quercetin. Genes were considered differentially expressed when significant at a 0.05 FDR rate using a *Benjamini–Hochberg correction.* Enzyme genes are as follows: *CHS*, chalcone synthase; *CHI*, chalcone isomerase; *F3H*, flavonoid‐3‐beta‐hydroxylase; *FNS*, flavone synthase *F3’H*, flavonoid‐3’‐hydroxylase; *DFR*, dihydroflavonol reductase; *FLS*, flavonol synthase; *FGT*, flavonol glucosyltransferase; *ANS*, anthocyanidin reductase; *3GT*, flavonoid 3‐*O* glucosyltransferase; *F2H*, flavanone‐2‐hydroxylase; *CGT*, *C*‐glycosyltransferase; *SM2*, salmon silk 2; *SM1*, salmon silk 1. Genes up‐regulated in highland landraces are represented by blue and those up‐regulated in lowland landraces are represented by red. Full circles represent a log2fold change of 2.11 (i.e., 4.32 times higher expression); partially filled circles represent up‐regulation to a portion of 4.32 times

Highland and lowland landraces displayed differential expression for several genes encoding enzymes involved in the initial steps of flavonoid biosynthesis and the subsequent steps of anthocyanin production (Figures 2 and 4). A gene putatively encoding a chalcone synthase (CHS) displayed higher expression levels in the lowland landraces (Figures 2 and 4). In addition, a chalcone isomerase (*CHI*) and a gene putatively encoding a flavonoid‐3‐beta‐hydroxylases (F3H) were DE between highland and lowland landraces. The former showed greater expression in the lowland landraces while the later showed greater expression in landraces from the highlands (Figures 2 and 4). Three genes predicted to encode flavonoid 3‐*O*‐glucosyltransferases (3GT) also showed greater expression in the lowland landraces when compared to highland landraces (Figures 2 and 4). When comparing highland and midland landraces or midland and lowland landraces, no genes encoding CHS and CHI enzymes were DE (Figures S7 and S10). However, a gene encoding a putative F3H enzyme showed greater expression in midland landraces when compared to those from the highlands. Two putative *3GT* genes displayed greater expression in lowland landraces when comparing with those from the midlands (Figures S3 and S10). When we compared highland landraces to midland landraces, one predicted *3GT* gene was more highly expressed in the highland landraces (Figures S2 and S7). In addition, we observed greater expression of a putative leucoanthocyanidin dioxygenase (*LDOX*) gene in the midland landraces when compared to those from the highlands (Figures S2 and S7).

**Figure 5:**
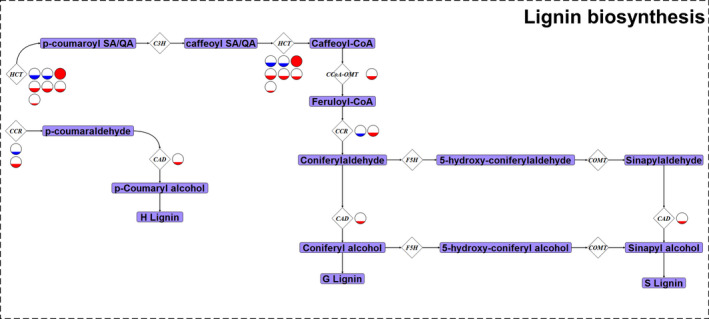
Differentially expressed genes between highland and lowland maize landraces encoding enzymes involved in lignin biosynthesis. The lignin biosynthesis pathway for maize including metabolic intermediates (light purple rectangles) and genes underlying enzymatic steps (white diamonds). *p*‐coumaroyl‐CoA, the final product in the general phenylpropanoid biosynthesis pathway (Figure 3), is the entry point into the lignin biosynthesis pathway both through the p‐coumaroyl SA/QA and *p*‐coumaraldehyde routes. Enzyme gene names are as follows: *HCT*, hydroxycinnamoyl CoA transferase; *CAD*, cinnamyl alcohol dehydrogenase; *C3H*, coumarate‐3‐hydroxylase; *CCoA‐OMT*, caffeoyl‐coenzyme A O‐methyltransferase; *CCR*, cinnamoyl‐CoA reductase; *F5H*, ferulate 5‐hydroxylase; *COMT*, cinnamate‐4‐hydroxylase. Full circles represent log2fold changes of 2.11 (i.e., 4.32 times higher expression); partially filled circles represent up‐regulation to a portion of 4.32 times. Enzyme gene names with multiple circles next to them represent multiple genes encoding that type of enzyme—cross‐reference Figure 2 and Table S3 for associated gene names and log2fold change values, respectively. Genes were considered differentially expressed when significant at a 0.05 FDR rate using a *Benjamini–Hochberg correction*. The *CCoA‐OMT* differentially expressed was *OMT3*

A majority of the lignin biosynthesis genes DE between highland and lowland landraces were up‐regulated in the lowland landraces. Five of the seven DE genes putatively encoding hydroxycinnamoyl CoA transferases (HCT), one of the two DE genes putatively encoding cinnamoyl‐CoA reductase (CCR), one putatively encoding a cinnamyl alcohol dehydrogenase (CAD), and a gene putatively encoding a caffeoyl‐coenzyme A *O*‐methylase (OMT), were all highly expressed in the lowland landraces when compared to those from the highlands (Figures 2 and 5). We observed a similar pattern when we compared midland landrace lignin gene expression patterns to those from the lowland (Figures S3 and S11). The opposite was observed in the highland–midland comparison where all DE lignin genes except for one *HCT* gene were more highly expressed in the highland landraces (Figures S2 and S8). In addition to the observed lignin gene expression patterns, we observed greater expression of coniferyl aldehyde dehydrogenase (*ALDH*) genes in both lowland and midland landraces when compared to those from highland locations (Figure 2 and Figure S2). No differential expression of *ALDH* genes was observed when comparing midland and lowland landraces. ALDHs, while not involved in lignin biosynthesis, can increase HCA levels out of the lignin pathway.

### 3.5 Genes involved in flavone and flavonol biosynthesis

We observed differential expression of a flavone synthase gene (*FNSI‐1*) (PMID: 26269546) involved in *O*‐glycosyl flavone biosynthesis when comparing highland landraces to those collected from midland and lowland elevations. In both comparisons, landraces from the lower elevations displayed greater expression of this gene (Figure 2 and Figure S2 and S3). When comparing midland and lowland landraces, we observed higher expression of a flavone synthase gene (*FNSI‐2*) putatively involved in *O*‐glycosyl flavone biosynthesis in the midland landraces (Figure S3). In addition, lowland landraces displayed greater expression of (*SM1*) (PMID: 27221383) when compared to those from the highlands (Figures 2 and 4)—*SM1* is involved in the last step of *C*‐glycosyl flavone (maysin) biosynthesis.

We also observed differential expression in the flavonol biosynthesis pathway among landraces from different elevational zones. When comparing highland to lowland landraces, three of the four DE genes encoding putative flavonol glucosyltransferases (FGT) showed increased expression in the highland landraces (Figures 2 and 4). When comparing expression between the highland and midland landraces, we identified an *FLS* gene (*FLS2*) (PMID: 22654889) more highly expressed in highland landraces and a predicted *FGT* gene more highly expressed in the midland landraces (Figures S2 and S7). The comparison between midland and lowland landraces also showed *FLS2* as DE, but this time it was more highly expressed in lowland landraces. Additionally, two genes predicted to encode *FGT* genes were highly expressed in the midland landraces when compared to those from the lowlands (Figures S3 and S10). Upon further investigation, we found lowland landrace six to have a *FLS2* expression level between two and five times greater than the other four lowland landraces and the second highest expression level of all landraces included in the study (Table S6c), which may have influenced all *FLS2* comparisons with lowland landraces.

### 3.6 Genes involved in nucleotide‐sugar interconversion

Lowland landraces showed higher expression levels of genes encoding nucleotide‐sugar interconversion enzymes when compared to highland landraces. One gene (*SM1*) (PMID: 27221383) encoding a rhamnose synthase (RHM) and another gene encoding a putative UDP‐D‐xylose synthase (UXS) were both more highly expressed in lowland landraces when compared to those from the highlands (Figure 2). As mentioned, *SM1* plays a role in maysin biosynthesis, but can also play a role in cell wall biosynthesis. We observed a similar trend of landraces from lower elevations showing greater expression of nucleotide‐sugar interconversion genes when comparing highland and midland landraces; both a gene encoding a putative RHM and a putative UDP‐D‐glucose dehydrogenase (*UGD*) were more highly expressed in the midland landraces (Figure S2). However, when comparing midland to lowland landraces, we observed greater expression of a putative *RHM* gene in the midland landraces (Figure S3).

## 4 DISCUSSION

An extensive body of literature has documented maize landrace genetic diversity; however, few studies have documented the distribution of functional genetic diversity in maize landraces across the landscape at a regional scale. Here, we employed RNA‐seq on maize landraces collected along an elevational gradient in Chiapas, Mexico, but grown at an intermediate elevation. We determined that highland, midland, and lowland landraces exhibited transcriptional differentiation in the general phenylpropanoid, flavonoid, and lignin biosynthesis pathways, as well as in genes involved in nucleotide‐sugar interconversion—all of which contribute to UV‐B protection. Hierarchical clustering suggested that highland and midland maize landraces are more like each other than they are to lowland landraces for this functional diversity. Our differential expression and enrichment analyses of landraces from different elevational zones indicate that expression differences are consistent with UV‐B acting as an agent of natural selection along our study elevation gradient, though neutral processes could also be involved. To our knowledge, this is a unique case of documenting differentiation across a complex pathway within a wild or cultivated plant species along a natural environmental gradient.

### 4.1 UV‐B related landrace hierarchical clustering patterns

Overall, highland and midland landraces were more similar to each other in their phenolic compound biosynthesis expression profiles than they were to the lowland maize landraces. This greater relatedness among highland and midland landraces was also observed when comparing expression patterns of the 500 most variable genes in the genomes of these landraces in a related study (Kost et al., 2017). In the hierarchical clustering analysis, landraces from higher elevations grouped more tightly. This could possibly be due to high UV‐B levels or other stresses and/or reduced seed exchange (Bellon, Hodson, & Hellin, 2011) leading to increased selection and local adaptation as maize was moved from its domestication center in the lowlands of southwestern Mexico to higher elevations. All midland landraces in our study were Comiteco and four out of the five highland landraces were Olotón; all tightly grouped together. These clustering results may corroborate the proposed evolutionary origin of Comiteco being from the hybridization of maize races Tehua and Olotón (Wellhausen et al., 1952). Tuxpeño has been described as more distantly related to Comiteco and Olotón *(*Ruiz Corral et al., 2008; Sanchez, Goodman, & Stuber, 2000), which corresponds with the lowland landraces (Tuxpeño) grouping together separately. Our finding suggests that the differentiation of pathways involved in UV‐B protection among the landraces we studied here may actually parallel differentiation of the races from which they come and the adaptation of those races to local conditions as maize expanded into higher elevations following domestication.

Research on the quantitative genetic differentiation of maize from these elevations in Chiapas does not necessarily coincide cleanly with these findings since midland and lowland landraces have sometimes been shown to be more similar in their fitness values than highland landraces (Mercer et al., 2008) and, in other years and environments, midland and highland types have been most similar (Mercer & Perales, 2019). This suggests that either: (a) year‐to‐year and location‐to‐location environmental variation may influence the expression of phenotypes and fitness, or (b) additional strong selective pressures acting on other pathways may have also contributed to the geographical structuring of maize landraces in Chiapas.

### 4.2 Transcriptional differentiation of UV‐B protectants

In common gardens, where environmental components of plant phenotypes are minimized, phenotypic differences (e.g., DE genes) are indicative of genetic differentiation (Frei, Scheepens, Armbruster, & Stöcklin, 2012; Hufford & Mazer, 2003; Linhart & Grant, 1996). Any observed differentiation could be due to adaptation and/or neutral evolutionary processes (Hufford & Mazer, 2003). However, performing enrichment analyses on DE genes obtained during hypothesis‐driven common garden experimentation can suggest molecular pathways and cellular and biological processes (e.g., UV‐B protectants) potentially under selection. This is because variance in selection intensities along environmental gradients can differentiate polygenic traits in populations along the gradient (Berg & Coop, 2014; McKay & Latta, 2002; Pritchard & Di Rienzo, 2010; Savolainen, Lascoux, & Merilä, 2013). This differentiation can manifest as an enrichment of DE genes underlying the trait(s) of interest due to common transcriptional regulation and, hence, gene co‐expression (Aguilar‐Rangel et al., 2017; Kost et al., 2017; Swanson‐Wagner et al., 2012; Yang et al., 2017). In this study, all pairwise comparisons among landraces from our three different elevational zones were clearly enriched for phenolic compound biosynthesis genes. Thus, our enrichment analyses findings are consistent with the idea that selection by factors, such as UV‐B irradiance, may well have shaped the phenolic compound biosynthesis pathways of maize landraces along our study elevational gradient in Chiapas. Observed patterns of gene co‐expression provide a foundation to begin exploring the ways that differential transcriptional regulation may underlie these apparent patterns of local adaptation. Nevertheless, further study would be necessary to differentiate how neutral and adaptive evolutionary processes may have worked together to shape differentiation of these pathways.

Differences in transcriptome profiles within these complex pathways among the highland and lowland landraces suggest that maize along our study gradient may employ distinct mechanisms to ward off the deleterious effect of UV‐B irradiance. Plants can increase lignin deposits and thickness in the epidermal layer following UV‐B exposure to reduce potential UV‐B damage (Day, 1993; Hilal et al., 2004; Hutzler et al., 1998; Rozema et al., 1997). Anthocyanins and flavones can also play a protective role against UV‐B (Casati & Walbot, 2005; Gould, McKelvie, & Markham, 2002; Holton & Cornish, 1995; Stapleton & Walbot, 1994). When grown in a midland environment (1,531 m), the lowland maize landraces showed higher expression of several confirmed and putative genes involved in anthocyanin, flavone, and lignin biosynthesis as compared to highland landraces. Lowland landraces also displayed greater gene expression of putative *4Cl* genes, a putative *CHS* gene, and *CHI1*, all of which increase the amount of substrate entering flavonoid and lignin biosynthesis. Finally, the increased expression of two putative nucleotide‐sugar interconversion genes in the lowland landraces when compared to the highlands (*SM1* and *UXS*), which potentially encode enzymes involved in generating sugar building blocks for carbohydrates bound for the cell wall (Penning et al., 2009), further suggests that differences in UV‐B irradiance levels along our study gradient may have led to alterations in cell wall biosynthesis in the maize landraces. Combined, these findings confirm differentiation of the modes of UV‐B protection employed between elevations through differentiation of these complex anthocyanin, flavone, and cell wall biosynthesis pathways. However, further work would be required to confirm adaptive differentiation of these genes.

In comparison with lowland landraces, highland maize landraces showed greater expression of genes putatively involved in flavonol and HCA biosynthesis, further suggesting that maize landraces in the region are transcriptionally differentiated in key pathways involved in reducing the negative effects of UV‐B irradiance. Our finding of greater expression of several putative *FGT* genes likely involved in flavonol glycoside formation in the highland when compared to lowland landraces, in combination with the strong body of literature discussing the role of flavonols in UV‐B protection (Falcone Ferreyra et al., 2010; Kusano et al., 2011; Li, Ou‐Lee, Raba, Amundson, & Last, 1993; Ryan, Swinny, Markham, & Winefield, 2002), suggests that variance in UV‐B intensity along our study gradient may underlie the observed differential expression of genes related to flavonol glycosides biosynthesis in landraces from different elevational zones. Several putative *FGT* genes being DE in the midland–lowland and highland–midland comparisons provide further support. By contrast, we did not see increased expression of any documented *FLS* genes among highland and lowland landraces, which might be expected if flavonol‐related transcriptional differentiation was somehow adaptive. But the formation of specific flavonol glycosides can increase the UV‐B protecting and scavenging activities of flavonols (Graham, 1998; Harborne & Williams, 2000; Neugart et al., 2012); flavonol quality is as important as quantity. The identification of increased *FLS* (i.e., *FLS2*) gene expression in the highland landraces when compared to those from the midlands suggests that the quantity of flavonols may also be relevant in counteracting the detrimental effects of UV‐B intensity in the region.

As is the case with flavonols, HCAs have been ascribed a role in UV‐B protection (Burchard et al., 2000; Lichtenthaler & Schweiger, 1998; Ruhland et al., 2005). Greater expression of a putative *C4H* gene in the highland landraces and increased expression of putative *4Cl* genes in the lowland landraces suggests that highland and lowland maize landraces could be genetically differentiated in HCA biosynthesis. We observed a similar trend when comparing midland and lowland landraces—greater expression of a putative *C4H* gene in midland landraces and increased expression of a putative *4Cl* gene in lowland landraces. Since 4Cl enzymes are needed to convert HCAs to lignin, increased expression of genes encoding these enzymes in the lowland landraces suggests that the regulatory mechanisms underlying the conversion is different in landraces from different elevational zones (Humphreys & Chapple, 2002). We would expect, then, that in the midland common garden environment, HCA production would have likely been relatively higher in highland and midland landraces. In addition, we observed expression difference in *ALDH2* when comparing highland and lowland landraces. ALDH2 is responsible for converting coniferyl aldehyde and sinapyl aldehyde into their corresponding HCAs—ferulic and sinapic acid, respectively (Nair, Bastress, Ruegger, Denault, & Chapple, 2004; Vanholme et al., 2012). These findings suggest that the control of HCA biosynthesis is differentiated in the maize landraces of Chiapas which may influence the UV‐B protection that maize from different elevations enjoy from HCAs.

The apparent differentiation of flavonol and anthocyanin biosynthesis among highland and lowland landraces may be of particular interest. Studies in Arabidopsis and maize have documented that an increase in flavonol biosynthesis can coincide with a decrease in anthocyanin biosynthesis (Falcone Ferreyra et al., 2010; Fornalé et al., 2013; Owens et al., 2008), consistent with a potential trade‐off. In our system, highland landraces showed increased expression of putative flavonol biosynthesis genes, while lowland landraces showed increased expression of several genes putatively involved in anthocyanin biosynthesis. Thus, highland and lowland landraces may be differentiated in the compounds that protect them from UV‐B. While we cannot say for certain that such differentiation is adaptive as neutral processes could also result in this kind of differentiation, it is important to note that anthocyanins provide less UV‐B protection than flavonols and one might expect less protection required at lower elevations. Given the other functions of anthocyanins (e.g., coloring, insect defense), there may be other selective pressures (e.g., human selection or insect herbivory) at lower elevations that could favor anthocyanin production, as well.

This potential trade‐off is particularly interesting in light of the study by Casati and Walbot (2005) who identified *C*‐glycosyl flavones (maysin), and not flavonols, to be involved in the ability of highland maize landraces from Mexico (2,200–2,800 m) and the Andes (2,200–3,900 m) to cope with increased UV‐B when compared to inbred lines. Our findings are like those of Casati and Walbot (2005) in that they suggest powerful flavonoids (i.e., flavonols, rather than anthocyanins) may have been selected for in particular elevational zones in Chiapas. In addition to flavonols, the lowland landraces studied here showed increased expression of *SM1,* a gene encoding *C*‐glycosyl flavones (maysin), when compared to highland landraces, indicating landraces from particular elevational zones are also transcriptionally differentiated for *C*‐glycosyl flavones (maysin) production. Here, we also observed the differential expression of *FNSI‐1* between highland and lowland landraces (i.e., up‐regulated in lowland landraces), a gene involved in *O*‐glycosyl flavone (apigenin) biosynthesis; FNSI‐1 has been shown to protect against UV‐B induced damage when expressed in Arabidopsis (Righini et al., 2018). The transcriptional differentiation of loci involved in the biosynthesis of apigenin and maysin flavones and flavonols in the landraces along our study gradient is consistent with UV‐B irradiance being an important selective pressure shaping diversity in the region. Increased expression of *FNSI‐1* in midland landraces when compared to those from the highland, and of *FNSI‐2* in midland landraces when compared to those from the lowlands, further supports this claim.

Apparent differences between our study and the findings reported in Casati and Walbot (2005) are likely influenced by three main factors. First, we did not provide UV‐B light supplementation, which was done in Casati and Walbot (2005). As pointed out by the authors, increased levels of maysin were only observed in highland landraces grown in lowland condition in the presence of UV‐B supplementation. Thus, the response was induced. Our highland landraces experienced reduced UV‐B levels in the midland environment relative to their home environment and, hence, we may not have expected to see heightened *C*‐glycosyl flavone production unless it was constitutively expressed rather than induced by high UV‐B. Second, other selective pressures may have shaped the phenolic biosynthesis pathway of lowland landraces in our study. As *C*‐glycosyl flavones retard the growth of lepidopteran pests (Waiss et al., 1979), it is possible that lowland landraces have been selected for constitutive expression of *C*‐glycosyl flavone to minimize insect herbivory. Finally, lowland landraces may not have the capacity to induce flavonols and instead flavone production is increased when they experience higher than normal levels of UV‐B light, which would have been the case in a midland environment. Combined, these observations suggest that there may not be fixed patterns of how maize landraces from different regions adapt or respond to UV‐B and that other selective pressures they encounter can also shift their gene expression profiles.

### 4.3 Genetic drift, phenotypic plasticity, and selective pressures

Importantly, some of the differential expression we observed in this study could have been caused by genetic drift and not local adaptation. A novel study by Kryvokhyzha et al. (2016) suggests that the use of a correction for population structure in differential expression studies can remove genes affected by genetic drift from resulting DE lists. However, they also caution that such a correction may eliminate true signals of local adaptation through false negatives (Kryvokhyzha et al., 2016). As we did not have the genetic data necessary to correct for population structure in this study, and acquiring accurate data from our pooled RNA samples would have been problematic due to small pool sizes, population diversity, and potential unbalanced RNA contributions and variation in allele‐specific expression (Hoban et al., 2016; Schlötterer, Tobler, Kofler, & Nolte, 2014; Serre et al., 2008), we cannot know the extent to which genetic drift might be the causative factor influencing differential expression. However, given that the geographical region where our maize landraces were collected was small, the outcrossing nature of maize, the established and dynamic seed exchange networks present in the region, and the fact that farmers in the region are interested in cultivating different and new landraces (Arteaga et al., 2016; Bellon & Brush, 1994; van Etten et al., 2008; van Heerwaarden et al., 2011; Louette, Charrier, & Berthaud, 1997; Louette & Smale, 2000; Perales et al., 2005; Pressoir & Berthaud, 2004a, 2004b; Warburton et al., 2011), genetic drift may well be less pronounced than both natural and human‐mediated selection in the region. Furthermore, the enrichment analyses results suggest that the observed patterns of transcriptional differentiation in our focal pathways are greater than would be expected by chance. Thus, our differential expression patterns appear consistent, at least in part, with differential adaptation. As our ability to correct for population structure in transcriptomic data advances, studying local adaptation in crop landraces and natural population will be enhanced as we may be able to add to the approach employed here to more firmly separate transcriptional signals due to adaptation and those due to neutral evolutionary processes.

We identified DE genes involved in the biosynthesis of phenolic compounds relevant to UV‐B protection, which appear to indicate genetic differentiation and potential signals of natural selection. However, given that our data were generated under only one environmental condition, we were unable to determine the extent that phenotypic plasticity may have been affecting our findings. Phenotypic plasticity, the ability of an organism to alter its phenotype in different environments (Sultan, 2000), allows sessile organisms (e.g., plants) to cope with environmental change (Grenier, Barre, & Litrico, 2016; Nicotra et al., 2010). Since plasticity has a genetic basis, it can contribute to the adaptation of plant populations to new and changing environment (Bradshaw, 2006; Ghalambor et al., 2015; Lande, 2009). One approach to elucidate signals of phenotypic plasticity in nature is to perform reciprocal transplant studies where populations collected along a gradient are grown in native and non‐native common gardens along the gradient (Palacio‐López, Beckage, Scheiner, & Molofsky, 2015; Pigliucci, 2001), that is, highland, midland, and lowland environments in our study. Variance in phenotypic values (e.g., gene expression) of populations among common gardens (i.e., reaction norm slopes) suggests phenotypic plasticity (Palacio‐López et al., 2015). Future work assessing differential expression across reciprocally transplanted gardens will elucidate the role that phenotypic plasticity plays in the adaptation of crop landraces to changing environments.

Finally, selective pressures other than UV‐B may have contributed to the observed patterns of genetic differentiation. In addition to UV‐B, other abiotic and biotic stressors such as herbivory, rainfall, salinity, and temperature have been shown to induce and/or be buffered by the phenolic compounds discussed in this study (Chalker‐Scott, 1999; Dixon & Paiva, 1995; Falcone Ferreyra, Rius, & Casati, 2012; Kovinich et al., 2014; Nakabayashi et al., 2014). While our findings strongly coincide with previously mentioned examples of UV‐B induced differentiation in maize landraces, additional studies that disentangle these effects of UV‐B irradiance and other selective pressures are needed to determine the causes of observed differential expression patterns. Given our findings, UV‐B supplementation studies, like those described in Casati and Walbot (2005), performed in reciprocal transplant gardens along elevational gradients that score and relate fitness to phenolic compound biosynthesis, are warranted. As are DNA studies looking for genetic differentiation in the promoter regions and transcription factors that regulate the DE phenolic biosynthesis genes described in this study. Combining these approaches may uncover the elusive evolutionary connection between genotype, phenotype, fitness, and natural selective pressures in crop landraces and naturally occurring populations.

### 4.4 Application for agricultural resiliency

To increase agricultural resiliency and sustainability in the face of unprecedented global climate change, it is imperative that we conserve and utilize the diversity contained within the crop landraces and crop wild relatives of the world (Bellon, 2008; Dempewolf et al., 2014, 2017; Mercer et al., 2012; Vigouroux et al., 2011). Many small‐scale farmers planting on two hectares of land or less cultivate, maintain, and facilitate the evolution of diverse crop landraces. In Mexico, maize landrace farmers contribute to both national food security and continue the dynamic process of evolution and in situ conservation of this globally important crop (Bellon et al., 2018). Specific to maize cultivation in Chiapas, Mexico, in a sample of 119 communities (2073 households) throughout four altitudinal environments in the State, between 60% and 100% of small‐scale farmers surveyed planted traditional varieties of maize (Brush & Perales, 2007). Deciphering how genetic diversity in these landraces is shaped by selective pressures and distributed across the landscape can (a) inform targeted in situ conservation strategies and (b) aid in the strategic movement of landrace diversity across the landscape to ensure small‐scale farmers have access to proper germplasm to cope with future abiotic and biotic changes.

Regional transcriptional differentiation studies, as the one reported here set a foundation for DNA‐based studies looking at the spatial distribution of functional genetic diversity in crop landraces across the landscape. Identifying DNA level differences within the promoters of DE genes involved in local adaptation and the transcription factors regulating them is a logical next step toward the execution of landscape level allelic diversity studies aimed at informing in situ conservation and utilization efforts. For instance, allelic diversity information could be employed in the Weitzman approach to diversity conservation where information on rare and endangered diversity is used to determine the most efficient allocation of resources to strengthen in situ conservation efforts (Weitzman, 1992, 1993). In addition, breeders could utilize such information to generate new crop varieties in preparation of continuously changing climates. Gaining understanding of diversity is a first step in its conservation and utilization.

## CONFLICT OF INTEREST

None declared.

## Supporting information

Supplementary Material

## Data Availability

Data available from the Dryad Digital Repository: https://doi.org/10.5061/dryad.gxd2547h1
