# Supplementary material for: Transcriptional differentiation of UV‐B protectant genes in maize landraces spanning an elevational gradient in Chiapas, Mexico
Source: Evol Appl. 2020 Apr 3;13(8):1949–67. doi: 10.1111/eva.12954 (PMC7463351; doi:10.1111/eva.12954)
Supplement: Supplementary file 1 — Supplementary Material [file EVA-13-1949-s001.docx]

**Supporting Information**

Table S1. Phenolic compound biosynthesis gene list

| **GRMZM number** | **General gene name** | **Enzyme description** |
| --- | --- | --- |
| GRMZM2G008539^F^ | *3GT* | Anthocyanidin 3-O-glucosyltransferase,UDPG-flavonoid 3-oxy glucosyl transferase |
| GRMZM2G022266^F^ | *3GT* | Anthocyanidin 3-O-glucosyltransferase,UDPG-flavonoid 3-oxy glucosyl transferase |
| GRMZM2G061289^F^ | *3GT* | Anthocyanidin 3-O-glucosyltransferase,UDPG-flavonoid 3-oxy glucosyl transferase |
| GRMZM2G061321^F^ | *3GT* | Anthocyanidin 3-O-glucosyltransferase,UDPG-flavonoid 3-oxy glucosyl transferase |
| GRMZM2G063550^F^ | *3GT* | Anthocyanidin 3-O-glucosyltransferase,UDPG-flavonoid 3-oxy glucosyl transferase |
| GRMZM2G066067^F^ | *3GT* | Anthocyanidin 3-O-glucosyltransferase,UDPG-flavonoid 3-oxy glucosyl transferase |
| GRMZM2G082249^F^ | *3GT* | Anthocyanidin 3-O-glucosyltransferase,UDPG-flavonoid 3-oxy glucosyl transferase |
| GRMZM2G083130^F^ | *3GT* | Anthocyanidin 3-O-glucosyltransferase,UDPG-flavonoid 3-oxy glucosyl transferase |
| GRMZM2G099740^F^ | *3GT* | Anthocyanidin 3-O-glucosyltransferase,UDPG-flavonoid 3-oxy glucosyl transferase |
| GRMZM2G135722^F^ | *3GT* | Anthocyanidin 3-O-glucosyltransferase,UDPG-flavonoid 3-oxy glucosyl transferase |
| GRMZM2G159404^F^ | *3GT* | Anthocyanidin 3-O-glucosyltransferase,UDPG-flavonoid 3-oxy glucosyl transferase |
| GRMZM2G162755^F^ | *3GT* | Anthocyanidin 3-O-glucosyltransferase,UDPG-flavonoid 3-oxy glucosyl transferase |
| GRMZM2G162783^F^ | *3GT* | Anthocyanidin 3-O-glucosyltransferase,UDPG-flavonoid 3-oxy glucosyl transferase |
| GRMZM2G165390^F^ | *3GT – Bz1* | Anthocyanidin 3-O-glucosyltransferase,UDPG-flavonoid 3-oxy glucosyl transferase |
| GRMZM2G180283^F^ | *3GT* | Anthocyanidin 3-O-glucosyltransferase,UDPG-flavonoid 3-oxy glucosyl transferase |
| GRMZM2G383404^F^ | *3GT* | Anthocyanidin 3-O-glucosyltransferase,UDPG-flavonoid 3-oxy glucosyl transferase |
| GRMZM2G478996^F^ | *3GT* | Anthocyanidin 3-O-glucosyltransferase,UDPG-flavonoid 3-oxy glucosyl transferase |
| GRMZM2G014651^G^* | *4CL* | 4-coumarate--CoA ligase |
| GRMZM2G019746^G^ | *4CL* | 4-coumarate--CoA ligase |
| GRMZM2G048522^G^ | *4CL* | 4-coumarate--CoA ligase |
| GRMZM2G054013^G^ | *4CL* | 4-coumarate--CoA ligase |
| GRMZM2G055320^G^ | *4CL – 4CL1* | 4-coumarate--CoA ligase |
| GRMZM2G075333^G^ | *4CL* | 4-coumarate--CoA ligase |
| GRMZM2G091643^G^ | *4CL* | 4-coumarate--CoA ligase |
| GRMZM2G096020^G^ | *4CL – 4CL3* | 4-coumarate--CoA ligase |
| **GRMZM number** | **General gene name** | **Enzyme description** |
| GRMZM2G122787^G^ | *4CL – 4CL4* | 4-coumarate--CoA ligase |
| GRMZM2G145179^G^* | *4CL* | 4-coumarate--CoA ligase |
| GRMZM2G174574^G^ | *4CL – 4CL2* | 4-coumarate--CoA ligase |
| GRMZM2G174732^G^ | *4CL* | 4-coumarate--CoA ligase |
| GRMZM2G433624^G^ | *4CL* | 4-coumarate--CoA ligase |
| GRMZM5G805585^G^ | *4CL* | 4-coumarate--CoA ligase |
| GRMZM2G058675 | *ALDH – ALDH1 (rf2)* | Coniferyl-aldehyde dehydrogenase |
| GRMZM2G071021 | *ALDH* | Coniferyl-aldehyde dehydrogenase |
| GRMZM2G097706 | *ALDH – ALDH5* | Coniferyl-aldehyde dehydrogenase |
| GRMZM2G125268 | *ALDH – ALDH2* | Coniferyl-aldehyde dehydrogenase |
| GRMZM2G407949 | *ALDH* | Coniferyl-aldehyde dehydrogenase |
| GRMZM2G007053^F^ | *-* | Flavonoid:NAD(P)+ oxidoreductase |
| GRMZM2G097841^F^ | *-* | Flavonoid:NAD(P)+ oxidoreductase |
| GRMZM2G431504^F^ | *-* | Flavonoid:NAD(P)+ oxidoreductase |
| GRMZM2G063949^N^ | *AXS* | Nucleotide-sugar interconversion |
| GRMZM2G085381^F^ | *Bx1* | DIBOA / DIMBOA biosynthesis |
| GRMZM2G085661 | *Bx2* | DIBOA / DIMBOA biosynthesis |
| GRMZM2G167549 | *Bx3* | DIBOA / DIMBOA biosynthesis |
| GRMZM2G085054 | *Bx8* | Benzoxazinoid glucosides biosynthesis |
| GRMZM2G161335 | *Bx9* | Benzoxazinoid glucosides biosynthesis |
| GRMZM2G138074^L^ | *C3H* | p-coumarate 3-hydroxylase |
| GRMZM2G010468^G^ | *C4H* | Cinnamate 4-Hydroxylase |
| GRMZM2G028677^G^ | *C4H* | Cinnamate 4-Hydroxylase |
| GRMZM2G139874^G^ | *C4H – C4H2* | Cinnamate 4-Hydroxylase |
| GRMZM2G147245^G^ | *C4H – C4H1* | Cinnamate 4-Hydroxylase |
| AC234163.1^L^ | *CAD* | Cinnamyl alcohol dehydrogenase |
| GRMZM2G046070^L^ | *CAD – CAD1* | Cinnamyl alcohol dehydrogenase |
| GRMZM2G090980^L^ | *CAD* | Cinnamyl alcohol dehydrogenase |
| **GRMZM number** | **General gene name** | **Enzyme description** |
| GRMZM2G118610^L^ | *CAD* | Cinnamyl alcohol dehydrogenase |
| GRMZM2G167613^L^ | *CAD* | Cinnamyl alcohol dehydrogenase |
| GRMZM2G443445^L^ | *CAD* | Cinnamyl alcohol dehydrogenase |
| GRMZM2G700188^L^ | *CAD* | Cinnamyl alcohol dehydrogenase |
| [GRMZM5G844562](http://www.maizesequence.org/Zea_mays/Transcript/Summary?transcript=GRMZM5G844562_T01)^L^ | *CAD – CAD3 (bm1)* | Cinnamyl alcohol dehydrogenase |
| GRMZM2G004138^L^ | *CCoAOMT* | Caffeoyl-CoA O-methyltransferase |
| GRMZM2G033952^L^ | *CCoAOMT – CCoAOMT1* | Caffeoyl-CoA O-methyltransferase |
| GRMZM2G077486^L^ | *CCoAOMT – OMT3* | Caffeoyl-CoA O-methyltransferase |
| GRMZM2G099363^L^ | *CCoAOMT – CCoAOMT2* | Caffeoyl-CoA O-methyltransferase |
| GRMZM2G127948^L^ | *CCoAOMT* | Caffeoyl-CoA O-methyltransferase |
| GRMZM2G332522^L^ | *CCoAOMT* | Caffeoyl-CoA O-methyltransferase |
| [AC234526.1_FGT005](http://www.maizesequence.org/Zea_mays/Transcript/Summary?db=core;g=AC234526.1_FG005;r=7:133868805-133871107;t=AC234526.1_FGT005)^L^ | *CCR* | [Cinnamoyl-coA reductase (CCR)](http://www.polebio.scsv.ups-tlse.fr/MAIZEWALL/descr.php?id=74) |
| GRMZM2G009681^L^ | *CCR* | [Cinnamoyl-coA reductase (CCR)](http://www.polebio.scsv.ups-tlse.fr/MAIZEWALL/descr.php?id=74) |
| GRMZM2G016836^L^ | *CCR – CCR4* | [Cinnamoyl-coA reductase (CCR)](http://www.polebio.scsv.ups-tlse.fr/MAIZEWALL/descr.php?id=74) |
| GRMZM2G017285^L^ | *CCR* | [Cinnamoyl-coA reductase (CCR)](http://www.polebio.scsv.ups-tlse.fr/MAIZEWALL/descr.php?id=74) |
| GRMZM2G034069^L^ | *CCR* | [Cinnamoyl-coA reductase (CCR)](http://www.polebio.scsv.ups-tlse.fr/MAIZEWALL/descr.php?id=74) |
| GRMZM2G050076^L^ | *CCR* | [Cinnamoyl-coA reductase (CCR)](http://www.polebio.scsv.ups-tlse.fr/MAIZEWALL/descr.php?id=74) |
| [GRMZM2G057328](http://www.maizesequence.org/Zea_mays/Transcript/ProteinSummary?peptide=GRMZM2G057328_P01)^L^ | *CCR* | [Cinnamoyl-coA reductase (CCR)](http://www.polebio.scsv.ups-tlse.fr/MAIZEWALL/descr.php?id=74) |
| GRMZM2G078480^L^ | *CCR* | [Cinnamoyl-coA reductase (CCR)](http://www.polebio.scsv.ups-tlse.fr/MAIZEWALL/descr.php?id=74) |
| GRMZM2G099420^L^ | *CCR – CCR1* | [Cinnamoyl-coA reductase (CCR)](http://www.polebio.scsv.ups-tlse.fr/MAIZEWALL/descr.php?id=74) |
| GRMZM2G107076^L^ | *CCR* | [Cinnamoyl-coA reductase (CCR)](http://www.polebio.scsv.ups-tlse.fr/MAIZEWALL/descr.php?id=74) |
| GRMZM2G109720^L^ | *CCR* | [Cinnamoyl-coA reductase (CCR)](http://www.polebio.scsv.ups-tlse.fr/MAIZEWALL/descr.php?id=74) |
| GRMZM2G110881^L^ | *CCR* | [Cinnamoyl-coA reductase (CCR)](http://www.polebio.scsv.ups-tlse.fr/MAIZEWALL/descr.php?id=74) |
| GRMZM2G131205^L^ | *CCR – CCR3* | [Cinnamoyl-coA reductase (CCR)](http://www.polebio.scsv.ups-tlse.fr/MAIZEWALL/descr.php?id=74) |
| GRMZM2G131836^L^ | *CCR* | [Cinnamoyl-coA reductase (CCR)](http://www.polebio.scsv.ups-tlse.fr/MAIZEWALL/descr.php?id=74) |
| GRMZM2G141350^L^ | *CCR* | [Cinnamoyl-coA reductase (CCR)](http://www.polebio.scsv.ups-tlse.fr/MAIZEWALL/descr.php?id=74) |
| GRMZM2G146031^L^ | *CCR* | [Cinnamoyl-coA reductase (CCR)](http://www.polebio.scsv.ups-tlse.fr/MAIZEWALL/descr.php?id=74) |
| GRMZM2G168893^L^ | *CCR* | [Cinnamoyl-coA reductase (CCR)](http://www.polebio.scsv.ups-tlse.fr/MAIZEWALL/descr.php?id=74) |
| **GRMZM number** | **General gene name** | **Enzyme description** |
| GRMZM2G179981^L^ | *CCR* | [Cinnamoyl-coA reductase (CCR)](http://www.polebio.scsv.ups-tlse.fr/MAIZEWALL/descr.php?id=74) |
| GRMZM2G119186^F^ | *CHI* | Chalcone isomerase |
| GRMZM2G155329^F^ | *CHI – CHI1* | Chalcone isomerase |
| GRMZM2G175076^F^ | *CHI* | Chalcone isomerase |
| GRMZM2G009348^F^ | *CHS* | Chalcone synthase, putative |
| GRMZM2G009510^F^ | *CHS* | Chalcone synthase, putative |
| GRMZM2G151227^F^ | *CHS – WHP1* | Chalcone synthase, putative |
| GRMZM2G175812^F^ | *CHS* | Chalcone synthase, putative |
| GRMZM2G346095^F^ | *CHS* | Chalcone synthase, putative |
| GRMZM2G422750^F^ | *CHS – C2* | Chalcone synthase, putative |
| GRMZM2G435393^F^ | *CHS* | Chalcone synthase, putative |
| AC196475.3^L^ | *COMT – COMT1 (bm3)* | Caffeic acid 3-O-methyltransferase |
| GRMZM2G082007^L^ | *COMT – COMT2* | Caffeic acid 3-O-methyltransferase |
| GRMZM2G423331^L^ | *COMT* | Caffeic acid 3-O-methyltransferase |
| GRMZM2G013726^F^ | *DFR – A4* | Dihydroflavonol-4-reductase |
| GRMZM2G026930^F^ | *DFR – A1* | Dihydroflavonol-4-reductase |
| GRMZM2G097854^F^ | *DFR* | Dihydroflavonol-4-reductase |
| GRMZM2G025870^F^ | *F3H* | Naringenin,2-oxoglutarate 3-dioxygenase |
| GRMZM2G050234^F^ | *F3H – F3H2* | Naringenin,2-oxoglutarate 3-dioxygenase |
| GRMZM2G058024^F^ | *F3H* | Naringenin,2-oxoglutarate 3-dioxygenase |
| GRMZM2G062396^F^ | *F3H – F3H1 (fht1)* | Naringenin,2-oxoglutarate 3-dioxygenase |
| GRMZM2G117246^F^ | *F3H* | Naringenin,2-oxoglutarate 3-dioxygenase |
| GRMZM2G122280^F^ | *F3H* | Naringenin,2-oxoglutarate 3-dioxygenase |
| GRMZM2G025832^F^ | *F3’H – F3'H (pr1)* | Flavonoid 3'-hydroxylase |
| GRMZM2G049424^F^* | *F3'H* | Flavonoid 3'-hydroxylase |
| GRMZM2G160763^F^* | *F3'H – F3'H2* | Flavonoid 3'-hydroxylase |
| GRMZM2G313854^F^* | *F3'H* | Flavonoid 3'-hydroxylase |
| AC210173.4_FGT005^L^ | *F5H – F5H1* | Ferulate-5-hydroxylase |
| **GRMZM number** | **General gene name** | **Enzyme description** |
| GRMZM2G100158^L^ | *F5H* | Ferulate-5-hydroxylase |
| GRMZM2G042865^F^ | *FGT* | Flavonol glucosyltransferase |
| GRMZM2G074631^F^ | *FGT* | Flavonol glucosyltransferase |
| GRMZM2G161625^F^ | *FGT* | Flavonol glucosyltransferase |
| GRMZM2G325023^F^ | *FGT* | Flavonol glucosyltransferase |
| GRMZM2G069298^F^ | *FLS – FLS2* | Flavonol synthase/flavanone 3-hydroxylase |
| GRMZM2G152801^F^ | *FLS – FLS1* | Flavonol synthase/flavanone 3-hydroxylase |
| GRMZM2G099467^F^ | *FNS – FNS1 (FNSI-1)* | Flavone synthase |
| GRMZM2G167336^F^ | *FNS* | Flavone synthase |
| GRMZM2G475380^F^ | *FNS – FNSI-2* | Flavone synthase |
| GRMZM2G017678^N^ | *GAE* | Nucleotide-sugar interconversion |
| GRMZM2G042179^N^ | *GAE* | Nucleotide-sugar interconversion |
| GRMZM2G170336^N^ | *GAE* | Nucleotide-sugar interconversion |
| GRMZM2G029856^N^ | *GER* | Nucleotide-sugar interconversion |
| GRMZM2G115124^N^ | *GMD* | Nucleotide-sugar interconversion |
| GRMZM2G138907^N^ | *GME* | Nucleotide-sugar interconversion |
| GRMZM2G016241^F^ | *GST – Bz2* | Glutathione S-transferase |
| GRMZM2G005046^L^ | *HCT* | Shikimate O-hydroxycinnamoyltransferase |
| GRMZM2G015793^L^ | *HCT* | Shikimate O-hydroxycinnamoyltransferase |
| GRMZM2G034360 ^L^ | *HCT – HCT10* | Shikimate O-hydroxycinnamoyltransferase |
| GRMZM2G035023^L^ | *HCT* | Shikimate O-hydroxycinnamoyltransferase |
| GRMZM2G035584^L^ | *HCT – HCT6* | Shikimate O-hydroxycinnamoyltransferase |
| GRMZM2G037591^L^* | *HCT* | Shikimate O-hydroxycinnamoyltransferase |
| GRMZM2G043154^L^ | *HCT* | Shikimate O-hydroxycinnamoyltransferase |
| GRMZM2G050450^L^ | *HCT* | Shikimate O-hydroxycinnamoyltransferase |
| GRMZM2G051005^L^ | *HCT* | Shikimate O-hydroxycinnamoyltransferase |
| GRMZM2G063139^L^ | *HCT* | Shikimate O-hydroxycinnamoyltransferase |
| GRMZM2G064969^L^ | *HCT* | Shikimate O-hydroxycinnamoyltransferase |
| **GRMZM number** | **General enzyme name** | **Enzyme description** |
| GRMZM2G070468^L^ | *HCT* | Shikimate O-hydroxycinnamoyltransferase |
| GRMZM2G094017^L^ | *HCT* | Shikimate O-hydroxycinnamoyltransferase |
| GRMZM2G107211^L^* | *HCT* | Shikimate O-hydroxycinnamoyltransferase |
| GRMZM2G107851^L^ | *HCT* | Shikimate O-hydroxycinnamoyltransferase |
| GRMZM2G115422^L^ | *HCT* | Shikimate O-hydroxycinnamoyltransferase |
| GRMZM2G122503^L^ | *HCT* | Shikimate O-hydroxycinnamoyltransferase |
| GRMZM2G124066^L^ | *HCT* | Shikimate O-hydroxycinnamoyltransferase |
| GRMZM2G124815^L^ | *HCT* | Shikimate O-hydroxycinnamoyltransferase |
| GRMZM2G127251^L^ | *HCT* | Shikimate O-hydroxycinnamoyltransferase |
| GRMZM2G129266^L^ | *HCT – HCT13* | Shikimate O-hydroxycinnamoyltransferase |
| GRMZM2G131165^L^ | *HCT* | Shikimate O-hydroxycinnamoyltransferase |
| GRMZM2G132678^L^ | *HCT* | Shikimate O-hydroxycinnamoyltransferase |
| GRMZM2G147908^L^ | *HCT* | Shikimate O-hydroxycinnamoyltransferase |
| GRMZM2G151553^L^ | *HCT* | Shikimate O-hydroxycinnamoyltransferase |
| GRMZM2G154216^L^* | *HCT* | Shikimate O-hydroxycinnamoyltransferase |
| GRMZM2G156004^L^ | *HCT* | Shikimate O-hydroxycinnamoyltransferase |
| GRMZM2G156296^L^ | *HCT – HCT11* | Shikimate O-hydroxycinnamoyltransferase |
| GRMZM2G156816^L^ | *HCT* | Shikimate O-hydroxycinnamoyltransferase |
| GRMZM2G158083^L^ | *HCT – HCT5* | Shikimate O-hydroxycinnamoyltransferase |
| GRMZM2G165192^L^ | *HCT* | Shikimate O-hydroxycinnamoyltransferase |
| GRMZM2G168499^L^* | *HCT* | Shikimate O-hydroxycinnamoyltransferase |
| GRMZM2G176446^L^ | *HCT* | Shikimate O-hydroxycinnamoyltransferase |
| GRMZM2G177349^L^ | *HCT* | Shikimate O-hydroxycinnamoyltransferase |
| GRMZM2G178769^L^ | *HCT* | Shikimate O-hydroxycinnamoyltransferase |
| GRMZM2G179703^L^ | *HCT – HCT12* | Shikimate O-hydroxycinnamoyltransferase |
| GRMZM2G417382^L^* | *HCT* | Shikimate O-hydroxycinnamoyltransferase |
| GRMZM2G446652^L^* | *HCT* | Shikimate O-hydroxycinnamoyltransferase |
| GRMZM2G000423^F^ | *LDOX* | Leucoanthocyanidin dioxygenase |
| **GRMZM number** | **General enzyme name** | **Enzyme description** |
| GRMZM2G162158^F^ | *LDOX* | Leucoanthocyanidin dioxygenase |
| GRMZM2G345717^F^ | *LDOX – A2* | Leucoanthocyanidin dioxygenase |
| GRMZM2G426175^F^ | *LDOX* | Leucoanthocyanidin dioxygenase |
| GRMZM2G029048^G^ | *PAL – PAL9* | Phenylalanine ammonia-lyase |
| GRMZM2G063917^G^ | *PAL – PAL4* | Phenylalanine ammonia-lyase |
| GRMZM2G074604^G^ | *PAL – PAL1* | Phenylalanine ammonia-lyase |
| GRMZM2G081582^G^ | *PAL – PAL5* | Phenylalanine ammonia-lyase |
| GRMZM2G118345^G^ | *PAL – PAL6* | Phenylalanine ammonia-lyase |
| GRMZM2G153871^G^ | *PAL* | Phenylalanine ammonia-lyase |
| GRMZM2G160541^G^ | *PAL – PAL3* | Phenylalanine ammonia-lyase |
| GRMZM2G170692^G^ | *PAL – PAL7* | Phenylalanine ammonia-lyase |
| GRMZM2G334660^G^ | *PAL – PAL8* | Phenylalanine ammonia-lyase |
| GRMZM2G441347^G^ | *PAL – PAL2* | Phenylalanine ammonia-lyase |
| GRMZM2G129761 | *PER* | 1-Cys peroxiredoxin |
| GRMZM2G031311^F^ | *RHM – RHS1 (SM1)* | Nucleotide-sugar interconversion |
| GRMZM2G044281^N^ | *RHM – RHM3* | Nucleotide-sugar interconversion |
| GRMZM2G072911^N^ | *RHM – RHM2* | Nucleotide-sugar interconversion |
| GRMZM2G166767^N^ | *RHM – RHM4* | Nucleotide-sugar interconversion |
| GRMZM2G311117^N^ | *RHM* | Nucleotide-sugar interconversion |
| GRMZM2G328500 ^N^ | *UGD* | Nucleotide-sugar interconversion |
| GRMZM2G409642^N^ | *UGD* | Nucleotide-sugar interconversion |
| GRMZM2G007404^N^ | *UXS* | Nucleotide-sugar interconversion |
| GRMZM2G044027^N^ | *UXS* | Nucleotide-sugar interconversion |
| GRMZM2G165357^N^ | *UXS* | Nucleotide-sugar interconversion |
| GRMZM2G359234^N^ | *UXS* | Nucleotide-sugar interconversion |

Note. A comprehensive list of genes involved in maize phenolic compound biosynthesis. Genes reported in [1, 2, 3], at http://cellwall.genomics.purdue.edu, genes obtained by NCBI BLAST searches, recently identified genes (E. Grotewold, pers. comm.), and genes identified during a MaizeCyc analysis were compiled to generate this list of 190 genes. The maize gene ID (GRMZM number), enzyme gene abbreviation (General gene name), and a description of the enzyme (Enzyme description) are provided for each gene. The initial text in the ‘General gene name’ is the generic name of the enzyme while text following the en dash (–) is the naming scheme presented in [4]. When appropriate, we used specific gene names. Genes that were removed to generate the list of 179 genes used in the hierarchical clustering analysis are represented with an *. The sub-portions of the list used in the enrichment analyses are abbreviated as follows: ^L^ Lignin Biosynthesis; ^G^ General Phenylpropanoid Biosynthesis; ^F^ Flavonoid Biosynthesis; and ^N^ Nucleotide-sugar interconversion.

1. Penning, B. W., Hunter, C. T., Tayengwa, R., Eveland, A. L., Dugard, C. K., Olek, A. T., ... & Thomas, S. R. (2009). Genetic resources for maize cell wall biology. *Plant Physiology*, *151*(4), 1703-1728.

2. Fornalé, S., Shi, X., Chai, C., Encina, A., Irar, S., Capellades, M., ... & Rigau, J. (2010). ZmMYB31 directly represses maize lignin genes and redirects the phenylpropanoid metabolic flux. *The Plant Journal*, *64*(4), 633-644.

3. Sekhon, R. S., Lin, H., Childs, K. L., Hansey, C. N., Buell, C. R., de Leon, N., & Kaeppler, S. M. (2011). Genome-wide atlas of transcription during maize development. *The Plant Journal*, *66*(4), 553-563..

4. Yang, F., Li, W., Jiang, N., Yu, H., Morohashi, K., Ouma, W. Z., ... & Velazquez, R. A. (2017). A Maize Gene Regulatory Network for Phenolic Metabolism. *Molecular plant*, *10*(3), 498-515.

Table S2. Raw, trimmed, and mapped read counts for each of the 45 maize landrace RNA-seq libraries

| Elevation | ID | Raw counts | Trimmed paired-end | Trimmed singletons | Uniquely mapped reads (0 mismatches) | | Uniquely mapped reads  (2 mismatches) |
| --- | --- | --- | --- | --- | --- | --- | --- |
| Lowland | 1.1 | 10,439,682 | 10,182,584 | 239,606 | 7,139,399 | 8,057,106 | |
|  | 1.2 | 10,430,532 | 9,782,834 | 569,155 | 6,740,973 | 7,794,724 | |
|  | 1.3 | 10,504,192 | 9,945,954 | 480,085 | 7,086,370 | 8,157,758 | |
|  | 4.1 | 11,292,632 | 11,011,414 | 261,899 | 7,831,552 | 8,874,598 | |
|  | 4.2 | 12,825,010 | 12,097,683 | 679,222 | 8,429,932 | 9,649,951 | |
|  | 4.3 | 10,330,470 | 9,765,568 | 486,097 | 7,115,540 | 8,142,660 | |
|  | 6.1 | 13,581,764 | 13,032,203 | 446,709 | 9,150,585 | 10,457,264 | |
|  | 6.2 | 20,737,732 | 19,587,880 | 1,038,222 | 11,991,434 | 13,825,068 | |
|  | 6.3 | 9,568,905 | 9,035,405 | 453,474 | 6,617,719 | 7,631,794 | |
|  | 7.1 | 11,153,956 | 10,881,011 | 252,581 | 7,986,770 | 9,019,609 | |
|  | 7.2 | 13,818,036 | 13,013,686 | 732,726 | 8,709,307 | 10,073,804 | |
|  | 7.3 | 9,462,968 | 8,884,115 | 474,976 | 6,410,195 | 7,411,220 | |
|  | 9.1 | 10,380,385 | 10,112,423 | 249,198 | 7,186,884 | 8,143,160 | |
|  | 9.2 | 13,513,444 | 12,755,328 | 691,518 | 9,311,219 | 10,726,555 | |
|  | 9.3 | 10,195,298 | 9,625,875 | 493,493 | 6,884,325 | 7,914,780 | |
| Midland | 10.1 | 10,588,864 | 10,250,995 | 304,751 | 7,495,939 | 8,521,976 | |
|  | 10.2 | 7,279,128 | 6,881,896 | 355,851 | 4,898,764 | 5,644,246 | |
|  | 10.3 | 9,845,739 | 9,276,558 | 499,067 | 6,334,315 | 7,348,480 | |
|  | 12.1 | 13,214,762 | 12,818,955 | 359,711 | 9,138,876 | 10,408,581 | |
|  | 12.2 | 9,991,418 | 9,674,662 | 277,799 | 6,815,411 | 7,763,058 | |
|  | 12.3 | 11,947,573 | 11,385,725 | 509,951 | 7,716,082 | 8,838,362 | |
|  | 13.1 | 13,083,726 | 12,648,770 | 394,578 | 9,064,717 | 10,346,368 | |
|  | 13.2 | 11,762,135 | 10,954,138 | 654,484 | 7,849,029 | 9,144,867 | |
|  | 13.3 | 11,967,217 | 11,346,672 | 573,631 | 7,729,959 | 8,962,273 | |
|  | 17.1 | 13,528,138 | 13,129,632 | 359,839 | 8,996,865 | 10,214,845 | |
|  | 17.2 | 11,949,060 | 11,568,709 | 332,091 | 8,218,826 | 9,367,518 | |
| Table S2. (cont.) Raw, trimmed, and mapped read counts for each of the 45 maize landrace RNA-seq libraries | | | | | | | |
| Elevation | ID | Raw counts | Trimmed paired-end | Trimmed singletons | Uniquely mapped reads (0 mismatches) | Uniquely mapped reads  (2 mismatches) | |
| Midland | 17.3 | 16,961,938 | 16,093,723 | 792,990 | 10,963,688 | 12,694,676 | |
|  | 18.1 | 13,683,294 | 13,236,512 | 387,945 | 9,607,943 | 10,948,525 | |
|  | 18.2 | 12,277,769 | 11,887,437 | 344,576 | 8,388,803 | 9,577,505 | |
|  | 18.3 | 5,292,851 | 5,015,296 | 250,365 | 3,581,160 | 4,125,075 | |
| Highland | 20.1 | 12,883,767 | 12,390,750 | 444,418 | 8,712,643 | 10,008,828 | |
|  | 20­­.2 | 12,242,627 | 11,886,034 | 321,072 | 8,251,586 | 9,432,015 | |
|  | 20­.3 | 14,205,156 | 13,461,901 | 637,821 | 9,566,940 | 11,007,865 | |
|  | 26.1 | 14,919,832 | 14,418,754 | 457,460 | 10,106,081 | 11,570,013 | |
|  | 26.2 | 12,723,433 | 12,339,342 | 340,536 | 8,289,458 | 9,455,402 | |
|  | 26.3 | 11,444,355 | 10,840,889 | 527,836 | 7,547,418 | 8,728,387 | |
|  | 27.1 | 13,297,370 | 12,839,493 | 417,909 | 9,189,327 | 10,546,302 | |
|  | 27.2 | 12,279,665 | 11,859,702 | 358,126 | 8,133,263 | 9,321,028 | |
|  | 27.3 | 12,208,517 | 11,622,856 | 537,065 | 8,028,978 | 9,294,031 | |
|  | 29.1 | 10,501,789 | 10,231,288 | 251,833 | 6,667,214 | 7,551,604 | |
|  | 29.2 | 13,590,231 | 13,130,261 | 407,067 | 9,584,572 | 10,961,216 | |
|  | 29.3 | 11,365,436 | 10,815,481 | 499,461 | 7,712,568 | 8,918,749 | |
|  | 30.1 | 14,952,693 | 14,396,962 | 501,474 | 10,230,359 | 11,774,007 | |
|  | 30.2 | 11,267,823 | 10,902,335 | 326,390 | 7,755,414 | 8,867,647 | |
|  | 30.3 | 12,536,177 | 11,944,892 | 544,556 | 7,699,196 | 8,864,515 | |

Legend. Read counts for each library before (raw counts) and after read preprocessing (trimmed paired end and trimmed singletons) and after mapping reads to the maize genome (uniquely mapped reads – 0 mismatches & 2 mismatches). Uniquely mapped reads were recorded when allowing both 0 & 2 mismatches. The ID column includes three replicates for each population (i.e., 1.1, 1.2, 1.3 replicates 1-3 for population one).

Table S3a. Highland–lowland maize landrace comparison: log2Fold change values of significantly differentially expressed genes involved in phenylpropanoid, flavonoid, and lignin biosynthesis, and nucleotide-sugar interconversion

| **Phenylpropanoid and flavonoid biosynthesis** | | | | | | | |
| --- | --- | --- | --- | --- | --- | --- | --- |
| **Gene** | **Maize gene ID** | **baseMean** | **log2FoldChange** | **lfcSE** | **stat** | **pvalue** | **padj** |
| *C4H* | GRMZM2G028677 | 15.99 | 1.60 | 0.31 | 5.19 | 2.05E-07 | 2.45E-05 |
| *4CL* | GRMZM2G091643 | 7.47 | -2.07 | 0.42 | -4.90 | 9.43E-07 | 8.12E-05 |
| *4CL2* | GRMZM2G174574 | 225.98 | -0.54 | 0.13 | -4.06 | 4.87E-05 | 0.002 |
| *4CL* | GRMZM5G805585 | 146.57 | -0.70 | 0.20 | -3.44 | <0.001 | 0.011 |
| *CHS* | GRMZM2G435393 | 1.49 | -1.56 | 0.43 | -3.60 | <0.001 | 0.007 |
| *CHI1* | GRMZM2G155329 | 357.37 | -0.35 | 0.13 | -2.81 | 0.0050 | 0.049 |
| *F3H* | GRMZM2G025870 | 33.28 | 0.57 | 0.17 | 3.31 | <0.001 | 0.015 |
| *FNS1 (FNSI-1)* | GRMZM2G099467 | 312.27 | -0.68 | 0.19 | -3.61 | <0.001 | 0.007 |
| *FGT* | GRMZM2G042865 | 8.57 | 1.51 | 0.28 | 5.46 | 4.81E-08 | 7.09E-06 |
| *FGT* | GRMZM2G074631 | 57.91 | 2.10 | 0.21 | 10.18 | 2.32E-24 | 6.90E-21 |
| *FGT* | GRMZM2G161625 | 4.89 | 1.37 | 0.38 | 3.64 | <0.001 | 0.006 |
| *FGT* | GRMZM2G325023 | 3.07 | -1.23 | 0.36 | -3.45 | 0.0010 | 0.011 |
| *3GT* | GRMZM2G022266 | 107.74 | -0.49 | 0.15 | -3.36 | <0.001 | 0.014 |
| *3GT* | GRMZM2G063550 | 3.44 | -2.04 | 0.41 | -5.02 | 5.06E-07 | 5.07E-05 |
| *3GT* | GRMZM2G383404 | 18.94 | -0.61 | 0.18 | -3.40 | <0.001 | 0.012 |
|  |  |  |  |  |  |  |  |
| **Lignin biosynthesis** | |  |  |  |  |  |  |
| **Gene** | **Maize gene ID** | **baseMean** | **log2FoldChange** | **lfcSE** | **stat** | **pvalue** | **padj** |
| *HCT10* | GRMZM2G034360 | 18.28 | 0.77 | 0.19 | 4.03 | 5.51E-05 | 0.002 |
| *HCT6* | GRMZM2G035584 | 137.03 | -0.34 | 0.11 | -3.13 | 0.0017 | 0.024 |
| *HCT* | GRMZM2G122503 | 11.42 | -2.11 | 0.36 | -5.82 | 5.92E-09 | 1.15E-06 |
| *HCT* | GRMZM2G124066 | 18.49 | -0.70 | 0.18 | -3.92 | 8.93E-05 | 0.003 |
| *HCT13* | GRMZM2G129266 | 85.15 | -0.62 | 0.18 | -3.43 | <0.001 | 0.011 |

Table S3a. (cont.) Highland–lowland maize landrace comparison: log2Fold change values of significantly differentially expressed genes involved in phenylpropanoid, flavonoid, and lignin biosynthesis, and nucleotide-sugar interconversion

| **Lignin biosynthesis** | |  |  |  |  |  |  |
| --- | --- | --- | --- | --- | --- | --- | --- |
| **Gene** | **Maize gene ID** | **baseMean** | **log2FoldChange** | **lfcSE** | **stat** | **pvalue** | **padj** |
| *HCT11* | GRMZM2G156296 | 63.78 | -0.76 | 0.22 | -3.51 | <0.001 | 0.009 |
| *HCT12* | GRMZM2G179703 | 74.58 | 0.66 | 0.20 | 3.37 | <0.001 | 0.013 |
| *CCR* | GRMZM2G009681 | 146.75 | -0.71 | 0.23 | -3.15 | 0.0016 | 0.023 |
| *CCR* | GRMZM2G168893 | 12.74 | 0.55 | 0.17 | 3.21 | 0.0013 | 0.020 |
| *OMT3* | GRMZM2G077486 | 148.16 | -0.66 | 0.11 | -5.76 | 8.19E-09 | 1.50E-06 |
| *ALDH2* | GRMZM2G125268 | 20.13 | -0.92 | 0.22 | -4.08 | 4.44E-05 | 0.002 |
| *CAD* | GRMZM2G118610 | 1029.71 | -0.50 | 0.18 | -2.87 | 0.0041 | 0.043 |
|  |  |  |  |  |  |  |  |
| **Plant nucleotide-sugar interconversion** | | |  |  |  |  |  |
| **Gene** | **Maize gene ID** | **baseMean** | **log2FoldChange** | **lfcSE** | **stat** | **pvalue** | **padj** |
| *RHS1 (SM1)* | GRMZM2G031311 | 503.06 | -0.52 | 0.16 | -3.36 | <0.001 | 0.014 |
| *UXS* | GRMZM2G044027 | 259.96 | -0.35 | 0.11 | -3.04 | 0.0023 | 0.030 |

Table S3b. Highland–midland maize landrace comparison: log2Fold change values of significantly differentially expressed genes involved in phenylpropanoid, flavonoid, and lignin biosynthesis, and nucleotide-sugar interconversion

| **Phenylpropanoid and flavonoid biosynthesis** | | | | | | | |
| --- | --- | --- | --- | --- | --- | --- | --- |
| **Gene** | **Maize gene ID** | **baseMean** | **log2FoldChange** | **lfcSE** | **stat** | **pvalue** | **padj** |
| *4CL1* | GRMZM2G055320 | 280.87 | -0.64 | 0.21 | -3.12 | 0.0018 | 0.0261 |
| *F3H2* | GRMZM2G050234 | 20.62 | -1.19 | 0.32 | -3.73 | <0.001 | 0.0061 |
| *FNS1 (FNSI-1)* | GRMZM2G099467 | 312.27 | -0.74 | 0.20 | -3.78 | <0.001 | 0.0053 |
| *FLS2* | GRMZM2G069298 | 8.04 | 1.82 | 0.49 | 3.68 | <0.001 | 0.0070 |
| *FGT* | GRMZM2G325023 | 3.07 | -1.47 | 0.39 | -3.79 | <0.001 | 0.0053 |
| *-* | GRMZM2G007053 | 36.36 | -0.90 | 0.26 | -3.44 | <0.001 | 0.0130 |
| *LDOX* | GRMZM2G162158 | 31.69 | -0.75 | 0.24 | -3.07 | 0.0021 | 0.0287 |
| *3GT* | GRMZM2G135722 | 170.94 | 1.64 | 0.51 | 3.24 | 0.0012 | 0.0204 |
|  |  |  |  |  |  |  |  |
| **Lignin biosynthesis** | |  |  |  |  |  |  |
| **Gene** | **Maize gene ID** | **baseMean** | **log2FoldChange** | **lfcSE** | **stat** | **pvalue** | **padj** |
| *HCT10* | GRMZM2G034360 | 18.28 | 0.60 | 0.20 | 3.09 | 0.0020 | 0.0278 |
| *HCT11* | GRMZM2G156296 | 63.78 | -0.74 | 0.23 | -3.26 | 0.0011 | 0.0194 |
| *CCR3* | GRMZM2G131205 | 1256.62 | 0.51 | 0.18 | 2.92 | 0.0035 | 0.0396 |
| *CCR* | GRMZM2G168893 | 12.74 | 0.74 | 0.18 | 4.17 | 3.06E-05 | 0.0018 |
| *ALDH5* | GRMZM2G097706 | 219.15 | -0.67 | 0.23 | -2.90 | 0.0038 | 0.0414 |
| *ALDH2* | GRMZM2G125268 | 20.13 | -0.82 | 0.23 | -3.51 | <0.001 | 0.0108 |
| *COMT2* | GRMZM2G082007 | 713.19 | 0.27 | 0.08 | 3.32 | <0.001 | 0.0170 |
|  |  |  |  |  |  |  |  |
| **Plant nucleotide-sugar interconversion** | | | | |  |  |  |
| **Gene** | **Maize gene ID** | **baseMean** | **log2FoldChange** | **lfcSE** | **stat** | **pvalue** | **padj** |
| *RHM3* | GRMZM2G044281 | 135.73 | -0.75 | 0.14 | -5.38 | 7.25E-08 | 2.69E-05 |
| *RHM2* | GRMZM2G072911 | 177.74 | -0.21 | 0.07 | -2.87 | 0.0042 | 0.0443 |

Table S3b. (cont.) Highland–midland maize landrace comparison: log2Fold change values of significantly differentially expressed genes involved in phenylpropanoid, flavonoid, and lignin biosynthesis, and nucleotide-sugar interconversion

| **Plant nucleotide-sugar interconversion** | | | | |  |  |  |
| --- | --- | --- | --- | --- | --- | --- | --- |
| **Gene** | **Maize gene ID** | **baseMean** | **log2FoldChange** | **lfcSE** | **stat** | **pvalue** | **padj** |
| *UGD* | GRMZM2G328500 | 545.61 | -0.67 | 0.13 | -5.26 | 1.47E-07 | 4.08E-05 |

Table S3c. Midland–lowland maize landrace comparison: log2Fold change values of significantly differentially expressed genes involved in phenylpropanoid, flavonoid, and lignin biosynthesis, and nucleotide-sugar interconversion

| **Phenylpropanoid and flavonoid biosynthesis** | | | | |  |  |  |
| --- | --- | --- | --- | --- | --- | --- | --- |
| **Gene** | **Maize gene ID** | **baseMean** | **log2FoldChange** | **lfcSE** | **stat** | **pvalue** | **padj** |
| *Bx8* | GRMZM2G085054 | 787.83 | 0.36 | 0.13 | 2.87 | 0.0041 | 0.0378 |
| *C4H* | GRMZM2G028677 | 15.99 | 0.85 | 0.31 | 2.73 | 0.0064 | 0.0495 |
| *4CL* | GRMZM5G805585 | 146.57 | -0.62 | 0.20 | -3.04 | 0.0024 | 0.0262 |
| *FNSI-2* | GRMZM2G475380 | 5.77 | 0.94 | 0.31 | 3.01 | 0.0026 | 0.0278 |
| *FLS2* | GRMZM2G069298 | 8.04 | -1.25 | 0.41 | -3.04 | 0.0024 | 0.0259 |
| *FGT* | GRMZM2G042865 | 8.57 | 1.45 | 0.28 | 5.25 | 1.54E-07 | 2.45E-05 |
| *FGT* | GRMZM2G074631 | 57.91 | 1.58 | 0.21 | 7.60 | 2.88E-14 | 3.97E-11 |
| *3GT* | GRMZM2G063550 | 3.44 | -1.67 | 0.39 | -4.25 | 2.12E-05 | 0.0010 |
| *3GT* | GRMZM2G159404 | 9.73 | -1.01 | 0.27 | -3.76 | <0.001 | 0.0043 |
|  |  |  |  |  |  |  |  |
| **Lignin biosynthesis** | |  |  |  |  |  |  |
| **Gene** | **Maize gene ID** | **baseMean** | **log2FoldChange** | **lfcSE** | **stat** | **pvalue** | **padj** |
| *HCT* | GRMZM2G043154 | 24.51 | -0.85 | 0.30 | -2.86 | 0.0042 | 0.0384 |
| *HCT* | GRMZM2G122503 | 11.42 | -0.99 | 0.35 | -2.83 | 0.0047 | 0.0406 |
| *HCT* | GRMZM2G177349 | 4.03 | -1.38 | 0.45 | -3.09 | 0.0020 | 0.0235 |
| *HCT12* | GRMZM2G179703 | 74.58 | 0.58 | 0.20 | 2.92 | 0.0035 | 0.0334 |
| *CCR1* | GRMZM2G099420 | 10.58 | -1.35 | 0.37 | -3.60 | 0.0003 | 0.0067 |
| *CCR* | GRMZM2G109720 | 230.00 | -0.21 | 0.07 | -2.85 | 0.0043 | 0.0388 |
| *CAD* | GRMZM2G090980 | 688.13 | -0.27 | 0.09 | -3.03 | 0.0025 | 0.0267 |
| *OMT3* | GRMZM2G077486 | 148.16 | -0.34 | 0.11 | -3.00 | 0.0027 | 0.0285 |
|  |  |  |  |  |  |  |  |
|  |  |  |  |  |  |  |  |

Table S3c. (cont.) Midland–lowland maize landrace comparison: log2Fold change values of significantly differentially expressed genes involved in phenylpropanoid, flavonoid, and lignin biosynthesis, and nucleotide-sugar interconversion

| **Plant nucleotide-sugar interconversion enzymes** | | | | |  |  |  |
| --- | --- | --- | --- | --- | --- | --- | --- |
| **Gene** | **Maize gene ID** | **BaseMean** | **log2FoldChange** | **lfcSE** | **stat** | **pvalue** | **padj** |
| *RHM3* | GRMZM2G044281 | 135.73 | 0.41 | 0.14 | 3.03 | 0.0025 | 0.0268 |

Note. Differential expression log2fold change values for phenylpropanoid, flavonoid, and lignin biosynthesis, and plant nucleotide-sugar interconversion enzyme genes for each pairwise comparison of elevation of population origin (highland, midland, and lowland). Positive log2fold change values indicate increased expression for first elevation in comparison: for highland vs. lowland comparison = increased expression in highland populations; highland vs. midland comparison = increased expression in highland populations; midland vs. lowland comparison = increased expression in midland populations. Enzyme gene names (Gene) and their corresponding ID’s (Maize gene ID’s) are described in Table S1. BaseMean, base mean over all samples for a given gene; log2FoldChange, expression difference among comparison; lfcSE, standard error of log2FoldChange; stat, Wald statistic; pvalue, Wald test p-value; padj, Benjamini-Hochberg adjusted p-value for multiple comparisons.

Table S4. Distributions of phenolic candidate genes and leaf background gene lists before and after normalization.

| **Bin #** | **Bin Lower Range** | **Bin Upper Range** | **Candidate Genes** | **Leaf Background Before** | **Leaf Background After** |
| --- | --- | --- | --- | --- | --- |
| 1 | 0.01919 | 760.01185 | 156 | 25616/25431/25512 | 10390 |
| 2 | 760.01185 | 1520. 00451 | 15 | 999/999/999 | 999 |
| 3 | 1520. 00451 | 2279.9972 | 3 | 309/309/309 | 200 |
| 4 | 2279.9972 | 3039.9898 | 1 | 149/148/148 | 67 |
| 5 | 3039.9898 | 3799.9825 | 2 | 87/87/87 | 133 |
| 6 | 3799.9825 | 4559.9751 | 0 | 48/48/48 | 0 |
| 7 | 4559.9751 | 5319.9678 | 1 | 38/38/38 | 67 |
| 8 | 5319.9678 | 6079.9605 | 0 | 25/25/25 | 0 |
| 9 | 6079.9605 | 6839.9531 | 0 | 24/24/24 | 0 |
| 10 | 6839.9531 | 7599.9458 | 1 | 21/21/21 | 67 |

Note. Bin ranges and number of genes per bin for our phenolic candidate gene list and leaf background gene lists. Bin # – Ten equally sized bins based on average counts per gene across all samples. Bin Lower Range and Bin Upper Range – upper and lower bounds of each bin. Candidate Genes – number of candidate genes per bin. Leaf Background Before – the number of leaf expressed background genes per bin before normalizing to the distribution of the candidate genes (highland–lowland/highland–midland/midland–lowland). Leaf Background After – the number of leaf expressed background genes per bin after normalizing to the distribution of the candidate genes. The number of genes per bin was the same for all pairwise comparisons. The Candidate Genes and Leaf Background After columns were used for subsequent enrichment analyses. Bins with red text were not used in the analyses due to few genes being present in them and we imposed a cutoff of 3000 counts. Bins 1-4 contained 98% of the phenolic candidate gene list.

Details of gene Leaf Background Before calculations. Initially, we identified 30,747 genes as being expressed during the V1 to V5 leaf stages in the gene atlas presented in Hoopes et al., (2019). As 3,621 genes were new to the APGv4 naming system, we reduced this number to 27,126 when we translated from the APGv4 to APGv3 gene naming systems. Numerous gene names in AGPv4 represented multiple gene names in AGPv3, increasing the count to 28,807. We removed 199 genes from the list as they were present in the leaf background but not in our DESeq2 normalized file. We then removed an additional 164 genes that we identified as being duplicates which left us with 28,444 genes. Next, we added genes that were DE but that were not recorded as expressed in the leaf tissue of the maize gene atlas; 423 to the highland–lowland comparison, 237 to the highland–midland comparison, and 319 to the midland–lowland comparison.

Table S5. Comparison of the distributions of phenolic candidate genes and leaf background genes.

| **Chi-square statistic** | ***P*-value** | **Different** |
| --- | --- | --- |
| *A. Candidate Genes – Leaf Background Before (156, 15, 3, 1 – 25431, 999, 309, 148)* | | |
| 11.969 | 0.00749 | Yes |
| *B. Candidate Genes – Leaf Background After (156, 15, 3, 1 – 10390, 999, 200, 67)* | | |
| 3.725e-05 | 1 | No |
| *C. Leaf Background Before – Leaf Background After (25431, 999, 309, 148 – 10390, 999, 200, 67)* | | |
| 415.55 | <0.00001 | Yes |

Note. Chi-square test comparing the distributions of the candidate gene list and leaf background before and after adjustment. This analysis was conducted to ensure that the distributions of the leaf expressed background gene set had the same distribution as that of the candidate gene set. Chi-square statistics – values when conducted in R; *P*-value – Yates corrected *p*-value for each comparison; Different – were the two distributions being compared different from each other (Yes) or the same (No).

Table S6a. Population level normalized counts for highland vs. lowland DE genes involved in phenylpropanoid, flavonoid, and lignin biosynthesis, and nucleotide-sugar interconversion

| **Phenylpropanoid and flavonoid biosynthesis** | | | | | | | | | | | | | | | | | |
| --- | --- | --- | --- | --- | --- | --- | --- | --- | --- | --- | --- | --- | --- | --- | --- | --- | --- |
| **Gene** | | **Maize gene ID** | **1.L** | **4.L** | **6.L** | **7.L** | **9.L** | **10.M** | **12.M** | **13.M** | **17.M** | **18.M** | **20.H** | **26.H** | **27.H** | **29.H** | **30.H** |
| *C4H* | | GRMZM2G028677 | 5.6 | 6.1 | 12.4 | 4.3 | 5.7 | 16.5 | 17.0 | 16.1 | 13.2 | 8.6 | **36.3** | **20.0** | **20.2** | **18.3** | **39.8** |
| *4CL* | | GRMZM2G091643 | **1.9** | **41.6** | **12.6** | **18.0** | **5.2** | 3.5 | 7.0 | 2.1 | 2.0 | 9.2 | 2.8 | 1.6 | 1.3 | 2.0 | 1.3 |
| *4CL2* | | GRMZM2G174574 | **299.0** | **234.3** | **257.0** | **277.1** | **263.2** | 270.8 | 257.6 | 241.3 | 219.7 | 161.5 | 226.5 | 134.7 | 148.7 | 185.8 | 212.5 |
| *4CL* | | GRMZM5G805585 | **165.9** | **188.6** | **228.2** | **132.6** | **267.3** | 124.8 | 143.1 | 96.6 | 103.6 | 157.4 | 127.9 | 114.0 | 145.8 | 89.8 | 112.7 |
| *CHS* | | GRMZM2G435393 | **5.4** | **3.1** | **2.6** | **1.4** | **2.0** | 1.1 | 0.3 | 0.7 | 1.0 | 2.2 | 0.3 | 0.9 | 0.6 | 0.6 | 0.3 |
| *CHI1* | | GRMZM2G155329 | **357.0** | **408.8** | **460.6** | **400.9** | **384.5** | 344.2 | 344.7 | 321.7 | 380.4 | 385.1 | 303.4 | 317.6 | 361.3 | 282.6 | 307.8 |
| *F3H* | | GRMZM2G025870 | 25.6 | 34.5 | 20.2 | 24.8 | 32.4 | 42.3 | 28.0 | 30.5 | 23.0 | 25.8 | **39.9** | **50.7** | **48.5** | **35.6** | **37.4** |
| *FNS (FNSI-1)* | | GRMZM2G099467 | **464.6** | **257.5** | **371.1** | **339.1** | **336.7** | 312.9 | 425.4 | 336.8 | 291.6 | 363.6 | 253.2 | 322.7 | 240.1 | 186.0 | 182.5 |
| *FGT* | | GRMZM2G042865 | 2.2 | 4.0 | 2.6 | 4.7 | 2.7 | 18.9 | 7.0 | 14.0 | 8.0 | 7.1 | **9.2** | **14.6** | **9.1** | **14.5** | **10.0** |
| *FGT* | | GRMZM2G074631 | **15.4** | **13.3** | **26.5** | **26.6** | **12.8** | 78.2 | 48.3 | 49.1 | 62.6 | 78.6 | 86.2 | 101.9 | 88.1 | 85.2 | 95.7 |
| *FGT* | | GRMZM2G161625 | 0.0 | 3.6 | 1.0 | 3.2 | 4.2 | 5.2 | 3.9 | 7.6 | 4.1 | 4.9 | **18.4** | **5.4** | **2.7** | **4.9** | **4.4** |
| *FGT* | | GRMZM2G325023 | **4.4** | **4.2** | **2.3** | **3.7** | **3.7** | 3.9 | 5.8 | 3.0 | 3.7 | 5.0 | 1.0 | 0.9 | 1.8 | 1.4 | 1.3 |
| *3GT* | | GRMZM2G022266 | **110.6** | **157.9** | **128.6** | **106.5** | **134.8** | 110.3 | 95.1 | 91.7 | 124.3 | 95.9 | 91.5 | 75.3 | 72.9 | 102.9 | 117.8 |
| *3GT* | | GRMZM2G063550 | **4.9** | **5.7** | **2.1** | **10.3** | **15.9** | 2.0 | 0.6 | 1.2 | 0.2 | 3.1 | 1.0 | 1.6 | 1.4 | 1.2 | 0.3 |
| *3GT* | | GRMZM2G383404 | **23.2** | **21.6** | **30.7** | **17.7** | **19.9** | 21.7 | 17.2 | 16.5 | 21.7 | 23.4 | 14.9 | 13.9 | 16.6 | 9.7 | 15.4 |
|  | |  |  |  |  |  |  |  |  |  |  |  |  |  |  |  |  |
| **Lignin biosynthesis** | | |  |  |  |  |  |  |  |  |  |  |  |  |  |  |  |
| **Gene** | **Maize gene ID** | | **1.L** | **4.L** | **6.L** | **7.L** | **9.L** | **10.M** | **12.M** | **13.M** | **17.M** | **18.M** | **20.H** | **26.H** | **27.H** | **29.H** | **30.H** |
| *HCT10* | GRMZM2G034360 | | 13.8 | 14.8 | 16.0 | 15.6 | 10.7 | 13.0 | 17.3 | 12.8 | 21.5 | 15.6 | **27.0** | **22.1** | **29.5** | **23.7** | **20.8** |
| *HCT6* | GRMZM2G035584 | | **164.8** | **151.0** | **174.4** | **147.6** | **132.7** | 140.1 | 123.8 | 139.0 | 134.0 | 149.6 | 148.3 | 132.8 | 107.0 | 112.2 | 98.0 |
| *HCT* | GRMZM2G122503 | | **28.4** | **39.6** | **16.0** | **15.2** | **11.5** | 17.8 | 6.8 | 6.1 | 6.2 | 6.0 | 5.3 | 3.4 | 3.1 | 4.7 | 1.2 |
| *HCT* | GRMZM2G124066 | | **18.8** | **17.7** | **24.4** | **24.1** | **31.1** | 19.7 | 13.7 | 14.8 | 14.2 | 28.3 | 18.3 | 11.6 | 15.4 | 11.0 | 14.2 |
| *HCT13* | GRMZM2G129266 | | **166.3** | **91.2** | **81.2** | **80.1** | **110.3** | 101.8 | 91.0 | 75.5 | 66.9 | 75.4 | 86.6 | 63.6 | 73.1 | 56.4 | 58.0 |
| *HCT11* | GRMZM2G156296 | | **109.5** | **47.8** | **46.7** | **83.0** | **89.7** | 97.2 | 73.4 | 72.5 | 60.0 | 61.2 | 60.0 | 44.6 | 28.6 | 52.7 | 29.7 |
| *HCT12* | GRMZM2G179703 | | 60.6 | 32.6 | 54.4 | 66.7 | 53.7 | 78.9 | 88.5 | 74.3 | 71.6 | 99.6 | 70.6 | 141.0 | 94.5 | 63.0 | 68.7 |
| *CCR* | GRMZM2G009681 | | **111.5** | **167.4** | **277.6** | **273.2** | **150.3** | 119.6 | 87.3 | 102.8 | 179.1 | 157.6 | 46.9 | 94.7 | 103.8 | 131.5 | 197.9 |
| *CCR* | GRMZM2G168893 | | 10.3 | 13.0 | 12.4 | 11.1 | 10.0 | 8.6 | 8.4 | 13.3 | 6.6 | 13.5 | **14.1** | **19.7** | **15.0** | **16.6** | **18.6** |
| *OMT3* | GRMZM2G077486 | | **181.4** | **152.4** | **190.0** | **225.9** | **170.3** | 161.5 | 134.6 | 148.2 | 163.5 | 117.4 | 123.4 | 100.1 | 113.3 | 117.1 | 123.2 |

Table S6a. (cont.) Population level normalized counts for highland vs. lowland DE genes involved in phenylpropanoid, flavonoid, and lignin biosynthesis, and nucleotide-sugar interconversion

| **Lignin biosynthesis** | | | | | | | | | | | | | | | | | |
| --- | --- | --- | --- | --- | --- | --- | --- | --- | --- | --- | --- | --- | --- | --- | --- | --- | --- |
| **Gene** | **Maize gene ID** | | **1.L** | **4.L** | **6.L** | **7.L** | **9.L** | **10.M** | **12.M** | **13.M** | **17.M** | **18.M** | **20.H** | **26.H** | **27.H** | **29.H** | **30.H** |
| *ALDH2* | GRMZM2G125268 | | **19.6** | **24.6** | **24.8** | **28.2** | **23.6** | 42.4 | 15.5 | 18.6 | 22.1 | 19.1 | 17.6 | 9.8 | 13.6 | 12.0 | 10.4 |
| *CAD* | GRMZM2G118610 | | **893.9** | **1314.2** | **1073.7** | **1564.3** | **961.6** | 1054.7 | 1052.0 | 1307.3 | 943.2 | 1107.2 | 707.3 | 807.2 | 836.8 | 664.6 | 1157.5 |
|  |  | |  |  |  |  |  |  |  |  |  |  |  |  |  |  |  |
| **Plant nucleotide-sugar interconversion** | | | | | | | | | | | | | | | | | |
| **Gene** | | **Maize gene ID** | **1.L** | **4.L** | **6.L** | **7.L** | **9.L** | **10.M** | **12.M** | **13.M** | **17.M** | **18.M** | **20.H** | **26.H** | **27.H** | **29.H** | **30.H** |
| *RHS1 (SM1)* | | GRMZM2G031311 | **621.0** | **457.0** | **642.0** | **644.4** | **704.9** | 506.8 | 471.7 | 346.3 | 468.1 | 576.3 | 406.2 | 422.7 | 500.0 | 345.4 | 433.2 |
| *UXS* | | GRMZM2G044027 | **232.6** | **240.2** | **360.0** | **331.7** | **258.1** | 279.1 | 286.8 | 232.4 | 239.7 | 324.0 | 239.9 | 203.7 | 273.1 | 164.3 | 233.9 |

Table S6b. Population level normalized counts for highland vs. midland DE genes involved in phenylpropanoid, flavonoid, and lignin biosynthesis, and nucleotide-sugar interconversion

| **Phenylpropanoid and flavonoid biosynthesis** | | | | | | | | | | | | | | | | |
| --- | --- | --- | --- | --- | --- | --- | --- | --- | --- | --- | --- | --- | --- | --- | --- | --- |
| **Gene** | **Maize gene ID** | **1.L** | **4.L** | **6.L** | **7.L** | **9.L** | **10.M** | **12.M** | **13.M** | **17.M** | **18.M** | **20.H** | **26.H** | **27.H** | **29.H** | **30.H** |
| *4CL1* | GRMZM2G055320 | 251.9 | 210.6 | 282.7 | 264.9 | 245.6 | **428.5** | **359.3** | **322.8** | **261.0** | **292.7** | 265.1 | 237.1 | 248.2 | 254.6 | 287.8 |
| *F3H2* | GRMZM2G050234 | 25.6 | 24.6 | 30.3 | 18.2 | 15.1 | **24.9** | **35.4** | **30.2** | **24.3** | **20.8** | 17.6 | 14.0 | 5.0 | 8.3 | 14.9 |
| *FNS1 (FNSI-1)* | GRMZM2G099467 | 464.6 | 257.5 | 371.1 | 339.1 | 336.7 | **312.9** | **425.4** | **336.8** | **291.6** | **363.6** | 253.2 | 322.7 | 240.1 | 186.0 | 182.5 |
| *FLS2* | GRMZM2G069298 | 6.8 | 9.7 | 17.9 | 3.8 | 3.3 | 5.9 | 0.6 | 1.3 | 2.8 | 4.1 | **8.5** | **12.7** | **18.8** | **8.2** | **16.1** |
| *FGT* | GRMZM2G325023 | 4.4 | 4.2 | 2.3 | 3.7 | 3.7 | **3.9** | **5.8** | **3.0** | **3.7** | **5.0** | 1.0 | 0.9 | 1.8 | 1.4 | 1.3 |
| *-* | GRMZM2G007053 | 17.6 | 13.3 | 20.7 | 15.0 | 16.3 | **47.3** | **55.2** | **74.8** | **73.5** | **51.0** | 77.6 | 23.9 | 29.5 | 16.6 | 13.2 |
| *LDOX* | GRMZM2G162158 | 34.2 | 36.1 | 37.3 | 21.6 | 38.4 | **43.6** | **28.8** | **38.9** | **55.7** | **29.3** | 26.1 | 14.6 | 29.5 | 16.8 | 24.3 |
| *3GT* | GRMZM2G135722 | 603.0 | 53.7 | 39.6 | 40.8 | 275.4 | 48.9 | 82.6 | 35.5 | 39.2 | 55.7 | **75.9** | **166.7** | **259.6** | **751.8** | **35.7** |
|  |  |  |  |  |  |  |  |  |  |  |  |  |  |  |  |  |

Table S6b. (cont.) Population level normalized counts for highland vs. midland DE genes involved in phenylpropanoid, flavonoid, and lignin biosynthesis, and nucleotide-sugar interconversion

| **Lignin biosynthesis** | | | | | | | | | | | | | | | | |
| --- | --- | --- | --- | --- | --- | --- | --- | --- | --- | --- | --- | --- | --- | --- | --- | --- |
| **Gene** | **Maize gene ID** | **1.L** | **4.L** | **6.L** | **7.L** | **9.L** | **10.M** | **12.M** | **13.M** | **17.M** | **18.M** | **20.H** | **26.H** | **27.H** | **29.H** | **30.H** |
| *HCT10* | GRMZM2G034360 | 13.8 | 14.8 | 16.0 | 15.6 | 10.7 | 13.0 | 17.3 | 12.8 | 21.5 | 15.6 | **27.0** | **22.1** | **29.5** | **23.7** | **20.8** |
| *HCT11* | GRMZM2G156296 | 109.5 | 47.8 | 46.7 | 83.0 | 89.7 | **97.2** | **73.4** | **72.5** | **60.0** | **61.2** | 60.0 | 44.6 | 28.6 | 52.7 | 29.7 |
| *CCR3* | GRMZM2G131205 | 1212.2 | 1020.0 | 1210.2 | 1400.8 | 1439.6 | 1073.9 | 978.8 | 1059.4 | 838.3 | 906.5 | **1585.6** | **1753.6** | **1691.4** | **1163.6** | **1515.5** |
| *CCR* | GRMZM2G168893 | 10.3 | 13.0 | 12.4 | 11.1 | 10.0 | 8.6 | 8.4 | 13.3 | 6.6 | 13.5 | **14.1** | **19.7** | **15.0** | **16.6** | **18.6** |
| *ALDH5* | GRMZM2G097706 | 127.3 | 242.2 | 257.9 | 202.7 | 171.9 | **234.1** | **276.1** | **276.1** | **319.4** | **306.2** | 205.7 | 169.1 | 159.2 | 238.1 | 101.2 |
| *ALDH2* | GRMZM2G125268 | 19.6 | 24.6 | 24.8 | 28.2 | 23.6 | **42.4** | **15.5** | **18.6** | **22.1** | **19.1** | 17.6 | 9.8 | 13.6 | 12.0 | 10.4 |
| *COMT2* | GRMZM2G082007 | 751.9 | 665.0 | 821.6 | 738.9 | 620.9 | 700.2 | 669.5 | 620.4 | 580.8 | 639.3 | **737.3** | **776.4** | **771.1** | **818.5** | **786.3** |
|  |  |  |  |  |  |  |  |  |  |  |  |  |  |  |  |  |
| **Plant nucleotide-sugar interconversion** | | | | | | | | | | | | | | | | |
| **Gene** | **Maize gene ID** | **1.L** | **4.L** | **6.L** | **7.L** | **9.L** | **10.M** | **12.M** | **13.M** | **17.M** | **18.M** | **20.H** | **26.H** | **27.H** | **29.H** | **30.H** |
| *RHM3* | GRMZM2G044281 | 161.0 | 114.1 | 131.3 | 135.2 | 107.5 | **134.0** | **177.2** | **167.2** | **188.6** | **197.1** | 145.7 | 78.6 | 101.9 | 105.4 | 91.2 |
| *RHM2* | GRMZM2G072911 | 179.0 | 188.6 | 193.3 | 177.5 | 169.1 | **171.9** | **194.9** | **202.0** | **184.1** | **185.6** | 172.3 | 156.8 | 175.5 | 156.3 | 159.2 |
| *UGD* | GRMZM2G328500 | 492.0 | 402.3 | 687.9 | 598.3 | 482.7 | **611.4** | **655.6** | **734.7** | **665.0** | **716.5** | 420.4 | 409.6 | 474.0 | 325.0 | 508.6 |

Table S6c. Population level normalized counts for midland vs. lowland DE genes involved in phenylpropanoid, flavonoid, and lignin biosynthesis, and nucleotide-sugar interconversion

| **Phenylpropanoid and flavonoid biosynthesis** | | | | | | | | | | | | | | | | |
| --- | --- | --- | --- | --- | --- | --- | --- | --- | --- | --- | --- | --- | --- | --- | --- | --- |
| **Gene** | **Maize gene ID** | **1.L** | **4.L** | **6.L** | **7.L** | **9.L** | **10.M** | **12.M** | **13.M** | **17.M** | **18.M** | **20.H** | **26.H** | **27.H** | **29.H** | **30.H** |
| *BX8* | GRMZM2G085054 | 553.6 | 822.7 | 742.1 | 670.9 | 639.3 | **1039.5** | **889.7** | **769.9** | **1012.4** | **771.3** | 690.4 | 828.6 | 773.8 | 823.8 | 789.4 |
| *C4H* | GRMZM2G028677 | 5.6 | 6.1 | 12.4 | 4.3 | 5.7 | **16.5** | **17.0** | **16.1** | **13.2** | **8.6** | 36.3 | 20.0 | 20.2 | 18.3 | 39.8 |
| *4CL* | GRMZM5G805585 | **165.9** | **188.6** | **228.2** | **132.6** | **267.3** | 124.8 | 143.1 | 96.6 | 103.6 | 157.4 | 127.9 | 114.0 | 145.8 | 89.8 | 112.7 |
| *FNSI-2* | GRMZM2G475380 | 2.2 | 2.4 | 6.2 | 2.1 | 6.3 | **6.9** | **9.1** | **12.8** | **5.5** | **6.9** | 6.6 | 5.6 | 7.2 | 0.9 | 5.8 |
| *FLS2* | GRMZM2G069298 | **6.8** | **9.7** | **17.9** | **3.8** | **3.3** | 5.9 | 0.6 | 1.3 | 2.8 | 4.1 | 8.5 | 12.7 | 18.8 | 8.2 | 16.1 |
| *FGT* | GRMZM2G042865 | 2.2 | 4.0 | 2.6 | 4.7 | 2.7 | **18.9** | **7.0** | **14.0** | **8.0** | **7.1** | 9.2 | 14.6 | 9.1 | 14.5 | 10.0 |
| *FGT* | GRMZM2G074631 | 15.4 | 13.3 | 26.5 | 26.6 | 12.8 | **78.2** | **48.3** | **49.1** | **62.6** | **78.6** | 86.2 | 101.9 | 88.1 | 85.2 | 95.7 |

Table S6c. (cont.) Population level normalized counts for midland vs. lowland DE genes involved in phenylpropanoid, flavonoid, and lignin biosynthesis, and nucleotide-sugar interconversion

| **Phenylpropanoid and flavonoid biosynthesis** | | | | | | | | | | | | | | | | | | |
| --- | --- | --- | --- | --- | --- | --- | --- | --- | --- | --- | --- | --- | --- | --- | --- | --- | --- | --- |
| **Gene** | **Maize gene ID** | | **1.L** | | **4.L** | **6.L** | **7.L** | **9.L** | **10.M** | **12.M** | **13.M** | **17.M** | **18.M** | **20.H** | **26.H** | **27.H** | **29.H** | **30.H** |
| *3GT* | GRMZM2G063550 | | **4.9** | | **5.7** | **2.1** | **10.3** | **15.9** | 2.0 | 0.6 | 1.2 | 0.2 | 3.1 | 1.0 | 1.6 | 1.4 | 1.2 | 0.3 |
| *3GT* | GRMZM2G159404 | | **20.8** | | **8.5** | **15.1** | **9.5** | **13.3** | 5.6 | 6.3 | 6.5 | 5.5 | 6.7 | 11.0 | 5.0 | 9.0 | 11.0 | 12.1 |
|  |  | |  | |  |  |  |  |  |  |  |  |  |  |  |  |  |  |
| **Lignin biosynthesis** | | |  | |  |  |  |  |  |  |  |  |  |  |  |  |  |  |
| **Gene** | | **Maize gene ID** | **1.L** | | **4.L** | **6.L** | **7.L** | **9.L** | **10.M** | **12.M** | **13.M** | **17.M** | **18.M** | **20.H** | **26.H** | **27.H** | **29.H** | **30.H** |
| *HCT* | | GRMZM2G043154 | **52.6** | | **23.2** | **29.3** | **31.0** | **29.9** | 16.6 | 20.8 | 19.5 | 13.2 | 16.5 | 10.4 | 19.3 | 22.3 | 50.1 | 12.9 |
| *HCT* | | GRMZM2G122503 | **28.4** | | **39.6** | **16.0** | **15.2** | **11.5** | 17.8 | 6.8 | 6.1 | 6.2 | 6.0 | 5.3 | 3.4 | 3.1 | 4.7 | 1.2 |
| *HCT* | | GRMZM2G177349 | **4.5** | | **14.3** | **5.8** | **5.7** | **3.6** | 0.0 | 1.3 | 0.6 | 2.2 | 2.0 | 1.9 | 8.1 | 7.7 | 1.9 | 1.0 |
| *HCT12* | | GRMZM2G179703 | 60.6 | | 32.6 | 54.4 | 66.7 | 53.7 | **78.9** | **88.5** | **74.3** | **71.6** | **99.6** | 70.6 | 141.0 | 94.5 | 63.0 | 68.7 |
| *CCR1* | | GRMZM2G099420 | **34.3** | | **12.5** | **8.5** | **12.9** | **16.7** | 4.7 | 2.6 | 15.5 | 1.8 | 5.3 | 1.6 | 17.2 | 11.1 | 8.7 | 5.3 |
| *CCR* | | GRMZM2G109720 | **233.8** | | **242.2** | **239.5** | **266.1** | **258.7** | 188.4 | 232.1 | 217.0 | 213.2 | 218.9 | 224.6 | 236.9 | 227.6 | 227.7 | 223.2 |
| *CAD* | | GRMZM2G090980 | **838.6** | | **841.1** | **718.7** | **710.1** | **747.0** | 621.0 | 577.7 | 568.3 | 778.5 | 644.9 | 595.9 | 643.0 | 737.3 | 765.8 | 533.9 |
| *OMT3* | | GRMZM2G077486 | **181.4** | | **152.4** | **190.0** | **225.9** | **170.3** | 161.5 | 134.6 | 148.2 | 163.5 | 117.4 | 123.4 | 100.1 | 113.3 | 117.1 | 123.2 |
|  | |  |  | |  |  |  |  |  |  |  |  |  |  |  |  |  |  |
| **Plant nucleotide-sugar interconversion** | | | | | | | | |  |  |  |  |  |  |  |  |  |  |
| **Gene** | | **Maize gene ID** | | **1.L** | **4.L** | **6.L** | **7.L** | **9.L** | **10.M** | **12.M** | **13.M** | **17.M** | **18.M** | **20.H** | **26.H** | **27.H** | **29.H** | **30.H** |
| *RHM3* | | GRMZM2G044281 | | 161.0 | 114.1 | 131.3 | 135.2 | 107.5 | **134.0** | **177.2** | **167.2** | **188.6** | **197.1** | 145.7 | 78.6 | 101.9 | 105.4 | 91.2 |

Note. Normalized counts for genes encoding enzymes involved in phenylpropanoid, flavonoid, and lignin biosynthesis, and plant nucleotide-sugar interconversion were output from DESeq2 for all pairwise comparisons of elevation of landrace origin: a) highland vs. lowland; b) highland vs. midland; and c) midland vs. lowland. Landrace populations are represented by a number.elevation format (i.e. 1.L = population one from the lowlands). For each gene, we made normalized counts bold for the elevation exhibiting the highest expression level.

Table S7a. Library level normalized counts for highland vs. lowland DE genes involved in phenylpropanoid, flavonoid, and lignin biosynthesis, and nucleotide-sugar interconversion

| **Phenylpropanoid and flavonoid biosynthesis** | | | | | | | | | | | | | | | | |
| --- | --- | --- | --- | --- | --- | --- | --- | --- | --- | --- | --- | --- | --- | --- | --- | --- |
| **Gene** | **Maize gene ID** | **1.1.L** | **1.2.L** | **1.3.L** | **4.1.L** | **4.2.L** | **4.3.L** | **6.1.L** | **6.2.L** | **6.3.L** | **7.1.L** | **7.2.L** | **7.3.L** | **9.1.L** | **9.2.L** | **9.3.L** |
| *C4H* | GRMZM2G028677 | 5.3 | 0.0 | 11.4 | 9.8 | 2.9 | 5.5 | 16.4 | 19.8 | 1.2 | 5.4 | 1.9 | 5.6 | 9.5 | 0.8 | 6.8 |
| *4CL* | GRMZM2G091643 | **0.0** | **0.0** | **5.7** | **16.6** | **2.9** | **105.3** | **4.3** | **0.0** | **33.5** | **3.3** | **19.3** | **31.4** | **0.0** | **8.8** | **6.8** |
| *4CL2* | GRMZM2G174574 | **281.9** | **286.7** | **328.3** | **230.6** | **261.7** | **210.6** | **275.5** | **235.9** | **259.7** | **249.0** | **249.7** | **332.7** | **181.8** | **244.0** | **363.7** |
| *4CL* | GRMZM5G805585 | **172.3** | **167.5** | **157.9** | **170.0** | **270.4** | **125.3** | **274.6** | **316.5** | **93.5** | **72.8** | **242.0** | **82.9** | **342.3** | **228.0** | **231.6** |
| *CHS* | GRMZM2G435393 | **13.8** | **2.2** | **0.0** | **4.9** | **1.0** | **3.3** | **3.5** | **3.0** | **1.2** | **2.2** | **1.0** | **1.1** | **1.2** | **2.4** | **2.3** |
| *CHI1* | GRMZM2G155329 | **321.3** | **205.8** | **544.1** | **477.8** | **267.5** | **481.0** | **308.3** | **439.7** | **633.6** | **476.2** | **356.8** | **369.7** | **473.0** | **279.4** | **401.0** |
| *F3H* | GRMZM2G025870 | 38.3 | 19.1 | 19.3 | 53.7 | 37.5 | 12.2 | 25.9 | 15.2 | 19.6 | 35.9 | 24.1 | 14.6 | 39.2 | 37.7 | 20.3 |
| *FNS1 (FNSI-1)* | GRMZM2G099467 | **339.4** | **525.1** | **529.3** | **258.9** | **179.0** | **334.7** | **337.7** | **232.0** | **543.6** | **257.7** | **186.1** | **573.5** | **173.5** | **337.2** | **499.3** |
| *FGT* | GRMZM2G042865 | 1.1 | 0.0 | 5.7 | 4.9 | 4.8 | 2.2 | 0.0 | 3.0 | 4.6 | 3.3 | 2.9 | 7.8 | 2.4 | 5.6 | 0.0 |
| *FGT* | GRMZM2G074631 | **5.3** | **7.9** | **32.9** | **16.6** | **14.4** | **8.9** | **43.2** | **16.7** | **19.6** | **4.3** | **38.6** | **37.0** | **8.3** | **25.7** | **4.5** |
| *FGT* | GRMZM2G161625 | 0.0 | 0.0 | 0.0 | 9.8 | 1.0 | 0.0 | 0.9 | 2.3 | 0.0 | 7.6 | 1.9 | 0.0 | 8.3 | 3.2 | 1.1 |
| *FGT* | GRMZM2G325023 | 4.3 | 6.7 | 2.3 | 4.9 | 1.0 | 6.7 | 3.5 | 2.3 | 1.2 | 2.2 | 1.0 | 7.8 | 4.8 | 0.8 | 5.6 |
| *3GT* | GRMZM2G022266 | **154.3** | **89.9** | **87.5** | **129.0** | **218.4** | **126.4** | **90.7** | **175.0** | **120.0** | **127.2** | **85.8** | **106.4** | **123.6** | **150.9** | **129.9** |
| *3GT* | GRMZM2G063550 | **1.1** | **7.9** | **5.7** | **7.8** | **3.8** | **5.5** | **0.9** | **3.0** | **2.3** | **19.6** | **6.7** | **4.5** | **33.3** | **6.4** | **7.9** |
| *3GT* | GRMZM2G383404 | **8.5** | **15.7** | **45.4** | **5.9** | **26.9** | **32.1** | **19.0** | **32.7** | **40.4** | **20.7** | **13.5** | **19.0** | **16.6** | **13.6** | **29.4** |
|  |  |  |  |  |  |  |  |  |  |  |  |  |  |  |  |  |
| **Gene** | **Maize gene ID** | **10.1.M** | **10.2.M** | **10.3.M** | **12.1.M** | **12.2.M** | **12.3.M** | **13.1.M** | **13.2.M** | **13.3.M** | **17.1.M** | **17.2.M** | **17.3.M** | **18.1.M** | **18.2.M** | **18.3.M** |
| *C4H* | GRMZM2G028677 | 7.9 | 19.4 | 22.0 | 22.2 | 9.7 | 19.1 | 15.6 | 20.7 | 11.8 | 18.6 | 10.5 | 10.4 | 12.4 | 6.9 | 6.5 |
| *4CL* | GRMZM2G091643 | 0.0 | 6.0 | 4.6 | 0.0 | 16.1 | 5.0 | 1.7 | 1.7 | 3.0 | 0.0 | 2.4 | 3.7 | 0.8 | 5.2 | 21.5 |
| *4CL2* | GRMZM2G174574 | 246.0 | 231.4 | 335.0 | 214.6 | 324.0 | 234.2 | 239.0 | 304.3 | 180.6 | 153.7 | 318.1 | 187.2 | 136.3 | 150.2 | 197.8 |
| *4CL* | GRMZM5G805585 | 184.8 | 86.6 | 103.2 | 223.1 | 83.7 | 122.6 | 135.1 | 105.5 | 49.4 | 119.3 | 84.0 | 107.7 | 204.1 | 102.5 | 165.6 |
| *CHS* | GRMZM2G435393 | 2.3 | 0.0 | 1.2 | 0.9 | 0.0 | 0.0 | 0.0 | 0.0 | 2.0 | 0.0 | 1.6 | 1.5 | 5.0 | 1.7 | 0.0 |
| *CHI1* | GRMZM2G155329 | 368.5 | 306.0 | 358.2 | 324.9 | 345.5 | 363.9 | 317.8 | 231.7 | 415.5 | 363.4 | 361.7 | 415.9 | 369.3 | 338.7 | 447.2 |
| *F3H* | GRMZM2G025870 | 76.0 | 14.9 | 35.9 | 43.6 | 18.2 | 22.1 | 52.0 | 20.7 | 18.8 | 22.4 | 25.0 | 21.5 | 57.8 | 17.4 | 2.2 |
| *FNS1 (FNSI-1)* | GRMZM2G099467 | 293.6 | 386.7 | 258.5 | 428.3 | 472.1 | 375.9 | 202.6 | 428.8 | 379.0 | 180.8 | 384.3 | 309.7 | 313.2 | 427.3 | 350.5 |
| *FGT* | GRMZM2G042865 | 6.8 | 10.5 | 39.4 | 4.3 | 9.7 | 7.0 | 6.1 | 11.2 | 24.7 | 5.6 | 8.9 | 9.7 | 9.1 | 7.8 | 4.3 |
| *FGT* | GRMZM2G074631 | 71.4 | 71.7 | 91.6 | 46.2 | 37.6 | 61.3 | 61.5 | 35.4 | 50.3 | 58.7 | 61.4 | 67.6 | 84.3 | 72.1 | 79.6 |

Table S7a. (cont.) Library level normalized counts for highland vs. lowland DE genes involved in phenylpropanoid, flavonoid, and lignin biosynthesis, and nucleotide-sugar interconversion

| **Phenylpropanoid and flavonoid biosynthesis** | | | | | | | | | | | | | | | | | | | | | | | | | | | | | | | |
| --- | --- | --- | --- | --- | --- | --- | --- | --- | --- | --- | --- | --- | --- | --- | --- | --- | --- | --- | --- | --- | --- | --- | --- | --- | --- | --- | --- | --- | --- | --- | --- |
| **Gene** | | **Maize gene ID** | | | **10.1.M** | | **10.2.M** | | **10.3.M** | | **12.1.M** | | **12.2.M** | | **12.3.M** | | **13.1.M** | | **13.2.M** | | **13.3.M** | | **17.1.M** | | **17.2.M** | | **17.3.M** | | **18.1.M** | **18.2.M** | **18.3.M** |
| *FGT* | | GRMZM2G161625 | | | 11.3 | | 3.0 | | 1.2 | | 3.4 | | 3.2 | | 5.0 | | 19.1 | | 1.7 | | 2.0 | | 8.4 | | 0.8 | | 3.0 | | 10.7 | 1.7 | 2.2 |
| *FGT* | | GRMZM2G325023 | | | 3.4 | | 6.0 | | 2.3 | | 7.7 | | 8.6 | | 1.0 | | 2.6 | | 4.3 | | 2.0 | | 4.7 | | 4.8 | | 1.5 | | 1.7 | 2.6 | 10.8 |
| *3GT* | | GRMZM2G022266 | | | 148.5 | | 95.5 | | 86.9 | | 118.0 | | 86.9 | | 80.4 | | 119.5 | | 113.3 | | 42.4 | | 129.5 | | 97.7 | | 145.6 | | 120.6 | 66.0 | 101.1 |
| *3GT* | | GRMZM2G063550 | | | 0.0 | | 1.5 | | 4.6 | | 0.9 | | 0.0 | | 1.0 | | 1.7 | | 0.9 | | 1.0 | | 0.0 | | 0.0 | | 0.7 | | 1.7 | 3.5 | 4.3 |
| *3GT* | | GRMZM2G383404 | | | 14.7 | | 17.9 | | 32.5 | | 12.0 | | 7.5 | | 32.2 | | 17.3 | | 17.3 | | 14.8 | | 17.7 | | 15.3 | | 31.9 | | 16.5 | 23.4 | 30.1 |
|  | |  | | |  | |  | |  | |  | |  | |  | |  | |  | |  | |  | |  | |  | |  |  |  |
| **Gene** | | **Maize gene ID** | | | **20.1.H** | | **20.2.H** | | **20.3.H** | | **26.1.H** | | **26.2.H** | | **26.3.H** | | **27.1.H** | | **27.2.H** | | **27.3.H** | | **29.1.H** | | **29.2.H** | | **29.3.H** | | **30.1.H** | **30.2.H** | **30.3.H** |
| *C4H* | | GRMZM2G028677 | | | **46.7** | | **31.0** | | **31.2** | | **24.9** | | **18.6** | | **16.4** | | **41.3** | | **19.2** | | **0.0** | | **41.3** | | **8.8** | | **4.9** | | **80.9** | **16.8** | **21.6** |
| *4CL* | | GRMZM2G091643 | | | 0.0 | | 2.8 | | 5.5 | | 0.0 | | 2.0 | | 2.7 | | 0.0 | | 2.9 | | 1.0 | | 1.3 | | 1.8 | | 2.9 | | 0.0 | 1.0 | 2.9 |
| *4CL2* | | GRMZM2G174574 | | | 183.6 | | 264.9 | | 231.1 | | 99.5 | | 145.2 | | 159.5 | | 102.8 | | 200.4 | | 142.9 | | 210.3 | | 188.5 | | 158.6 | | 107.6 | 288.9 | 241.0 |
| *4CL* | | GRMZM5G805585 | | | 237.5 | | 75.1 | | 71.0 | | 162.5 | | 102.0 | | 77.5 | | 178.6 | | 117.9 | | 141.0 | | 164.0 | | 23.8 | | 81.7 | | 55.2 | 166.5 | 116.6 |
| *CHS* | | GRMZM2G435393 | | | 0.0 | | 0.0 | | 0.8 | | 1.7 | | 0.0 | | 0.9 | | 1.7 | | 0.0 | | 0.0 | | 0.0 | | 1.8 | | 0.0 | | 0.0 | 0.0 | 1.0 |
| *CHI1* | | GRMZM2G155329 | | | 374.4 | | 320.3 | | 215.4 | | 190.7 | | 402.2 | | 360.0 | | 181.2 | | 395.1 | | 507.8 | | 220.3 | | 299.5 | | 327.9 | | 319.3 | 328.7 | 275.3 |
| *F3H* | | GRMZM2G025870 | | | **48.7** | | **46.0** | | **25.0** | | **63.0** | | **50.0** | | **39.2** | | **85.9** | | **43.1** | | **16.4** | | **63.8** | | **27.3** | | **15.6** | | **61.4** | **29.3** | **21.6** |
| *FNS1 (FNSI-1)* | | GRMZM2G099467 | | | 69.5 | | 254.6 | | 435.6 | | 68.0 | | 201.1 | | 699.0 | | 74.1 | | 229.2 | | 417.1 | | 122.7 | | 210.5 | | 224.8 | | 89.0 | 261.7 | 196.9 |
| *FGT* | | GRMZM2G042865 | | | **9.3** | | **11.3** | | **7.0** | | **7.5** | | **12.8** | | **23.7** | | **5.1** | | **6.7** | | **15.4** | | **10.0** | | **5.3** | | **28.2** | | **7.1** | **4.2** | **18.6** |
| *FGT* | | GRMZM2G074631 | | | 101.6 | | 93.0 | | 64.0 | | 116.9 | | 79.5 | | 109.4 | | 118.0 | | 77.7 | | 68.5 | | 77.6 | | 85.4 | | 92.4 | | 140.5 | 72.2 | 74.5 |
| *FGT* | | GRMZM2G161625 | | | **17.6** | | **24.4** | | **13.3** | | **11.6** | | **0.0** | | **4.6** | | **4.2** | | **1.9** | | **1.9** | | **7.5** | | **6.2** | | **1.0** | | **7.1** | **4.2** | **2.0** |
| *FGT* | | GRMZM2G325023 | | | **0.0** | | **0.0** | | **3.1** | | **0.8** | | **1.0** | | **0.9** | | **1.7** | | **1.0** | | **2.9** | | **1.3** | | **0.9** | | **1.9** | | **0.9** | **1.0** | **2.0** |
| *3GT* | | GRMZM2G022266 | | | 87.1 | | 111.8 | | 75.7 | | 77.9 | | 107.9 | | 40.1 | | 97.7 | | 81.5 | | 39.6 | | 130.2 | | 98.7 | | 79.8 | | 161.9 | 116.2 | 75.4 |
| *3GT* | | GRMZM2G063550 | | | 3.1 | | 0.0 | | 0.0 | | 0.8 | | 3.9 | | 0.0 | | 3.4 | | 0.0 | | 1.0 | | 2.5 | | 0.0 | | 1.0 | | 0.9 | 0.0 | 0.0 |
| *3GT* | | GRMZM2G383404 | | | 8.3 | | 16.9 | | 19.5 | | 7.5 | | 20.6 | | 13.7 | | 15.2 | | 18.2 | | 16.4 | | 8.8 | | 8.8 | | 11.7 | | 16.0 | 10.5 | 19.6 |
|  |  | |  |  | |  | |  | |  | |  | |  | |  | |  | |  | |  | |  | |  | |  |  |  |  |
| **Lignin biosynthesis** | | | | | | | | | | | | | | | | | | | | | | | | | | | | | | | |
| **Gene** | | **Maize gene ID** | | | **1.1.L** | | **1.2.L** | | **1.3.L** | | **4.1.L** | | **4.2.L** | | **4.3.L** | | **6.1.L** | | **6.2.L** | | **6.3.L** | | **7.1.L** | | **7.2.L** | | **7.3.L** | | **9.1.L** | **9.2.L** | **9.3.L** |
| *HCT10* | | GRMZM2G034360 | | | 23.4 | | 11.2 | | 6.8 | | 20.5 | | 18.3 | | 5.5 | | 13.8 | | 12.2 | | 21.9 | | 10.9 | | 13.5 | | 22.4 | | 11.9 | 8.8 | 11.3 |
| *HCT6* | | GRMZM2G035584 | | | **126.6** | | **133.8** | | **234.0** | | **137.8** | | **151.1** | | **164.0** | | **130.4** | | **164.3** | | **228.5** | | **137.0** | | **110.9** | | **194.9** | | **107.0** | **130.8** | **160.4** |
| *HCT* | | GRMZM2G122503 | | | **5.3** | | **76.5** | | **3.4** | | **62.5** | | **38.5** | | **17.7** | | **4.3** | | **20.5** | | **23.1** | | **6.5** | | **15.4** | | **23.5** | | **23.8** | **7.2** | **3.4** |
| *HCT* | | GRMZM2G124066 | | | **19.1** | | **12.4** | | **25.0** | | **14.7** | | **16.4** | | **22.2** | | **21.6** | | **29.7** | | **21.9** | | **26.1** | | **26.0** | | **20.2** | | **35.7** | **32.9** | **24.9** |
| *HCT13* | | GRMZM2G129266 | | | **104.3** | | **298.0** | | **96.6** | | **86.0** | | **77.9** | | **109.7** | | **98.4** | | **49.5** | | **95.8** | | **98.9** | | **81.0** | | **60.5** | | **110.5** | **133.3** | **87.0** |

Table S7a. (cont.) Library level normalized counts for highland vs. lowland DE genes involved in phenylpropanoid, flavonoid, and lignin biosynthesis, and nucleotide-sugar interconversion

| **Lignin biosynthesis** | | | | | | | | | | | | | | | | |
| --- | --- | --- | --- | --- | --- | --- | --- | --- | --- | --- | --- | --- | --- | --- | --- | --- |
| **Gene** | **Maize gene ID** | **1.1.L** | **1.2.L** | **1.3.L** | **4.1.L** | **4.2.L** | **4.3.L** | **6.1.L** | **6.2.L** | **6.3.L** | **7.1.L** | **7.2.L** | **7.3.L** | **9.1.L** | **9.2.L** | **9.3.L** |
| *HCT11* | GRMZM2G156296 | **154.3** | **63.0** | **111.3** | **46.9** | **21.2** | **75.4** | **27.6** | **59.3** | **53.1** | **57.6** | **78.1** | **113.1** | **64.2** | **118.0** | **87.0** |
| *HCT12* | GRMZM2G179703 | 39.4 | 64.1 | 78.4 | 38.1 | 37.5 | 22.2 | 21.6 | 34.2 | 107.3 | 87.0 | 68.5 | 44.8 | 60.6 | 58.6 | 41.8 |
| *CCR* | GRMZM2G009681 | **94.7** | **102.3** | **137.4** | **215.9** | **136.6** | **149.6** | **219.4** | **389.5** | **223.9** | **299.0** | **195.7** | **324.8** | **60.6** | **253.7** | **136.7** |
| *CCR* | GRMZM2G168893 | 9.6 | 15.7 | 5.7 | 7.8 | 20.2 | 11.1 | 17.3 | 10.7 | 9.2 | 15.2 | 13.5 | 4.5 | 15.4 | 5.6 | 9.0 |
| *OMT3* | GRMZM2G077486 | **164.9** | **220.4** | **159.0** | **145.6** | **159.7** | **151.9** | **152.0** | **194.0** | **223.9** | **254.4** | **228.5** | **194.9** | **196.1** | **156.5** | **158.1** |
| *ALDH2* | GRMZM2G125268 | **16.0** | **23.6** | **19.3** | **13.7** | **26.9** | **33.3** | **25.9** | **22.1** | **26.5** | **26.1** | **32.8** | **25.8** | **30.9** | **18.5** | **21.5** |
| *CAD* | GRMZM2G118610 | **810.7** | **755.6** | **1115.4** | **1655.1** | **694.8** | **1592.8** | **1188.3** | **1256.1** | **776.7** | **1171.0** | **2104.8** | **1417.0** | **1077.9** | **869.4** | **937.6** |
|  |  |  |  |  |  |  |  |  |  |  |  |  |  |  |  |  |
| **Gene** | **Maize gene ID** | **10.1.M** | **10.2.M** | **10.3.M** | **12.1.M** | **12.2.M** | **12.3.M** | **13.1.M** | **13.2.M** | **13.3.M** | **17.1.M** | **17.2.M** | **17.3.M** | **18.1.M** | **18.2.M** | **18.3.M** |
| *HCT10* | GRMZM2G034360 | 18.1 | 10.5 | 10.4 | 8.5 | 17.2 | 26.1 | 13.9 | 8.6 | 15.8 | 22.4 | 26.6 | 15.6 | 18.2 | 15.6 | 12.9 |
| *HCT6* | GRMZM2G035584 | 137.2 | 110.5 | 172.7 | 79.5 | 151.3 | 140.7 | 116.0 | 195.4 | 105.6 | 133.2 | 111.4 | 157.5 | 138.8 | 157.2 | 152.7 |
| *HCT* | GRMZM2G122503 | 20.4 | 7.5 | 25.5 | 4.3 | 2.1 | 14.1 | 1.7 | 7.8 | 8.9 | 0.0 | 9.7 | 8.9 | 11.6 | 4.3 | 2.2 |
| *HCT* | GRMZM2G124066 | 23.8 | 22.4 | 12.8 | 17.1 | 15.0 | 9.0 | 10.4 | 17.3 | 16.8 | 15.8 | 11.3 | 15.6 | 24.0 | 28.7 | 32.3 |
| *HCT13* | GRMZM2G129266 | 171.2 | 65.7 | 68.4 | 106.9 | 82.6 | 83.4 | 76.2 | 82.1 | 68.1 | 46.6 | 67.8 | 86.2 | 100.8 | 73.8 | 51.6 |
| *HCT11* | GRMZM2G156296 | 97.5 | 137.3 | 56.8 | 53.0 | 114.8 | 52.3 | 57.2 | 128.8 | 31.6 | 64.3 | 46.0 | 69.8 | 86.8 | 58.2 | 38.7 |
| *HCT12* | GRMZM2G179703 | 123.6 | 70.2 | 42.9 | 107.7 | 65.5 | 92.5 | 55.4 | 72.6 | 94.8 | 81.1 | 46.8 | 86.9 | 124.8 | 64.3 | 109.7 |
| *CCR* | GRMZM2G009681 | 165.5 | 82.1 | 111.3 | 52.1 | 121.2 | 88.5 | 94.4 | 85.6 | 128.3 | 172.4 | 160.7 | 204.3 | 204.1 | 135.5 | 133.3 |
| *CCR* | GRMZM2G168893 | 9.1 | 7.5 | 9.3 | 9.4 | 9.7 | 6.0 | 13.9 | 11.2 | 14.8 | 6.5 | 7.3 | 5.9 | 14.0 | 11.3 | 15.1 |
| *OMT3* | GRMZM2G077486 | 209.7 | 165.7 | 109.0 | 100.9 | 150.2 | 152.8 | 215.6 | 111.5 | 117.5 | 209.7 | 147.8 | 133.0 | 123.9 | 112.0 | 116.1 |
| *ALDH2* | GRMZM2G125268 | 15.9 | 10.5 | 100.8 | 19.7 | 12.9 | 14.1 | 18.2 | 13.0 | 24.7 | 13.0 | 24.2 | 29.0 | 14.0 | 17.4 | 25.8 |
| *CAD* | GRMZM2G118610 | 1089.5 | 564.3 | 1510.3 | 659.1 | 1355.2 | 1141.8 | 938.8 | 695.1 | 2287.9 | 847.0 | 540.2 | 1442.4 | 1289.8 | 651.3 | 1380.4 |
|  |  |  |  |  |  |  |  |  |  |  |  |  |  |  |  |  |
| **Gene** | **Maize gene ID** | **20.1.H** | **20.2.H** | **20.3.H** | **26.1.H** | **26.2.H** | **26.3.H** | **27.1.H** | **27.2.H** | **27.3.H** | **29.1.H** | **29.2.H** | **29.3.H** | **30.1.H** | **30.2.H** | **30.3.H** |
| *HCT10* | GRMZM2G034360 | **19.7** | **26.3** | **35.1** | **32.3** | **16.7** | **17.3** | **39.6** | **32.6** | **16.4** | **16.3** | **35.2** | **19.5** | **12.5** | **32.5** | **17.6** |
| *HCT6* | GRMZM2G035584 | 175.3 | 122.1 | 147.5 | 121.9 | 147.1 | 129.4 | 104.5 | 110.3 | 106.2 | 135.2 | 83.7 | 117.7 | 96.1 | 108.9 | 89.2 |
| *HCT* | GRMZM2G122503 | 4.1 | 5.6 | 6.2 | 2.5 | 4.9 | 2.7 | 2.5 | 1.0 | 5.8 | 7.5 | 2.6 | 3.9 | 2.7 | 1.0 | 0.0 |
| *HCT* | GRMZM2G124066 | 19.7 | 18.8 | 16.4 | 6.6 | 22.6 | 5.5 | 12.6 | 16.3 | 17.4 | 11.3 | 14.1 | 7.8 | 12.5 | 9.4 | 20.6 |
| *HCT13* | GRMZM2G129266 | 139.0 | 73.3 | 47.6 | 39.0 | 60.8 | 91.1 | 54.8 | 99.7 | 64.7 | 61.3 | 57.3 | 50.6 | 57.8 | 56.5 | 59.8 |
| *HCT11* | GRMZM2G156296 | 49.8 | 87.4 | 42.9 | 28.2 | 61.8 | 43.7 | 25.3 | 31.6 | 29.0 | 52.6 | 60.8 | 44.8 | 24.9 | 38.7 | 25.5 |
| *HCT12* | GRMZM2G179703 | **60.1** | **88.3** | **63.2** | **150.1** | **160.9** | **112.1** | **76.7** | **101.6** | **105.2** | **63.8** | **58.1** | **67.1** | **117.4** | **52.3** | **36.3** |
| *CCR* | GRMZM2G009681 | 28.0 | 71.4 | 41.4 | 92.9 | 75.5 | 115.7 | 77.5 | 108.4 | 125.5 | 152.7 | 126.8 | 114.8 | 100.5 | 310.9 | 182.3 |
| *CCR* | GRMZM2G168893 | **14.5** | **16.9** | **10.9** | **23.2** | **17.7** | **18.2** | **22.7** | **13.4** | **8.7** | **23.8** | **12.3** | **13.6** | **15.1** | **23.0** | **17.6** |
| *OMT3* | GRMZM2G077486 | 101.6 | 124.9 | 143.6 | 119.4 | 72.6 | 108.5 | 84.3 | 148.6 | 107.2 | 130.2 | 117.2 | 104.1 | 173.5 | 94.2 | 101.9 |
| *ALDH2* | GRMZM2G125268 | 9.3 | 26.3 | 17.2 | 7.5 | 11.8 | 10.0 | 11.0 | 20.1 | 9.7 | 2.5 | 15.0 | 18.5 | 5.3 | 8.4 | 17.6 |

Table S7a. (cont.) Library level normalized counts for highland vs. lowland DE genes involved in phenylpropanoid, flavonoid, and lignin biosynthesis, and nucleotide-sugar interconversion

| **Lignin biosynthesis** | | | | | | | | | | | | | | | | |
| --- | --- | --- | --- | --- | --- | --- | --- | --- | --- | --- | --- | --- | --- | --- | --- | --- |
| **Gene** | **Maize gene ID** | **20.1.H** | **20.2.H** | **20.3.H** | **26.1.H** | **26.2.H** | **26.3.H** | **27.1.H** | **27.2.H** | **27.3.H** | **29.1.H** | **29.2.H** | **29.3.H** | **30.1.H** | **30.2.H** | **30.3.H** |
| *CAD* | GRMZM2G118610 | 423.1 | 810.6 | 888.3 | 593.6 | 923.1 | 905.0 | 654.7 | 823.7 | 1032.0 | 266.6 | 955.7 | 771.6 | 998.0 | 832.3 | 1642.2 |

| **Plant nucleotide-sugar interconversion enzymes** | | | | | | | | | | | | | | | | |
| --- | --- | --- | --- | --- | --- | --- | --- | --- | --- | --- | --- | --- | --- | --- | --- | --- |
| **Gene** | **Maize gene ID** | **1.1.L** | **1.2.L** | **1.3.L** | **4.1.L** | **4.2.L** | **4.3.L** | **6.1.L** | **6.2.L** | **6.3.L** | **7.1.L** | **7.2.L** | **7.3.L** | **9.1.L** | **9.2.L** | **9.3.L** |
| *RHS1 (SM1)* | GRMZM2G031311 | **451.1** | **564.4** | **847.4** | **555.9** | **383.9** | **431.2** | **404.2** | **820.2** | **701.7** | **729.5** | **689.4** | **514.2** | **1056.5** | **530.6** | **527.5** |
| *UXS* | GRMZM2G044027 | **196.8** | **220.4** | **280.6** | **249.2** | **216.5** | **254.9** | **361.8** | **287.6** | **430.5** | **365.3** | **255.5** | **374.1** | **325.6** | **207.9** | **240.6** |
|  |  |  |  |  |  |  |  |  |  |  |  |  |  |  |  |  |
| **Gene** | **Maize gene ID** | **10.1.M** | **10.2.M** | **10.3.M** | **12.1.M** | **12.2.M** | **12.3.M** | **13.1.M** | **13.2.M** | **13.3.M** | **17.1.M** | **17.2.M** | **17.3.M** | **18.1.M** | **18.2.M** | **18.3.M** |
| *RHS1 (SM1)* | GRMZM2G031311 | 408.1 | 443.4 | 668.8 | 730.1 | 342.3 | 342.8 | 292.7 | 257.6 | 488.6 | 433.3 | 280.2 | 690.7 | 661.8 | 544.5 | 522.5 |
| *UXS* | GRMZM2G044027 | 290.2 | 232.9 | 314.1 | 207.7 | 355.2 | 297.5 | 230.4 | 243.8 | 223.1 | 219.9 | 268.9 | 230.2 | 285.9 | 301.4 | 384.9 |
|  |  |  |  |  |  |  |  |  |  |  |  |  |  |  |  |  |
| **Gene** | **Maize gene ID** | **20.1.H** | **20.2.H** | **20.3.H** | **26.1.H** | **26.2.H** | **26.3.H** | **27.1.H** | **27.2.H** | **27.3.H** | **29.1.H** | **29.2.H** | **29.3.H** | **30.1.H** | **30.2.H** | **30.3.H** |
| *RHS1* *(SM1)* | GRMZM2G031311 | 559.0 | 287.4 | 372.3 | 366.5 | 606.2 | 295.3 | 482.8 | 480.4 | 536.8 | 331.7 | 259.0 | 445.6 | 607.5 | 312.0 | 380.2 |
| *UXS* | GRMZM2G044027 | 248.9 | 249.9 | 220.9 | 203.1 | 251.1 | 156.8 | 275.5 | 238.8 | 305.1 | 154.0 | 146.2 | 192.7 | 179.7 | 272.2 | 249.9 |

Table S7b. Library level normalized counts for highland vs. midland DE genes involved in phenylpropanoid, flavonoid, and lignin biosynthesis, and nucleotide-sugar interconversion

| **Phenylpropanoid and flavonoid biosynthesis** | | | | |  |  |  |  |  |  |  |  |  |  |  |  |
| --- | --- | --- | --- | --- | --- | --- | --- | --- | --- | --- | --- | --- | --- | --- | --- | --- |
| **Gene** | **Maize gene ID** | **1.1.L** | **1.2.L** | **1.3.L** | **4.1.L** | **4.2.L** | **4.3.L** | **6.1.L** | **6.2.L** | **6.3.L** | **7.1.L** | **7.2.L** | **7.3.L** | **9.1.L** | **9.2.L** | **9.3.L** |
| *4CL1* | GRMZM2G055320 | 100.0 | 191.1 | 464.6 | 128.0 | 141.5 | 362.4 | 136.4 | 206.2 | 505.5 | 109.8 | 211.2 | 473.8 | 96.3 | 118.8 | 521.9 |
| *F3H2* | GRMZM2G050234 | 6.4 | 4.5 | 65.9 | 10.7 | 22.1 | 41.0 | 20.7 | 13.7 | 56.6 | 3.3 | 14.5 | 37.0 | 8.3 | 8.8 | 28.2 |
| *FNS1 (FNSI-1)* | GRMZM2G099467 | 339.4 | 525.1 | 529.3 | 258.9 | 179.0 | 334.7 | 337.7 | 232.0 | 543.6 | 257.7 | 186.1 | 573.5 | 173.5 | 337.2 | 499.3 |
| *FLS2* | GRMZM2G069298 | 18.1 | 0.0 | 2.3 | 13.7 | 15.4 | 0.0 | 3.5 | 34.2 | 16.2 | 3.3 | 5.8 | 2.2 | 8.3 | 1.6 | 0.0 |
| *FGT* | GRMZM2G325023 | 4.3 | 6.7 | 2.3 | 4.9 | 1.0 | 6.7 | 3.5 | 2.3 | 1.2 | 2.2 | 1.0 | 7.8 | 4.8 | 0.8 | 5.6 |
| *-* | GRMZM2G007053 | 25.5 | 10.1 | 17.0 | 11.7 | 11.5 | 16.6 | 24.2 | 18.3 | 19.6 | 19.6 | 16.4 | 9.0 | 9.5 | 22.5 | 16.9 |

Table S7b. (cont.) Library level normalized counts for highland vs. midland DE genes involved in phenylpropanoid, flavonoid, and lignin biosynthesis, and nucleotide-sugar interconversion

| **Phenylpropanoid and flavonoid biosynthesis** | | | | | | | | | | | | | | | | |
| --- | --- | --- | --- | --- | --- | --- | --- | --- | --- | --- | --- | --- | --- | --- | --- | --- |
| **Gene** | **Maize gene ID** | **1.1.L** | **1.2.L** | **1.3.L** | **4.1.L** | **4.2.L** | **4.3.L** | **6.1.L** | **6.2.L** | **6.3.L** | **7.1.L** | **7.2.L** | **7.3.L** | **9.1.L** | **9.2.L** | **9.3.L** |
| *LDOX* | GRMZM2G162158 | 38.3 | 31.5 | 32.9 | 61.6 | 28.9 | 17.7 | 48.4 | 22.1 | 41.5 | 22.8 | 17.4 | 24.6 | 52.3 | 45.0 | 18.1 |
| *3GT* | GRMZM2G135722 | 1568.1 | 221.5 | 19.3 | 31.3 | 78.9 | 51.0 | 85.5 | 13.7 | 19.6 | 25.0 | 35.7 | 61.6 | 48.7 | 751.4 | 26.0 |
|  |  |  |  |  |  |  |  |  |  |  |  |  |  |  |  |  |
| **Gene** | **Maize gene ID** | **10.1.M** | **10.2.M** | **10.3.M** | **12.1.M** | **12.2.M** | **12.3.M** | **13.1.M** | **13.2.M** | **13.3.M** | **17.1.M** | **17.2.M** | **17.3.M** | **18.1.M** | **18.2.M** | **18.3.M** |
| *4CL1* | GRMZM2G055320 | **96.4** | **725.5** | **463.6** | **119.7** | **672.8** | **285.5** | **71.0** | **486.7** | **410.6** | **122.1** | **388.4** | **272.6** | **96.7** | **465.5** | **316.1** |
| *F3H2* | GRMZM2G050234 | **5.7** | **38.8** | **30.1** | **21.4** | **52.6** | **32.2** | **4.3** | **12.1** | **74.0** | **10.2** | **11.3** | **51.2** | **14.9** | **34.7** | **12.9** |
| *FNS1 (FNSI-1)* | GRMZM2G099467 | **293.6** | **386.7** | **258.5** | **428.3** | **472.1** | **375.9** | **202.6** | **428.8** | **379.0** | **180.8** | **384.3** | **309.7** | **313.2** | **427.3** | **350.5** |
| *FLS2* | GRMZM2G069298 | 0.0 | 1.5 | 16.2 | 0.9 | 1.1 | 0.0 | 0.9 | 0.0 | 3.0 | 0.9 | 0.0 | 7.4 | 3.3 | 6.9 | 2.2 |
| *FGT* | GRMZM2G325023 | **3.4** | **6.0** | **2.3** | **7.7** | **8.6** | **1.0** | **2.6** | **4.3** | **2.0** | **4.7** | **4.8** | **1.5** | **1.7** | **2.6** | **10.8** |
| *-* | GRMZM2G007053 | **64.6** | **13.4** | **63.8** | **61.6** | **54.7** | **49.3** | **55.4** | **67.4** | **101.7** | **78.3** | **77.5** | **64.6** | **63.6** | **26.9** | **62.4** |
| *LDOX* | GRMZM2G162158 | **52.2** | **25.4** | **53.3** | **52.1** | **15.0** | **19.1** | **52.8** | **43.2** | **20.7** | **76.4** | **48.4** | **42.3** | **33.1** | **31.3** | **23.7** |
| *3GT* | GRMZM2G135722 | 43.1 | 53.7 | 49.8 | 61.6 | 133.1 | 53.3 | 30.3 | 41.5 | 34.5 | 20.5 | 43.6 | 53.5 | 44.6 | 68.6 | 53.8 |
|  |  |  |  |  |  |  |  |  |  |  |  |  |  |  |  |  |
| **Gene** | **Maize gene ID** | **20.1.H** | **20.2.H** | **20.3.H** | **26.1.H** | **26.2.H** | **26.3.H** | **27.1.H** | **27.2.H** | **27.3.H** | **29.1.H** | **29.2.H** | **29.3.H** | **30.1.H** | **30.2.H** | **30.3.H** |
| *4CL1* | GRMZM2G055320 | 57.0 | 299.6 | 438.7 | 67.2 | 217.8 | 426.5 | 81.7 | 190.8 | 472.1 | 55.1 | 183.2 | 525.4 | 47.1 | 246.0 | 570.3 |
| *F3H2* | GRMZM2G050234 | 5.2 | 28.2 | 19.5 | 0.8 | 15.7 | 25.5 | 1.7 | 7.7 | 5.8 | 0.0 | 3.5 | 21.4 | 2.7 | 28.3 | 13.7 |
| *FNS1 (FNSI-1)* | GRMZM2G099467 | 69.5 | 254.6 | 435.6 | 68.0 | 201.1 | 699.0 | 74.1 | 229.2 | 417.1 | 122.7 | 210.5 | 224.8 | 89.0 | 261.7 | 196.9 |
| *FLS2* | GRMZM2G069298 | **11.4** | **5.6** | **8.6** | **9.1** | **9.8** | **19.1** | **2.5** | **19.2** | **34.8** | **2.5** | **10.6** | **11.7** | **26.7** | **1.0** | **20.6** |
| *FGT* | GRMZM2G325023 | 0.0 | 0.0 | 3.1 | 0.8 | 1.0 | 0.9 | 1.7 | 1.0 | 2.9 | 1.3 | 0.9 | 1.9 | 0.9 | 1.0 | 2.0 |
| *-* | GRMZM2G007053 | 39.4 | 72.3 | 121.0 | 22.4 | 28.4 | 21.0 | 21.9 | 14.4 | 52.1 | 20.0 | 13.2 | 16.5 | 14.2 | 13.6 | 11.8 |
| *LDOX* | GRMZM2G162158 | 9.3 | 15.0 | 53.9 | 10.8 | 4.9 | 28.3 | 40.4 | 21.1 | 27.0 | 27.5 | 5.3 | 17.5 | 28.5 | 20.9 | 23.5 |
| *3GT* | GRMZM2G135722 | **14.5** | **167.2** | **46.1** | **43.1** | **19.6** | **437.5** | **597.4** | **15.3** | **166.1** | **2035.3** | **62.5** | **157.6** | **45.4** | **16.8** | **45.1** |
|  |  |  |  |  |  |  |  |  |  |  |  |  |  |  |  |  |

Table S7b. (cont.) Library level normalized counts for highland vs. midland DE genes involved in phenylpropanoid, flavonoid, and lignin biosynthesis, and nucleotide-sugar interconversion

| **Lignin biosynthesis** | |  |  |  |  |  |  |  |  |  |  |  |  |  |  |  |
| --- | --- | --- | --- | --- | --- | --- | --- | --- | --- | --- | --- | --- | --- | --- | --- | --- |
| **Gene** | **Maize gene ID** | **1.1.L** | **1.2.L** | **1.3.L** | **4.1.L** | **4.2.L** | **4.3.L** | **6.1.L** | **6.2.L** | **6.3.L** | **7.1.L** | **7.2.L** | **7.3.L** | **9.1.L** | **9.2.L** | **9.3.L** |
| *HCT10* | GRMZM2G034360 | 23.4 | 11.2 | 6.8 | 20.5 | 18.3 | 5.5 | 13.8 | 12.2 | 21.9 | 10.9 | 13.5 | 22.4 | 11.9 | 8.8 | 11.3 |
| *HCT11* | GRMZM2G156296 | 154.3 | 63.0 | 111.3 | 46.9 | 21.2 | 75.4 | 27.6 | 59.3 | 53.1 | 57.6 | 78.1 | 113.1 | 64.2 | 118.0 | 87.0 |
| *CCR3* | GRMZM2G131205 | 1616.0 | 898.4 | 1122.3 | 1273.1 | 1100.8 | 686.1 | 1595.1 | 1382.4 | 653.2 | 2218.0 | 1200.4 | 784.1 | 2467.2 | 1223.4 | 628.1 |
| *CCR* | GRMZM2G168893 | 9.6 | 15.7 | 5.7 | 7.8 | 20.2 | 11.1 | 17.3 | 10.7 | 9.2 | 15.2 | 13.5 | 4.5 | 15.4 | 5.6 | 9.0 |
| *ALDH5* | GRMZM2G097706 | 159.6 | 140.5 | 81.8 | 269.7 | 165.5 | 291.5 | 472.4 | 138.5 | 162.7 | 226.1 | 242.0 | 140.0 | 130.7 | 241.6 | 143.5 |
| *ALDH2* | GRMZM2G125268 | 16.0 | 23.6 | 19.3 | 13.7 | 26.9 | 33.3 | 25.9 | 22.1 | 26.5 | 26.1 | 32.8 | 25.8 | 30.9 | 18.5 | 21.5 |
| *COMT2* | GRMZM2G082007 | 764.9 | 723.0 | 767.9 | 719.1 | 635.1 | 640.7 | 946.5 | 909.9 | 608.2 | 790.4 | 786.8 | 639.6 | 708.3 | 610.9 | 543.3 |
|  |  |  |  |  |  |  |  |  |  |  |  |  |  |  |  |  |
| **Gene** | **Maize gene ID** | **10.1.M** | **10.2.M** | **10.3.M** | **12.1.M** | **12.2.M** | **12.3.M** | **13.1.M** | **13.2.M** | **13.3.M** | **17.1.M** | **17.2.M** | **17.3.M** | **18.1.M** | **18.2.M** | **18.3.M** |
| *HCT10* | GRMZM2G034360 | 18.1 | 10.5 | 10.4 | 8.5 | 17.2 | 26.1 | 13.9 | 8.6 | 15.8 | 22.4 | 26.6 | 15.6 | 18.2 | 15.6 | 12.9 |
| *HCT11* | GRMZM2G156296 | **97.5** | **137.3** | **56.8** | **53.0** | **114.8** | **52.3** | **57.2** | **128.8** | **31.6** | **64.3** | **46.0** | **69.8** | **86.8** | **58.2** | **38.7** |
| *CCR3* | GRMZM2G131205 | 2197.1 | 430.0 | 594.6 | 1585.8 | 630.9 | 719.7 | 1884.5 | 308.6 | 985.1 | 1219.7 | 451.3 | 843.8 | 1559.2 | 517.6 | 642.9 |
| *CCR* | GRMZM2G168893 | 9.1 | 7.5 | 9.3 | 9.4 | 9.7 | 6.0 | 13.9 | 11.2 | 14.8 | 6.5 | 7.3 | 5.9 | 14.0 | 11.3 | 15.1 |
| *ALDH5* | GRMZM2G097706 | **227.9** | **79.1** | **395.3** | **359.1** | **107.3** | **361.8** | **202.6** | **370.9** | **254.7** | **191.0** | **369.8** | **397.4** | **314.8** | **255.3** | **348.3** |
| *ALDH2* | GRMZM2G125268 | **15.9** | **10.5** | **100.8** | **19.7** | **12.9** | **14.1** | **18.2** | **13.0** | **24.7** | **13.0** | **24.2** | **29.0** | **14.0** | **17.4** | **25.8** |
| *COMT2* | GRMZM2G082007 | 851.4 | 594.2 | 654.9 | 760.0 | 606.2 | 642.3 | 711.9 | 541.2 | 608.0 | 494.8 | 442.5 | 805.1 | 756.9 | 515.9 | 645.0 |
|  |  |  |  |  |  |  |  |  |  |  |  |  |  |  |  |  |
| **Gene** | **Maize gene ID** | **20.1.H** | **20.2.H** | **20.3.H** | **26.1.H** | **26.2.H** | **26.3.H** | **27.1.H** | **27.2.H** | **27.3.H** | **29.1.H** | **29.2.H** | **29.3.H** | **30.1.H** | **30.2.H** | **30.3.H** |
| *HCT10* | GRMZM2G034360 | **19.7** | **26.3** | **35.1** | **32.3** | **16.7** | **17.3** | **39.6** | **32.6** | **16.4** | **16.3** | **35.2** | **19.5** | **12.5** | **32.5** | **17.6** |
| *HCT11* | GRMZM2G156296 | 49.8 | 87.4 | 42.9 | 28.2 | 61.8 | 43.7 | 25.3 | 31.6 | 29.0 | 52.6 | 60.8 | 44.8 | 24.9 | 38.7 | 25.5 |
| *CCR3* | GRMZM2G131205 | **3094.5** | **1008.8** | **653.4** | **2694.6** | **1860.9** | **705.4** | **3377.0** | **998.2** | **699.0** | **1723.6** | **1143.3** | **623.7** | **2931.9** | **1173.6** | **440.9** |
| *CCR* | GRMZM2G168893 | **14.5** | **16.9** | **10.9** | **23.2** | **17.7** | **18.2** | **22.7** | **13.4** | **8.7** | **23.8** | **12.3** | **13.6** | **15.1** | **23.0** | **17.6** |
| *ALDH5* | GRMZM2G097706 | 106.8 | 193.5 | 316.9 | 247.1 | 136.4 | 123.9 | 111.2 | 84.4 | 281.9 | 336.7 | 240.5 | 137.2 | 67.6 | 167.5 | 68.6 |
| *ALDH2* | GRMZM2G125268 | 9.3 | 26.3 | 17.2 | 7.5 | 11.8 | 10.0 | 11.0 | 20.1 | 9.7 | 2.5 | 15.0 | 18.5 | 5.3 | 8.4 | 17.6 |
| *COMT2* | GRMZM2G082007 | **794.4** | **693.2** | **724.4** | **927.8** | **806.3** | **595.1** | **800.4** | **712.4** | **800.3** | **1038.9** | **773.4** | **643.2** | **1006.0** | **746.4** | **606.5** |
|  |  |  |  |  |  |  |  |  |  |  |  |  |  |  |  |  |
|  | | | | |  |  |  |  |  |  |  |  |  |  |  |  |
| **Plant nucleotide-sugar interconversion enzymes** | | | | | | |  |  |  |  |  |  |  |  |  |  |
| **Gene** | **Maize gene ID** | **1.1.L** | **1.2.L** | **1.3.L** | **4.1.L** | **4.2.L** | **4.3.L** | **6.1.L** | **6.2.L** | **6.3.L** | **7.1.L** | **7.2.L** | **7.3.L** | **9.1.L** | **9.2.L** | **9.3.L** |
| *RHM3* | GRMZM2G044281 | 125.5 | 154.0 | 203.3 | 129.9 | 90.5 | 121.9 | 120.9 | 139.2 | 133.9 | 203.3 | 99.3 | 103.1 | 124.8 | 91.5 | 106.2 |
| *RHM2* | GRMZM2G072911 | 162.8 | 156.3 | 218.1 | 175.9 | 183.8 | 206.2 | 209.9 | 203.9 | 166.2 | 176.1 | 172.6 | 183.7 | 171.1 | 156.5 | 179.6 |
| *UGD* | GRMZM2G328500 | 428.7 | 431.8 | 615.6 | 449.4 | 317.6 | 440.0 | 720.2 | 493.0 | 850.6 | 658.9 | 449.3 | 686.7 | 543.1 | 399.0 | 506.1 |
|  |  |  |  |  |  |  |  |  |  |  |  |  |  |  |  |  |

Table S7b. (cont.) Library level normalized counts for highland vs. midland DE genes involved in phenylpropanoid, flavonoid, and lignin biosynthesis, and nucleotide-sugar interconversion

| **Plant nucleotide-sugar interconversion enzymes** | | | | | | | | | | | | | | | | |
| --- | --- | --- | --- | --- | --- | --- | --- | --- | --- | --- | --- | --- | --- | --- | --- | --- |
| **Gene** | **Maize gene ID** | **10.1.M** | **10.2.M** | **10.3.M** | **12.1.M** | **12.2.M** | **12.3.M** | **13.1.M** | **13.2.M** | **13.3.M** | **17.1.M** | **17.2.M** | **17.3.M** | **18.1.M** | **18.2.M** | **18.3.M** |
| *RHM3* | GRMZM2G044281 | **155.3** | **97.0** | **149.5** | **164.1** | **200.6** | **166.9** | **175.8** | **159.9** | **165.8** | **167.7** | **214.0** | **184.2** | **195.8** | **158.9** | **236.5** |
| *RHM2* | GRMZM2G072911 | **181.4** | **158.2** | **176.2** | **173.5** | **239.3** | **171.9** | **172.3** | **172.0** | **261.6** | **173.3** | **187.3** | **191.6** | **191.7** | **191.1** | **174.2** |
| *UGD* | GRMZM2G328500 | **630.3** | **558.3** | **645.6** | **436.8** | **926.0** | **604.1** | **485.8** | **759.9** | **958.4** | **437.0** | **1043.2** | **514.7** | **647.0** | **747.7** | **754.7** |
|  |  |  |  |  |  |  |  |  |  |  |  |  |  |  |  |  |
| **Gene** | **Maize gene ID** | **20.1.H** | **20.2.H** | **20.3.H** | **26.1.H** | **26.2.H** | **26.3.H** | **27.1.H** | **27.2.H** | **27.3.H** | **29.1.H** | **29.2.H** | **29.3.H** | **30.1.H** | **30.2.H** | **30.3.H** |
| *RHM3* | GRMZM2G044281 | 216.7 | 121.2 | 99.1 | 119.4 | 72.6 | 43.7 | 137.3 | 94.9 | 73.4 | 152.7 | 82.8 | 80.8 | 96.1 | 95.3 | 82.3 |
| *RHM2* | GRMZM2G072911 | 143.1 | 202.0 | 171.7 | 128.5 | 167.7 | 174.1 | 126.4 | 205.2 | 195.0 | 132.7 | 161.2 | 175.1 | 108.5 | 176.9 | 192.1 |
| *UGD* | GRMZM2G328500 | 364.0 | 441.5 | 455.9 | 349.0 | 375.7 | 504.0 | 386.7 | 391.2 | 643.9 | 230.3 | 312.7 | 432.0 | 365.6 | 551.7 | 608.5 |

Table S7c. Library level normalized counts for midland vs. lowland DE genes involved in phenylpropanoid, flavonoid, and lignin biosynthesis, and nucleotide-sugar interconversion

| **Phenylpropanoid and flavonoid biosynthesis** | | | | | |  |  |  |  |  |  |  |  |  |  |  |
| --- | --- | --- | --- | --- | --- | --- | --- | --- | --- | --- | --- | --- | --- | --- | --- | --- |
| **Gene** | **Maize gene ID** | **1.1.L** | **1.2.L** | **1.3.L** | **4.1.L** | **4.2.L** | **4.3.L** | **6.1.L** | **6.2.L** | **6.3.L** | **7.1.L** | **7.2.L** | **7.3.L** | **9.1.L** | **9.2.L** | **9.3.L** |
| *BX8* | GRMZM2G085054 | 371.3 | 425.0 | 864.4 | 589.2 | 567.7 | 1311.2 | 908.5 | 601.0 | 716.7 | 374.0 | 638.3 | 1000.3 | 348.2 | 783.5 | 786.2 |
| *C4H* | GRMZM2G028677 | 5.3 | 0.0 | 11.4 | 9.8 | 2.9 | 5.5 | 16.4 | 19.8 | 1.2 | 5.4 | 1.9 | 5.6 | 9.5 | 0.8 | 6.8 |
| *4CL* | GRMZM5G805585 | **172.3** | **167.5** | **157.9** | **170.0** | **270.4** | **125.3** | **274.6** | **316.5** | **93.5** | **72.8** | **242.0** | **82.9** | **342.3** | **228.0** | **231.6** |
| *FNSI-2* | GRMZM2G475380 | 2.1 | 2.2 | 2.3 | 2.9 | 1.0 | 3.3 | 14.7 | 1.5 | 2.3 | 2.2 | 2.9 | 1.1 | 5.9 | 4.0 | 9.0 |
| *FLS2* | GRMZM2G069298 | **18.1** | **0.0** | **2.3** | **13.7** | **15.4** | **0.0** | **3.5** | **34.2** | **16.2** | **3.3** | **5.8** | **2.2** | **8.3** | **1.6** | **0.0** |
| *FGT* | GRMZM2G042865 | 1.1 | 0.0 | 5.7 | 4.9 | 4.8 | 2.2 | 0.0 | 3.0 | 4.6 | 3.3 | 2.9 | 7.8 | 2.4 | 5.6 | 0.0 |
| *FGT* | GRMZM2G074631 | 5.3 | 7.9 | 32.9 | 16.6 | 14.4 | 8.9 | 43.2 | 16.7 | 19.6 | 4.3 | 38.6 | 37.0 | 8.3 | 25.7 | 4.5 |
| *3GT* | GRMZM2G063550 | **1.1** | **7.9** | **5.7** | **7.8** | **3.8** | **5.5** | **0.9** | **3.0** | **2.3** | **19.6** | **6.7** | **4.5** | **33.3** | **6.4** | **7.9** |
| *3GT* | GRMZM2G159404 | **16.0** | **6.7** | **39.8** | **2.9** | **11.5** | **11.1** | **15.5** | **9.1** | **20.8** | **13.0** | **1.9** | **13.4** | **21.4** | **7.2** | **11.3** |
|  |  |  |  |  |  |  |  |  |  |  |  |  |  |  |  |  |
| **Gene** | **Maize gene ID** | **10.1.M** | **10.2.M** | **10.3.M** | **12.1.M** | **12.2.M** | **12.3.M** | **13.1.M** | **13.2.M** | **13.3.M** | **17.1.M** | **17.2.M** | **17.3.M** | **18.1.M** | **18.2.M** | **18.3.M** |
| *BX8* | GRMZM2G085054 | **661.0** | **716.6** | **1741.0** | **694.2** | **1048.3** | **926.7** | **684.2** | **675.2** | **950.5** | **503.2** | **889.0** | **1645.2** | **633.7** | **682.6** | **997.6** |
| *C4H* | GRMZM2G028677 | **7.9** | **19.4** | **22.0** | **22.2** | **9.7** | **19.1** | **15.6** | **20.7** | **11.8** | **18.6** | **10.5** | **10.4** | **12.4** | **6.9** | **6.5** |
| *4CL* | GRMZM5G805585 | 184.8 | 86.6 | 103.2 | 223.1 | 83.7 | 122.6 | 135.1 | 105.5 | 49.4 | 119.3 | 84.0 | 107.7 | 204.1 | 102.5 | 165.6 |
| *FNSI-2* | GRMZM2G475380 | **5.7** | **10.5** | **4.6** | **3.4** | **13.9** | **10.1** | **4.3** | **18.2** | **15.8** | **0.9** | **8.1** | **7.4** | **8.3** | **6.1** | **6.5** |
| *FLS2* | GRMZM2G069298 | 0.0 | 1.5 | 16.2 | 0.9 | 1.1 | 0.0 | 0.9 | 0.0 | 3.0 | 0.9 | 0.0 | 7.4 | 3.3 | 6.9 | 2.2 |
| *FGT* | GRMZM2G042865 | **6.8** | **10.5** | **39.4** | **4.3** | **9.7** | **7.0** | **6.1** | **11.2** | **24.7** | **5.6** | **8.9** | **9.7** | **9.1** | **7.8** | **4.3** |
| *FGT* | GRMZM2G074631 | **71.4** | **71.7** | **91.6** | **46.2** | **37.6** | **61.3** | **61.5** | **35.4** | **50.3** | **58.7** | **61.4** | **67.6** | **84.3** | **72.1** | **79.6** |
| *3GT* | GRMZM2G063550 | 0.0 | 1.5 | 4.6 | 0.9 | 0.0 | 1.0 | 1.7 | 0.9 | 1.0 | 0.0 | 0.0 | 0.7 | 1.7 | 3.5 | 4.3 |
| *3GT* | GRMZM2G159404 | 6.8 | 3.0 | 7.0 | 4.3 | 7.5 | 7.0 | 3.5 | 6.1 | 9.9 | 1.9 | 7.3 | 7.4 | 4.1 | 9.6 | 6.5 |
|  |  |  |  |  |  |  |  |  |  |  |  |  |  |  |  |  |
| **Gene** | **Maize gene ID** | **20.1.H** | **20.2.H** | **20.3.H** | **26.1.H** | **26.2.H** | **26.3.H** | **27.1.H** | **27.2.H** | **27.3.H** | **29.1.H** | **29.2.H** | **29.3.H** | **30.1.H** | **30.2.H** | **30.3.H** |
| *BX8* | GRMZM2G085054 | 357.8 | 836.0 | 877.4 | 393.0 | 774.0 | 1318.7 | 564.5 | 896.6 | 860.2 | 552.0 | 755.8 | 1163.7 | 611.1 | 478.4 | 1278.7 |
| *C4H* | GRMZM2G028677 | 46.7 | 31.0 | 31.2 | 24.9 | 18.6 | 16.4 | 41.3 | 19.2 | 0.0 | 41.3 | 8.8 | 4.9 | 80.9 | 16.8 | 21.6 |
| *4CL* | GRMZM5G805585 | 237.5 | 75.1 | 71.0 | 162.5 | 102.0 | 77.5 | 178.6 | 117.9 | 141.0 | 164.0 | 23.8 | 81.7 | 55.2 | 166.5 | 116.6 |
| *FNSI-2* | GRMZM2G475380 | 5.2 | 7.5 | 7.0 | 9.1 | 5.9 | 1.8 | 10.1 | 7.7 | 3.9 | 0.0 | 0.9 | 1.9 | 5.3 | 6.3 | 5.9 |
| *FLS2* | GRMZM2G069298 | 11.4 | 5.6 | 8.6 | 9.1 | 9.8 | 19.1 | 2.5 | 19.2 | 34.8 | 2.5 | 10.6 | 11.7 | 26.7 | 1.0 | 20.6 |
| *FGT* | GRMZM2G042865 | 9.3 | 11.3 | 7.0 | 7.5 | 12.8 | 23.7 | 5.1 | 6.7 | 15.4 | 10.0 | 5.3 | 28.2 | 7.1 | 4.2 | 18.6 |
| *FGT* | GRMZM2G074631 | 101.6 | 93.0 | 64.0 | 116.9 | 79.5 | 109.4 | 118.0 | 77.7 | 68.5 | 77.6 | 85.4 | 92.4 | 140.5 | 72.2 | 74.5 |
| *3GT* | GRMZM2G063550 | 3.1 | 0.0 | 0.0 | 0.8 | 3.9 | 0.0 | 3.4 | 0.0 | 1.0 | 2.5 | 0.0 | 1.0 | 0.9 | 0.0 | 0.0 |
| *3GT* | GRMZM2G159404 | 13.5 | 8.5 | 10.9 | 6.6 | 1.0 | 7.3 | 12.6 | 5.8 | 8.7 | 2.5 | 7.0 | 23.4 | 8.0 | 15.7 | 12.7 |

Table S7c. (cont.) Library level normalized counts for midland vs. lowland DE genes involved in phenylpropanoid, flavonoid, and lignin biosynthesis, and nucleotide-sugar interconversion

| **Lignin biosynthesis** | |  |  |  |  |  |  |  |  |  |  |  |  |  |  |  |
| --- | --- | --- | --- | --- | --- | --- | --- | --- | --- | --- | --- | --- | --- | --- | --- | --- |
| **Gene** | **Maize gene ID** | **1.1.L** | **1.2.L** | **1.3.L** | **4.1.L** | **4.2.L** | **4.3.L** | **6.1.L** | **6.2.L** | **6.3.L** | **7.1.L** | **7.2.L** | **7.3.L** | **9.1.L** | **9.2.L** | **9.3.L** |
| *HCT* | GRMZM2G043154 | **97.9** | **41.6** | **18.2** | **33.2** | **15.4** | **21.1** | **16.4** | **28.9** | **42.7** | **43.5** | **26.0** | **23.5** | **7.1** | **74.7** | **7.9** |
| *HCT* | GRMZM2G122503 | **5.3** | **76.5** | **3.4** | **62.5** | **38.5** | **17.7** | **4.3** | **20.5** | **23.1** | **6.5** | **15.4** | **23.5** | **23.8** | **7.2** | **3.4** |
| *HCT* | GRMZM2G177349 | **0.0** | **7.9** | **5.7** | **0.0** | **39.5** | **3.3** | **6.9** | **2.3** | **8.1** | **10.9** | **3.9** | **2.2** | **8.3** | **2.4** | **0.0** |
| *HCT12* | GRMZM2G179703 | 39.4 | 64.1 | 78.4 | 38.1 | 37.5 | 22.2 | 21.6 | 34.2 | 107.3 | 87.0 | 68.5 | 44.8 | 60.6 | 58.6 | 41.8 |
| *CCR1* | GRMZM2G099420 | **30.9** | **58.5** | **13.6** | **19.5** | **13.5** | **4.4** | **18.1** | **6.1** | **1.2** | **13.0** | **22.2** | **3.4** | **21.4** | **18.5** | **10.2** |
| *CCR* | GRMZM2G109720 | **227.7** | **218.1** | **255.6** | **249.2** | **235.8** | **241.6** | **244.4** | **230.5** | **243.5** | **284.9** | **295.0** | **218.4** | **295.9** | **240.8** | **239.5** |
| *CAD* | GRMZM2G090980 | **1006.4** | **899.5** | **610.0** | **891.1** | **871.8** | **760.4** | **709.9** | **785.9** | **660.2** | **744.8** | **733.8** | **651.9** | **956.7** | **627.0** | **657.4** |
| *OMT3* | GRMZM2G077486 | **164.9** | **220.4** | **159.0** | **145.6** | **159.7** | **151.9** | **152.0** | **194.0** | **223.9** | **254.4** | **228.5** | **194.9** | **196.1** | **156.5** | **158.1** |
|  |  |  |  |  |  |  |  |  |  |  |  |  |  |  |  |  |
| **Gene** | **Maize gene ID** | **10.1.M** | **10.2.M** | **10.3.M** | **12.1.M** | **12.2.M** | **12.3.M** | **13.1.M** | **13.2.M** | **13.3.M** | **17.1.M** | **17.2.M** | **17.3.M** | **18.1.M** | **18.2.M** | **18.3.M** |
| *HCT* | GRMZM2G043154 | 28.3 | 13.4 | 8.1 | 23.9 | 21.5 | 17.1 | 15.6 | 24.2 | 18.8 | 30.7 | 7.3 | 1.5 | 30.6 | 6.1 | 12.9 |
| *HCT* | GRMZM2G122503 | 20.4 | 7.5 | 25.5 | 4.3 | 2.1 | 14.1 | 1.7 | 7.8 | 8.9 | 0.0 | 9.7 | 8.9 | 11.6 | 4.3 | 2.2 |
| *HCT* | GRMZM2G177349 | 0.0 | 0.0 | 0.0 | 1.7 | 1.1 | 1.0 | 0.9 | 0.0 | 1.0 | 0.0 | 0.0 | 6.7 | 2.5 | 3.5 | 0.0 |
| *HCT12* | GRMZM2G179703 | **123.6** | **70.2** | **42.9** | **107.7** | **65.5** | **92.5** | **55.4** | **72.6** | **94.8** | **81.1** | **46.8** | **86.9** | **124.8** | **64.3** | **109.7** |
| *CCR1* | GRMZM2G099420 | 12.5 | 1.5 | 0.0 | 2.6 | 4.3 | 1.0 | 39.8 | 0.9 | 5.9 | 0.0 | 4.0 | 1.5 | 14.0 | 1.7 | 0.0 |
| *CCR* | GRMZM2G109720 | 236.9 | 146.3 | 182.0 | 217.1 | 251.1 | 228.2 | 257.2 | 147.0 | 246.8 | 217.1 | 156.6 | 265.9 | 233.8 | 197.1 | 225.8 |
| *CAD* | GRMZM2G090980 | 755.1 | 574.8 | 533.2 | 817.3 | 416.3 | 499.6 | 653.0 | 475.5 | 576.4 | 904.8 | 616.1 | 814.8 | 802.3 | 513.3 | 619.2 |
| *OMT3* | GRMZM2G077486 | 209.7 | 165.7 | 109.0 | 100.9 | 150.2 | 152.8 | 215.6 | 111.5 | 117.5 | 209.7 | 147.8 | 133.0 | 123.9 | 112.0 | 116.1 |
|  |  |  |  |  |  |  |  |  |  |  |  |  |  |  |  |  |
| **Gene** | **Maize gene ID** | **20.1.H** | **20.2.H** | **20.3.H** | **26.1.H** | **26.2.H** | **26.3.H** | **27.1.H** | **27.2.H** | **27.3.H** | **29.1.H** | **29.2.H** | **29.3.H** | **30.1.H** | **30.2.H** | **30.3.H** |
| *HCT* | GRMZM2G043154 | 7.3 | 12.2 | 11.7 | 10.8 | 20.6 | 26.4 | 31.2 | 14.4 | 21.2 | 115.2 | 16.7 | 18.5 | 16.9 | 18.8 | 2.9 |
| *HCT* | GRMZM2G122503 | 4.1 | 5.6 | 6.2 | 2.5 | 4.9 | 2.7 | 2.5 | 1.0 | 5.8 | 7.5 | 2.6 | 3.9 | 2.7 | 1.0 | 0.0 |
| *HCT* | GRMZM2G177349 | 0.0 | 1.9 | 3.9 | 21.6 | 1.0 | 1.8 | 21.1 | 0.0 | 1.9 | 0.0 | 1.8 | 3.9 | 0.0 | 0.0 | 2.9 |
| *HCT12* | GRMZM2G179703 | 60.1 | 88.3 | 63.2 | 150.1 | 160.9 | 112.1 | 76.7 | 101.6 | 105.2 | 63.8 | 58.1 | 67.1 | 117.4 | 52.3 | 36.3 |
| *CCR1* | GRMZM2G099420 | 4.1 | 0.0 | 0.8 | 24.9 | 17.7 | 9.1 | 21.9 | 10.5 | 1.0 | 6.3 | 15.9 | 3.9 | 3.6 | 9.4 | 2.9 |
| *CCR* | GRMZM2G109720 | 260.3 | 234.8 | 178.8 | 227.2 | 251.1 | 232.4 | 244.3 | 241.6 | 196.9 | 220.3 | 255.4 | 207.3 | 212.6 | 218.8 | 238.1 |
| *CAD* | GRMZM2G090980 | 688.6 | 587.1 | 512.1 | 774.4 | 628.8 | 525.9 | 996.8 | 604.1 | 611.1 | 1056.5 | 609.5 | 631.5 | 564.0 | 550.7 | 487.0 |
| *OMT3* | GRMZM2G077486 | 101.6 | 124.9 | 143.6 | 119.4 | 72.6 | 108.5 | 84.3 | 148.6 | 107.2 | 130.2 | 117.2 | 104.1 | 173.5 | 94.2 | 101.9 |
|  |  |  |  |  |  |  |  |  |  |  |  |  |  |  |  |  |
|  | | | | |  |  |  |  |  |  |  |  |  |  |  |  |
| **Plant nucleotide-sugar interconversion enzymes** | | | | | | |  |  |  |  |  |  |  |  |  |  |
| **Gene** | **Maize gene ID** | **1.1.L** | **1.2.L** | **1.3.L** | **4.1.L** | **4.2.L** | **4.3.L** | **6.1.L** | **6.2.L** | **6.3.L** | **7.1.L** | **7.2.L** | **7.3.L** | **9.1.L** | **9.2.L** | **9.3.L** |
| *RHM3* | GRMZM2G044281 | 125.5 | 154.0 | 203.3 | 129.9 | 90.5 | 121.9 | 120.9 | 139.2 | 133.9 | 203.3 | 99.3 | 103.1 | 124.8 | 91.5 | 106.2 |
|  | | | | | | | | | | | | | | | | |
| Table S7c. (cont.) Library level normalized counts for midland vs. lowland DE genes involved in phenylpropanoid, flavonoid, and lignin biosynthesis, and nucleotide-sugar interconversion | | | | | | | | | | | | | | | | |
|  |  |  |  |  |  |  |  |  |  |  |  |  |  |  |  |  |
| **Gene** | **Maize gene ID** | **10.1.M** | **10.2.M** | **10.3.M** | **12.1.M** | **12.2.M** | **12.3.M** | **13.1.M** | **13.2.M** | **13.3.M** | **17.1.M** | **17.2.M** | **17.3.M** | **18.1.M** | **18.2.M** | **18.3.M** |
| *RHM3* | GRMZM2G044281 | **155.3** | **97.0** | **149.5** | **164.1** | **200.6** | **166.9** | **175.8** | **159.9** | **165.8** | **167.7** | **214.0** | **184.2** | **195.8** | **158.9** | **236.5** |
|  |  |  |  |  |  |  |  |  |  |  |  |  |  |  |  |  |
| **Gene** | **Maize gene ID** | **20.1.H** | **20.2.H** | **20.3.H** | **26.1.H** | **26.2.H** | **26.3.H** | **27.1.H** | **27.2.H** | **27.3.H** | **29.1.H** | **29.2.H** | **29.3.H** | **30.1.H** | **30.2.H** | **30.3.H** |
| *RHM3* | GRMZM2G044281 | 216.7 | 121.2 | 99.1 | 119.4 | 72.6 | 43.7 | 137.3 | 94.9 | 73.4 | 152.7 | 82.8 | 80.8 | 96.1 | 95.3 | 82.3 |

Note. Normalized counts for genes encoding enzymes involved in phenylpropanoid flavonoid, and lignin biosynthesis, and plant nucleotide-sugar interconversion were output from DESeq2 for all pairwise comparisons of elevation of landrace origin: a) highland vs. lowland; b) highland vs. midland; and c) midland vs. lowland. Libraries are represented by a population.sample.elevation format (i.e. 1.1.L = population one sample one from the lowlands). For each gene, we made normalized counts bold for the elevation exhibiting the highest expression value.

Figure S1. Experimental planting design – modified split-plot

| ****** | ****** | ****** | ****** | ****** | ****** | ****** | ****** | ****** | ****** | ****** | ****** | ****** | ****** | ****** | ****** | ****** | ****** | ****** | ****** | ****** |
| --- | --- | --- | --- | --- | --- | --- | --- | --- | --- | --- | --- | --- | --- | --- | --- | --- | --- | --- | --- | --- |
| **Block 1** | **1** | **2** | **4** | **5** | **3** | ****** | ****** | **10** | **12** | **13** | **18** | **17** | ****** | ****** | **20** | **27** | **26** | **30** | **29** | ****** |
| ****** | **1** | **2** | **4** | **5** | **3** | ****** | ****** | **10** | **12** | **13** | **18** | **17** | ****** | ****** | **20** | **27** | **26** | **30** | **29** | ****** |
| ****** | **1** | **2** | **4** | **5** | **3** | ****** | ****** | **10** | **12** | **13** | **18** | **17** | ****** | ****** | **20** | **27** | **26** | **30** | **29** | ****** |
| ****** | **1** | **2** | **4** | **5** | **3** | ****** | ****** | **10** | **12** | **13** | **18** | **17** | ****** | ****** | **20** | **27** | **26** | **30** | **29** | ****** |
| ****** | **1** | **2** | **4** | **5** | **3** | ****** | ****** | **10** | **12** | **13** | **18** | **17** | ****** | ****** | **20** | **27** | **26** | **30** | **29** | ****** |
| ****** | **1** | **2** | **4** | **5** | **3** | ****** | ****** | **10** | **12** | **13** | **18** | **17** | ****** | ****** | **20** | **27** | **26** | **30** | **29** | ****** |
| ****** | **1** | **2** | **4** | **5** | **3** | ****** | ****** | **10** | **12** | **13** | **18** | **17** | ****** | ****** | **20** | **27** | **26** | **30** | **29** | ****** |
| ****** | **1** | **2** | **4** | **5** | **3** | ****** | ****** | **10** | **12** | **13** | **18** | **17** | ****** | ****** | **20** | **27** | **26** | **30** | **29** | ****** |
| ****** | **1** | **2** | **4** | **5** | **3** | ****** | ****** | **10** | **12** | **13** | **18** | **17** | ****** | ****** | **20** | **27** | **26** | **30** | **29** | ****** |
| ****** | **1** | **2** | **4** | **5** | **3** | ****** | ****** | **10** | **12** | **13** | **18** | **17** | ****** | ****** | **20** | **27** | **26** | **30** | **29** | ****** |
| ****** | ****** | ****** | ****** | ****** | ****** | ****** | ****** | ****** | ****** | ****** | ****** | ****** | ****** | ****** | ****** | ****** | ****** | ****** | ****** | ****** |
|  |  |  |  |  |  |  |  |  |  |  |  |  |  |  |  |  |  |  |  |  |
| ****** | ****** | ****** | ****** | ****** | ****** | ****** | ****** | ****** | ****** | ****** | ****** | ****** | ****** | ****** | ****** | ****** | ****** | ****** | ****** | ****** |
| **Block 2** | **18** | **17** | **12** | **10** | **13** | ****** | ****** | **30** | **29** | **20** | **26** | **27** | ****** | ****** | **1** | **2** | **4** | **3** | **5** | ****** |
| ****** | **18** | **17** | **12** | **10** | **13** | ****** | ****** | **30** | **29** | **20** | **26** | **27** | ****** | ****** | **1** | **2** | **4** | **3** | **5** | ****** |
| ****** | **18** | **17** | **12** | **10** | **13** | ****** | ****** | **30** | **29** | **20** | **26** | **27** | ****** | ****** | **1** | **2** | **4** | **3** | **5** | ****** |
| ****** | **18** | **17** | **12** | **10** | **13** | ****** | ****** | **30** | **29** | **20** | **26** | **27** | ****** | ****** | **1** | **2** | **4** | **3** | **5** | ****** |
| ****** | **18** | **17** | **12** | **10** | **13** | ****** | ****** | **30** | **29** | **20** | **26** | **27** | ****** | ****** | **1** | **2** | **4** | **3** | **5** | ****** |
| ****** | **18** | **17** | **12** | **10** | **13** | ****** | ****** | **30** | **29** | **20** | **26** | **27** | ****** | ****** | **1** | **2** | **4** | **3** | **5** | ****** |
| ****** | **18** | **17** | **12** | **10** | **13** | ****** | ****** | **30** | **29** | **20** | **26** | **27** | ****** | ****** | **1** | **2** | **4** | **3** | **5** | ****** |
| ****** | **18** | **17** | **12** | **10** | **13** | ****** | ****** | **30** | **29** | **20** | **26** | **27** | ****** | ****** | **1** | **2** | **4** | **3** | **5** | ****** |
| ****** | **18** | **17** | **12** | **10** | **13** | ****** | ****** | **30** | **29** | **20** | **26** | **27** | ****** | ****** | **1** | **2** | **4** | **3** | **5** | ****** |
| ****** | **18** | **17** | **12** | **10** | **13** | ****** | ****** | **30** | **29** | **20** | **26** | **27** | ****** | ****** | **1** | **2** | **4** | **3** | **5** | ****** |
| ****** | ****** | ****** | ****** | ****** | ****** | ****** | ****** | ****** | ****** | ****** | ****** | ****** | ****** | ****** | ****** | ****** | ****** | ****** | ****** | ****** |
|  |  |  |  |  |  |  |  |  |  |  |  |  |  |  |  |  |  |  |  |  |
| ****** | ****** | ****** | ****** | ****** | ****** | ****** | ****** | ****** | ****** | ****** | ****** | ****** | ****** | ****** | ****** | ****** | ****** | ****** | ****** | ****** |
| **Block 3** | **20** | **26** | **29** | **30** | **27** | ****** | ****** | **4** | **1** | **5** | **2** | **3** | ****** | ****** | **12** | **10** | **13** | **17** | **18** | ****** |
| ****** | **20** | **26** | **29** | **30** | **27** | ****** | ****** | **4** | **1** | **5** | **2** | **3** | ****** | ****** | **12** | **10** | **13** | **17** | **18** | ****** |
| ****** | **20** | **26** | **29** | **30** | **27** | ****** | ****** | **4** | **1** | **5** | **2** | **3** | ****** | ****** | **12** | **10** | **13** | **17** | **18** | ****** |
| ****** | **20** | **26** | **29** | **30** | **27** | ****** | ****** | **4** | **1** | **5** | **2** | **3** | ****** | ****** | **12** | **10** | **13** | **17** | **18** | ****** |
| ****** | **20** | **26** | **29** | **30** | **27** | ****** | ****** | **4** | **1** | **5** | **2** | **3** | ****** | ****** | **12** | **10** | **13** | **17** | **18** | ****** |
| ****** | **20** | **26** | **29** | **30** | **27** | ****** | ****** | **4** | **1** | **5** | **2** | **3** | ****** | ****** | **12** | **10** | **13** | **17** | **18** | ****** |
| ****** | **20** | **26** | **29** | **30** | **27** | ****** | ****** | **4** | **1** | **5** | **2** | **3** | ****** | ****** | **12** | **10** | **13** | **17** | **18** | ****** |
| ****** | **20** | **26** | **29** | **30** | **27** | ****** | ****** | **4** | **1** | **5** | **2** | **3** | ****** | ****** | **12** | **10** | **13** | **17** | **18** | ****** |
| ****** | **20** | **26** | **29** | **30** | **27** | ****** | ****** | **4** | **1** | **5** | **2** | **3** | ****** | ****** | **12** | **10** | **13** | **17** | **18** | ****** |
| ****** | **20** | **26** | **29** | **30** | **27** | ****** | ****** | **4** | **1** | **5** | **2** | **3** | ****** | ****** | **12** | **10** | **13** | **17** | **18** | ****** |
| ****** | ****** | ****** | ****** | ****** | ****** | ****** | ****** | ****** | ****** | ****** | ****** | ****** | ****** | ****** | ****** | ****** | ****** | ****** | ****** | ****** |

Note. Blue are highland landraces, green are midland landraces, and red are lowland landraces—main plots. Landrace number corresponds with the numbering in Table 1—randomized subplots. Each colored/ numbered square is a *mata* and each light green square containing two asterisks are boarder plants. Three blocks are represented.

Figure S2. Highland and midland landrace relative expression levels for the phenylpropanoid, flavonoid, lignin, and nucleotide-sugar interconversion genes differentially expressed in the midland common garden experiment


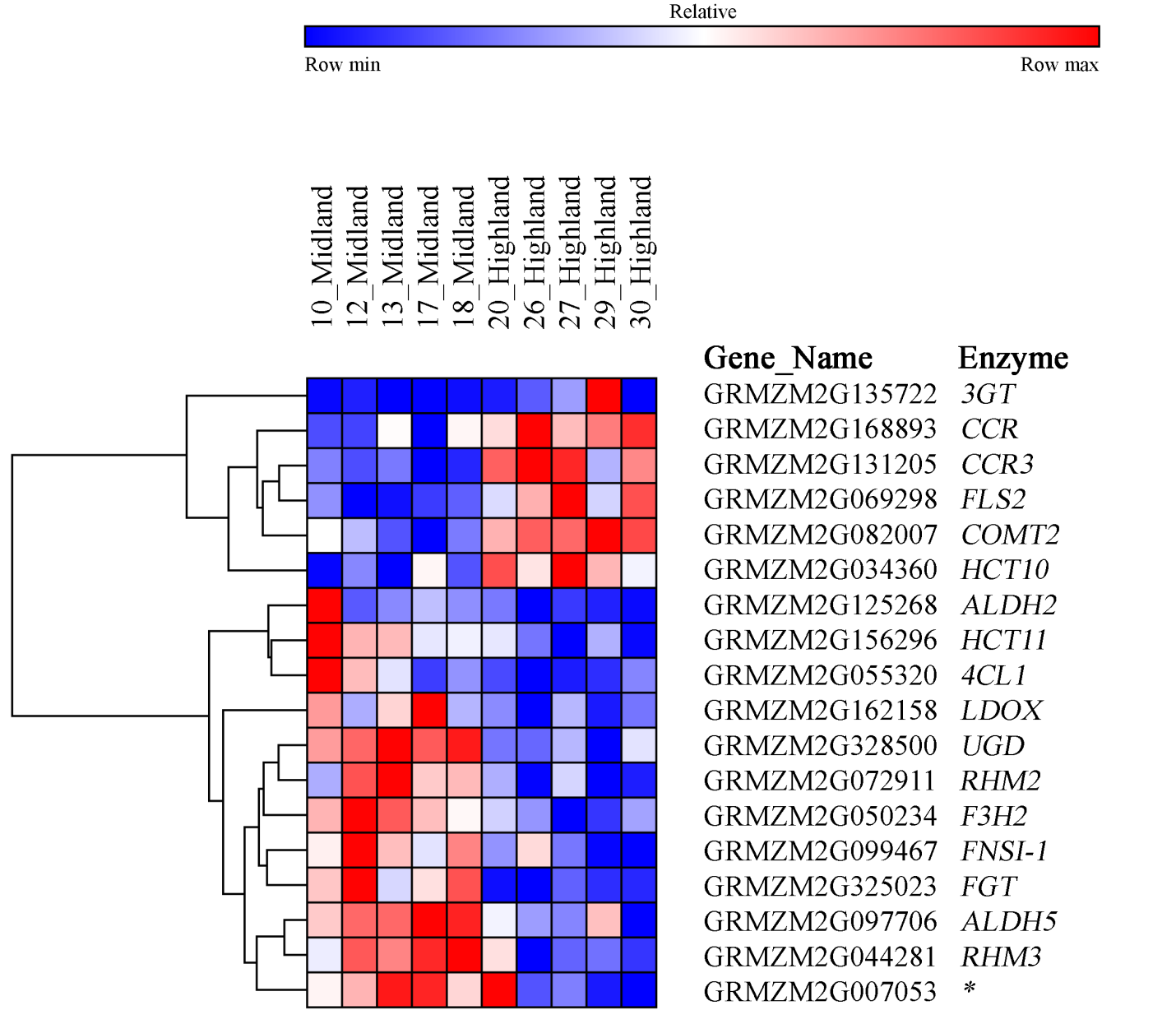


Note. Normalized counts of the phenylpropanoid, flavonoid, lignin, and nucleotide-sugar interconversion genes identified as being differentially expressed between highland and midland landrace populations grown in a midland common garden. We called genes differentially expressed when they exhibited a FDR of 0.05 (Benjamini-Hochberg). Relative expression was determined for each row with red representing the population with the highest expression and blue representing the population with the lowest expression. Each column corresponds to a maize landrace population and each row represents a differentially expressed gene. Gene name (Gene_Name) and enzyme gene names (Enzyme) are provided for the latter. * represents a putative flavonoid:NAD(P)+ oxidoreductase.

Figure S3. Midland and lowland landrace relative expression levels for the phenylpropanoid, flavonoid, lignin, and nucleotide-sugar interconversion genes differentially expressed in our midland common garden experiment


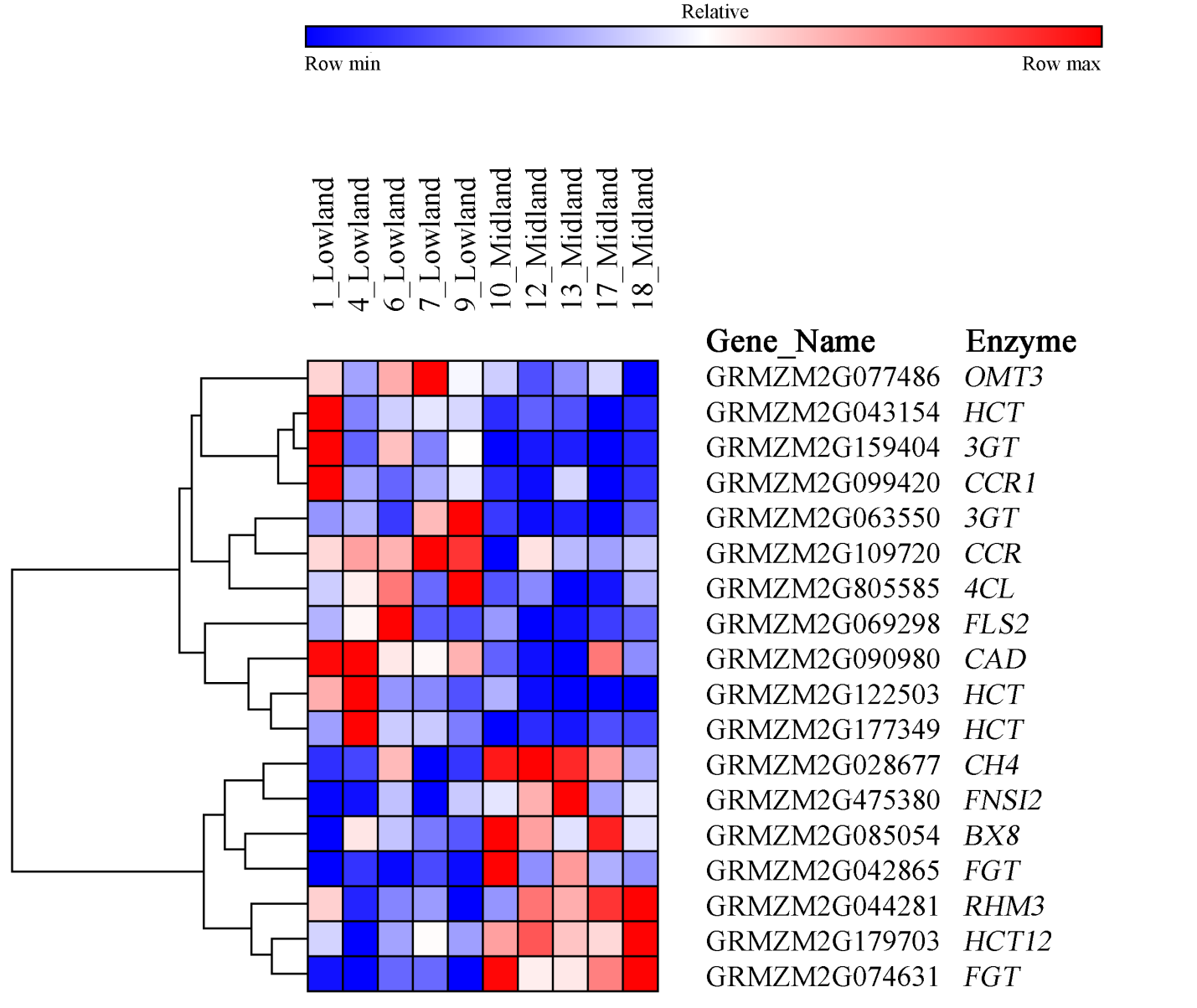


Note. Normalized counts of phenylpropanoid, flavonoid, lignin, and nucleotide-sugar interconversion genes identified as being differentially expressed between midland and lowland landraces grown in our midland common garden. Genes exhibited a FDR of 0.05 (Benjamini-Hochberg) were called differentially expressed. Relative expression was determined for each row with red representing the population with the highest expression and blue representing the population with the lowest expression. Gene name (Gene_Name) and enzyme gene names (Enzyme) are provided for the latter

Figure S4. Distribution of average gene expression values for phenolic candidate genes and the full maize genome across all maize landraces

A.


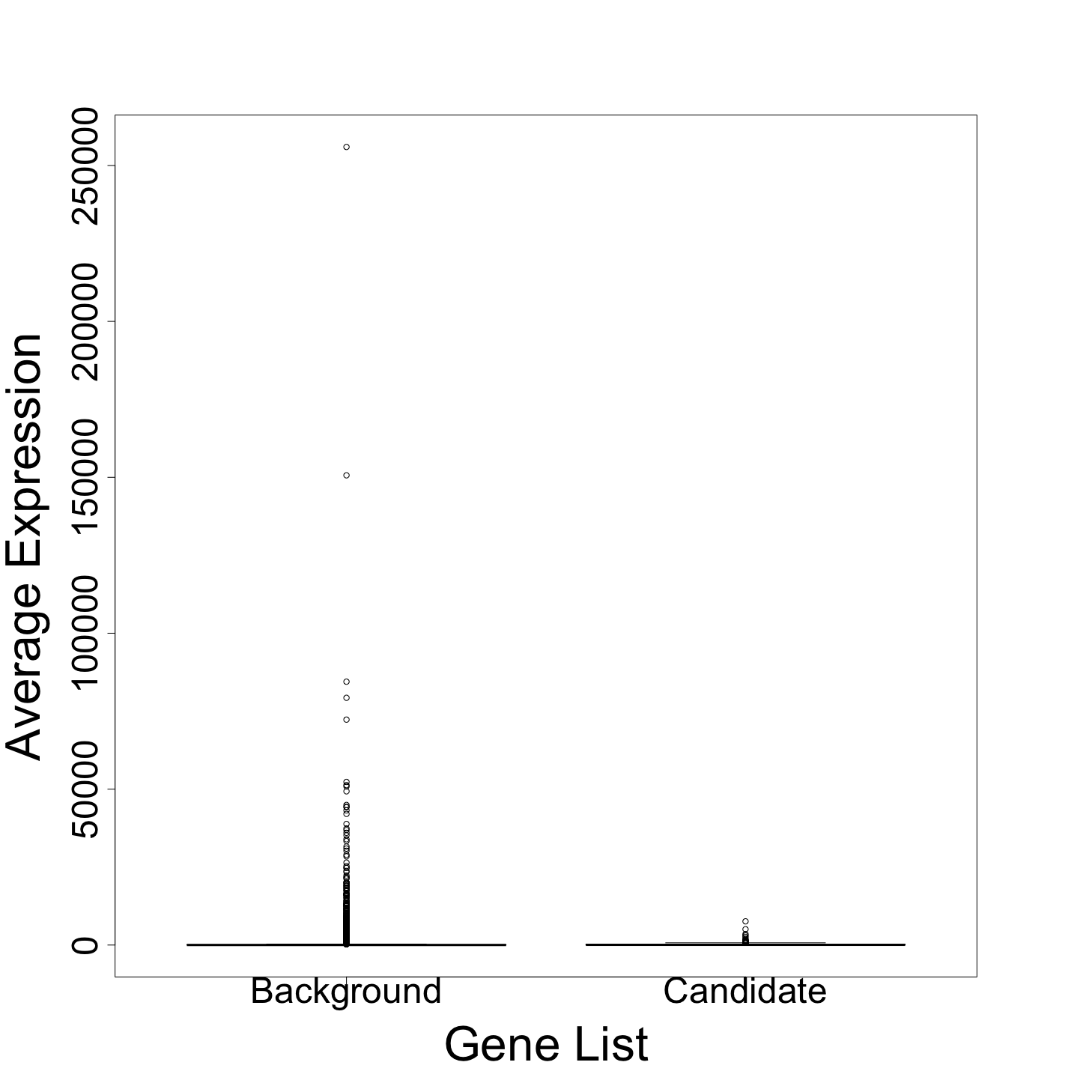


B.


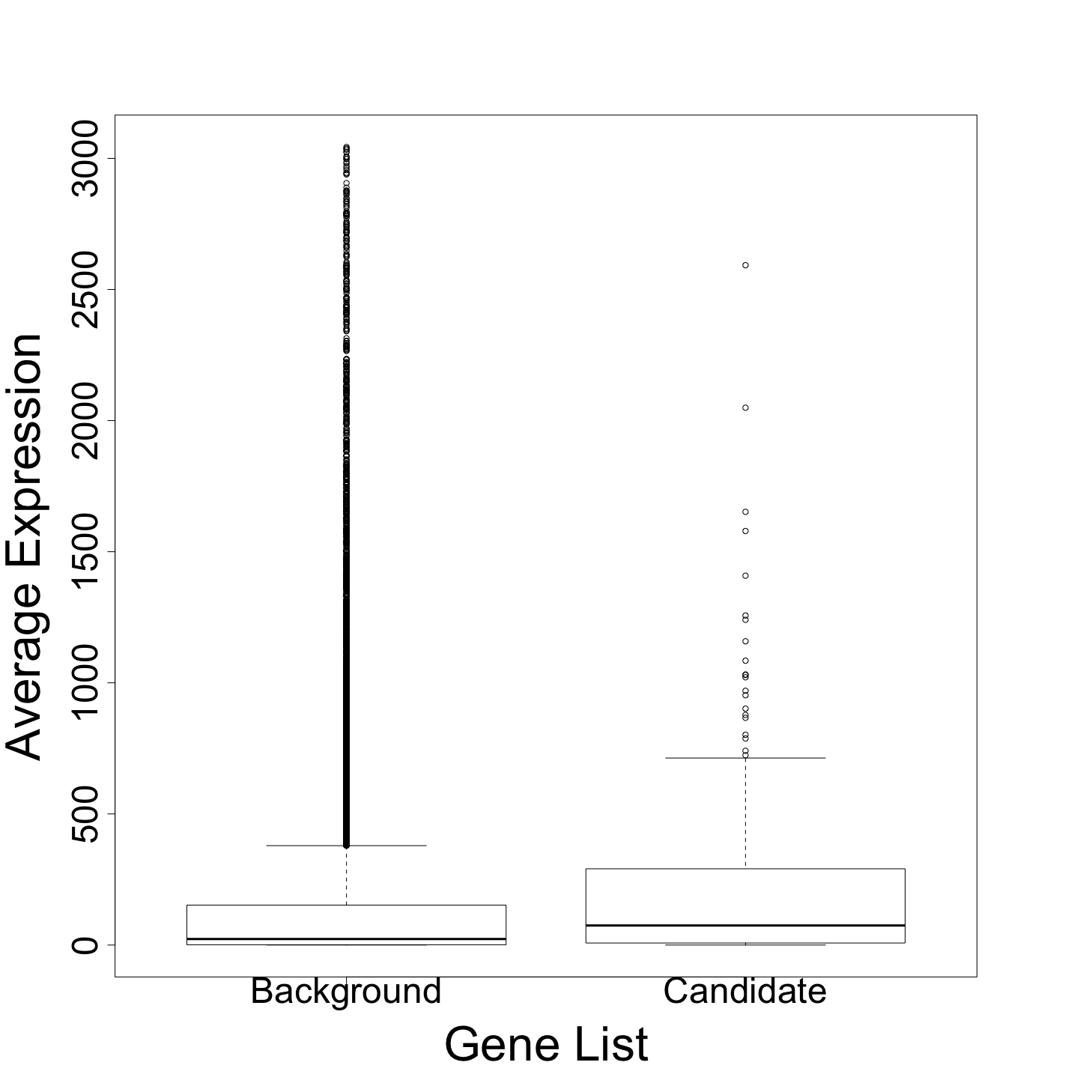


Note. Boxplots displaying average normalized gene expression values for phenolic candidate genes (Candidate) and the full maize genome (Background) before (A) and after (B) outlier removal. The average gene expression values were derived from taking the averages of each loci across all landraces. The Background before outlier removal (A) consisted of 396565 loci and the Candidate consisted of 190 loci. Viewed after we removed all genes with zero expression and with values of expression over 3000 counts (B). This made the Candidate list consist of 175 loci and the Background consist of 33813 loci.

Figure S5. Normalization of leaf background gene set distribution through subsampling

A. Highland::Lowland Before


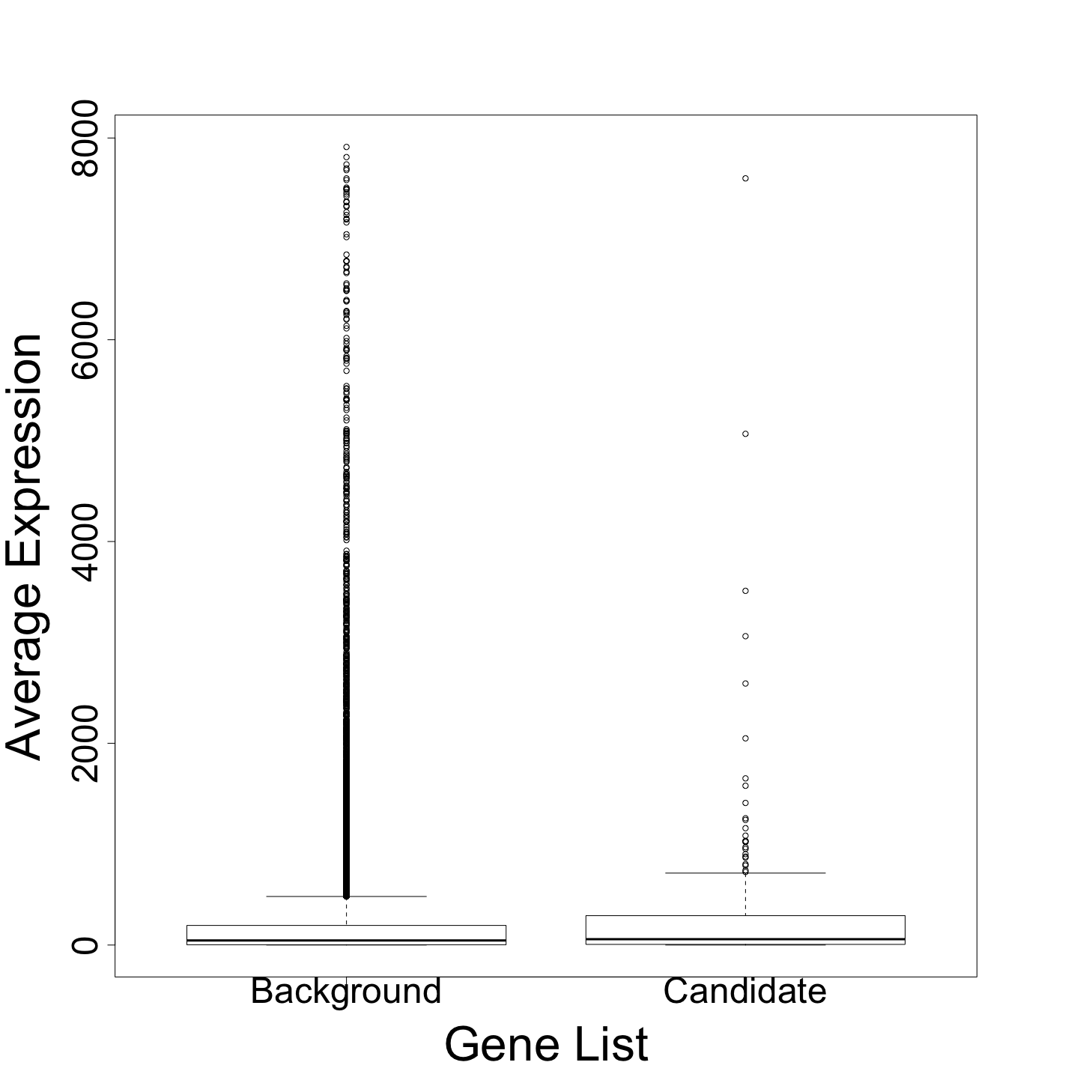


B. Highland::Lowland After


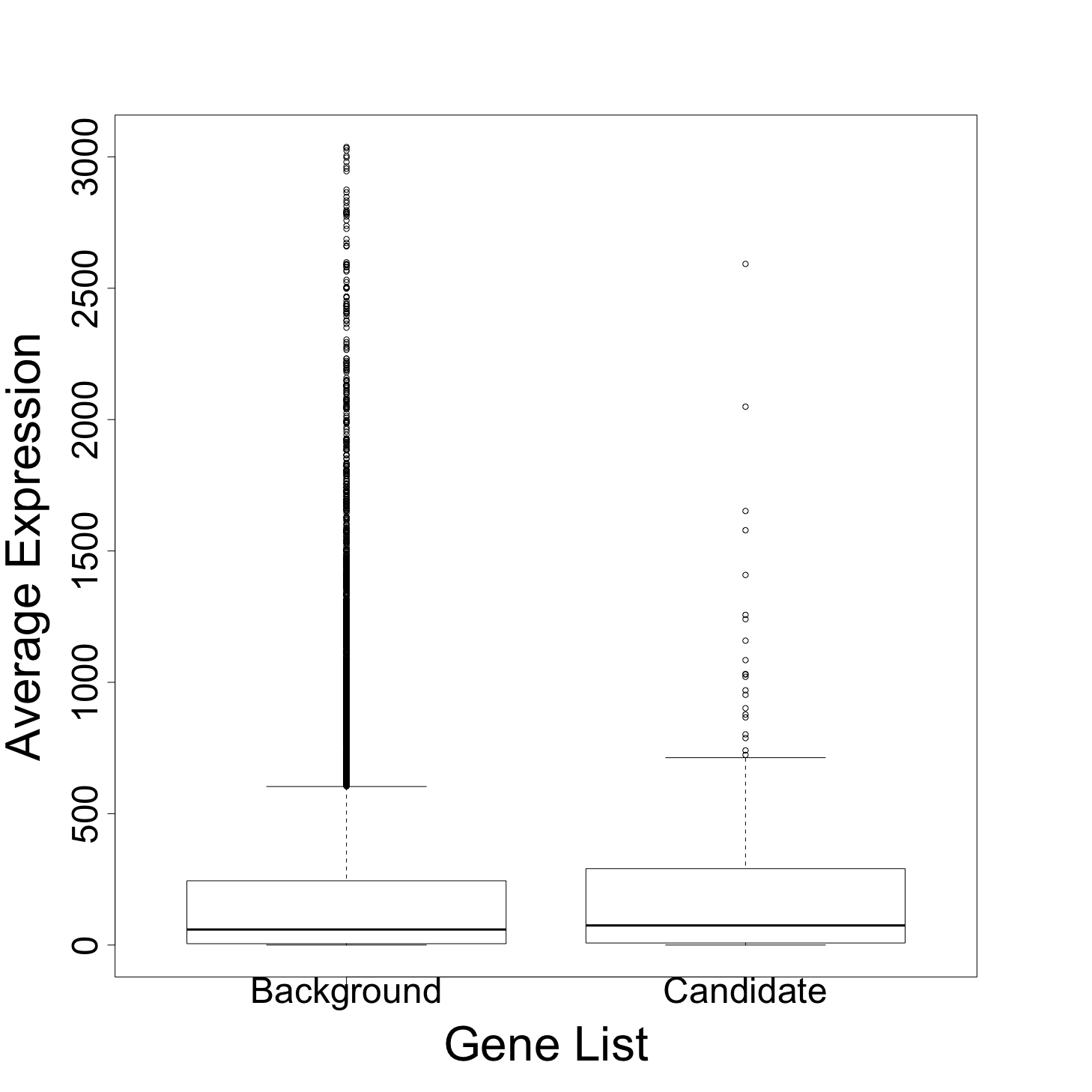


Figure S5. (cont.) Normalization of leaf background gene set distribution through subsampling

C. Highland::Midland Before


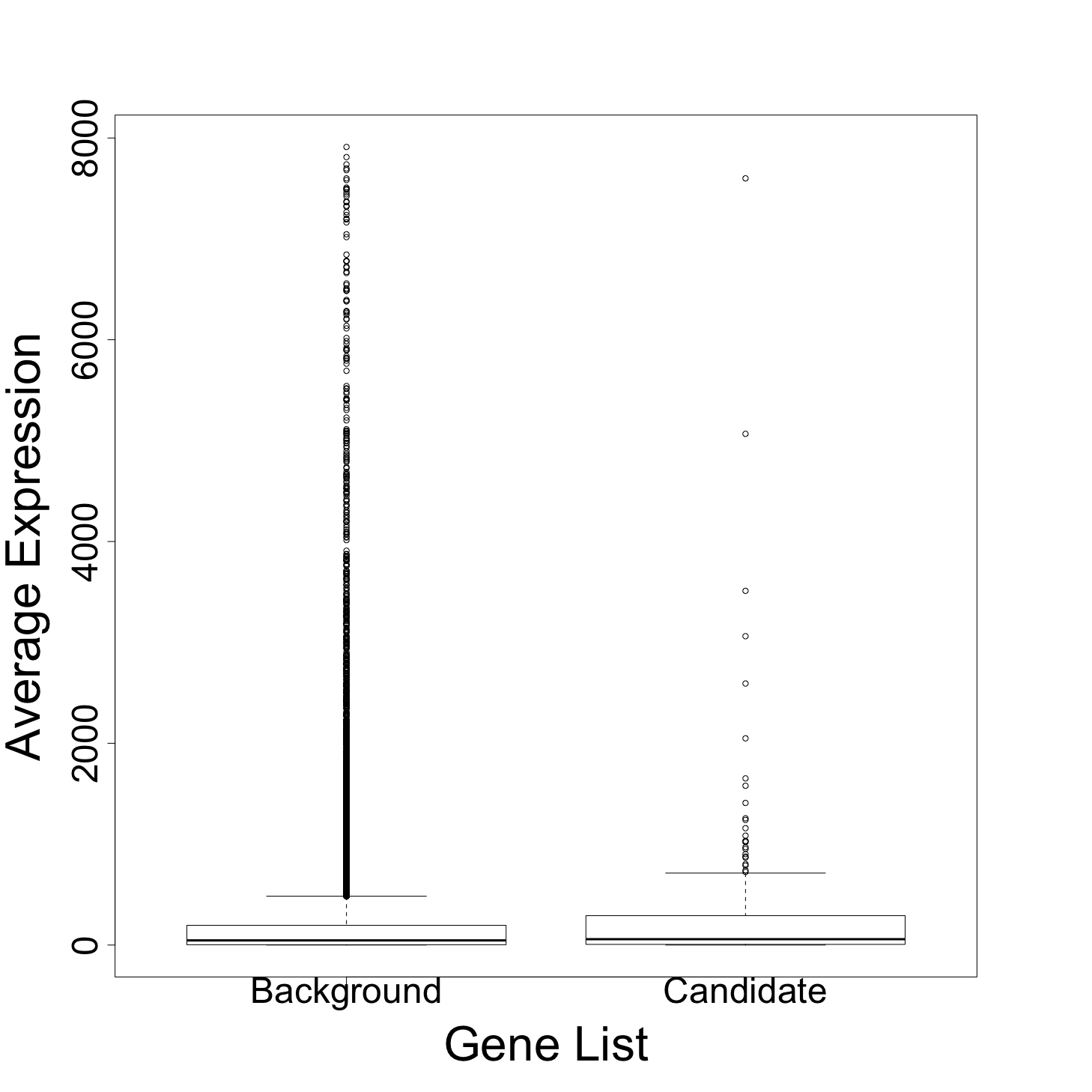


D. Highland::Midland After


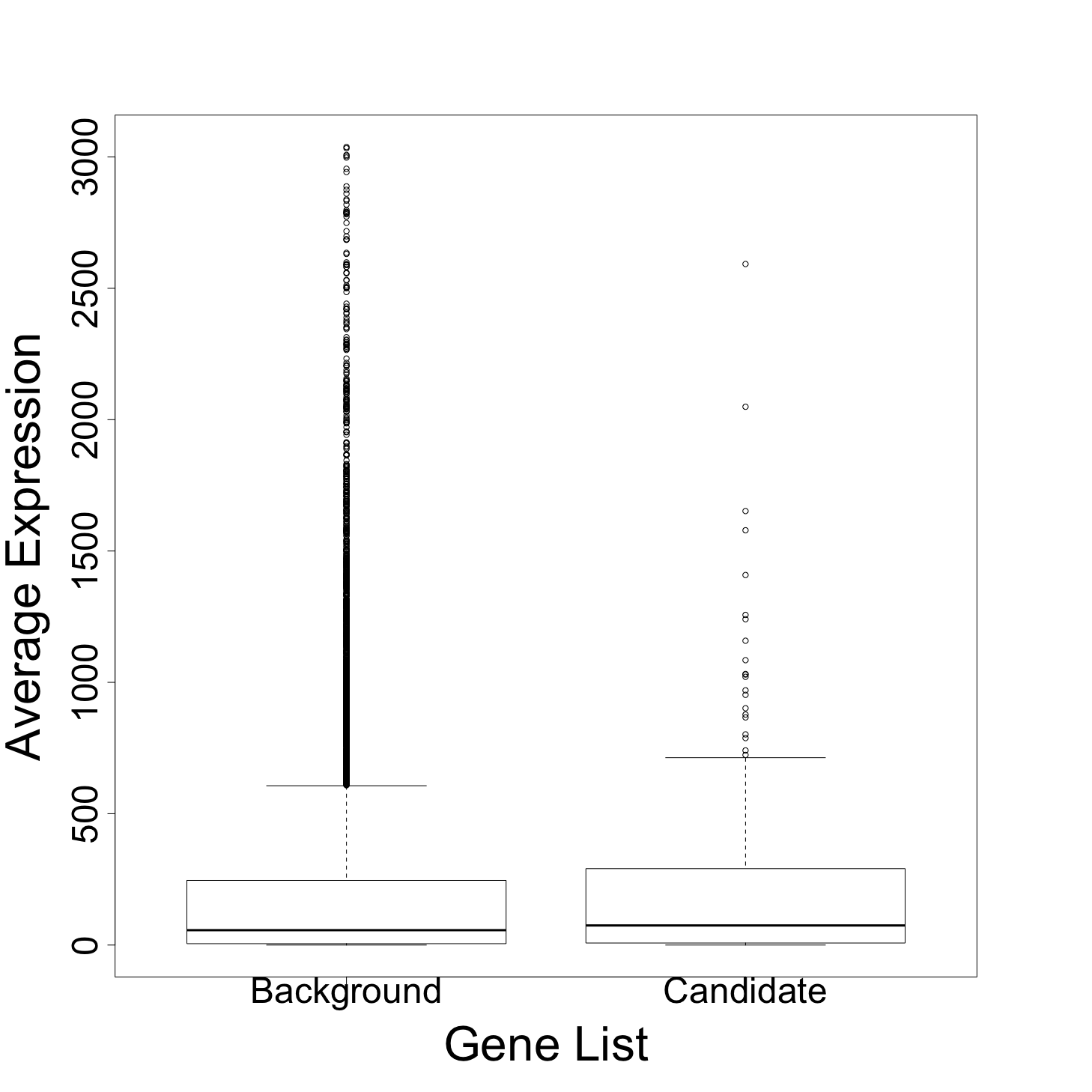


Figure S5. (cont.) Normalization of leaf background gene set distribution through subsampling

E. Midland::Lowland Before


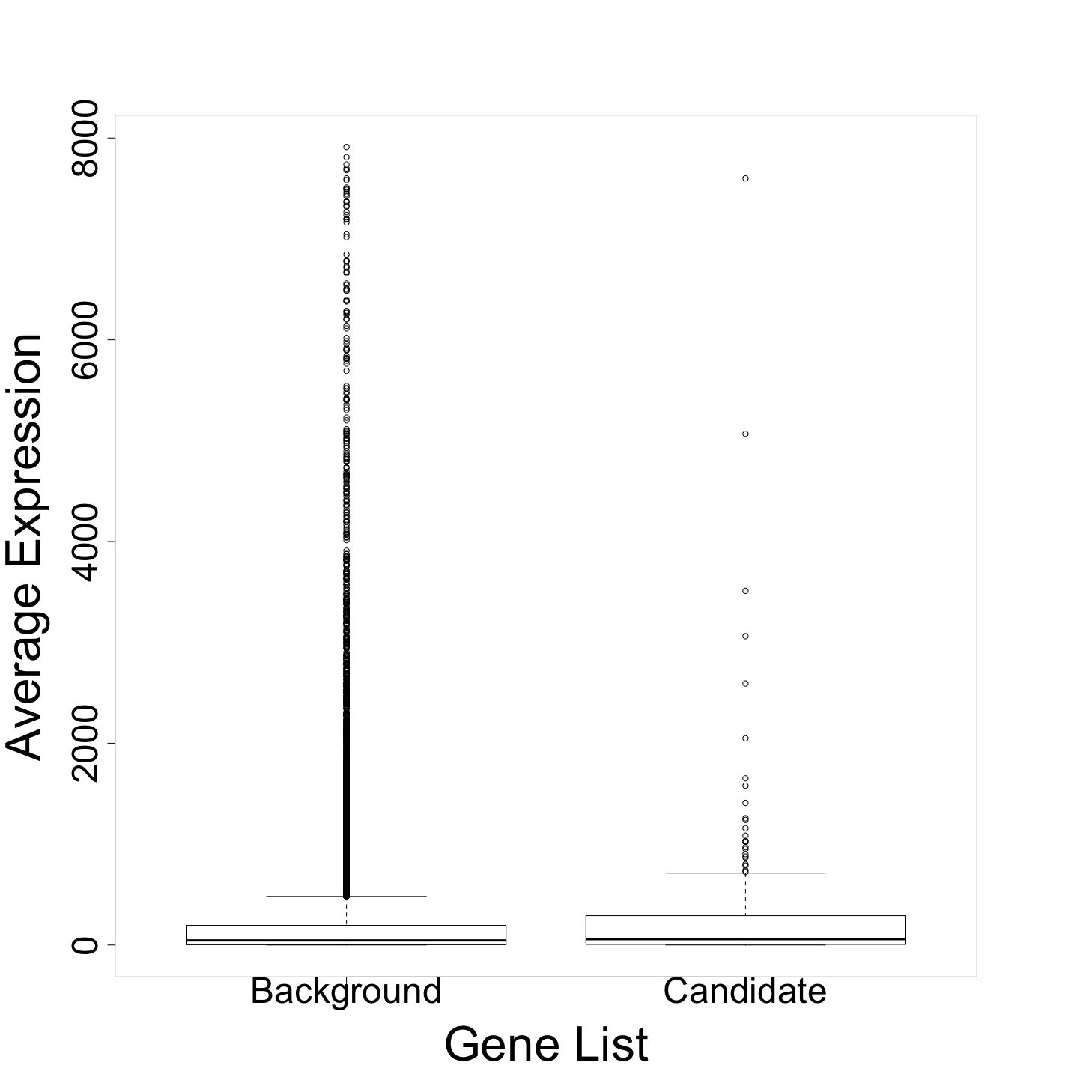


F. Midland::Lowland After


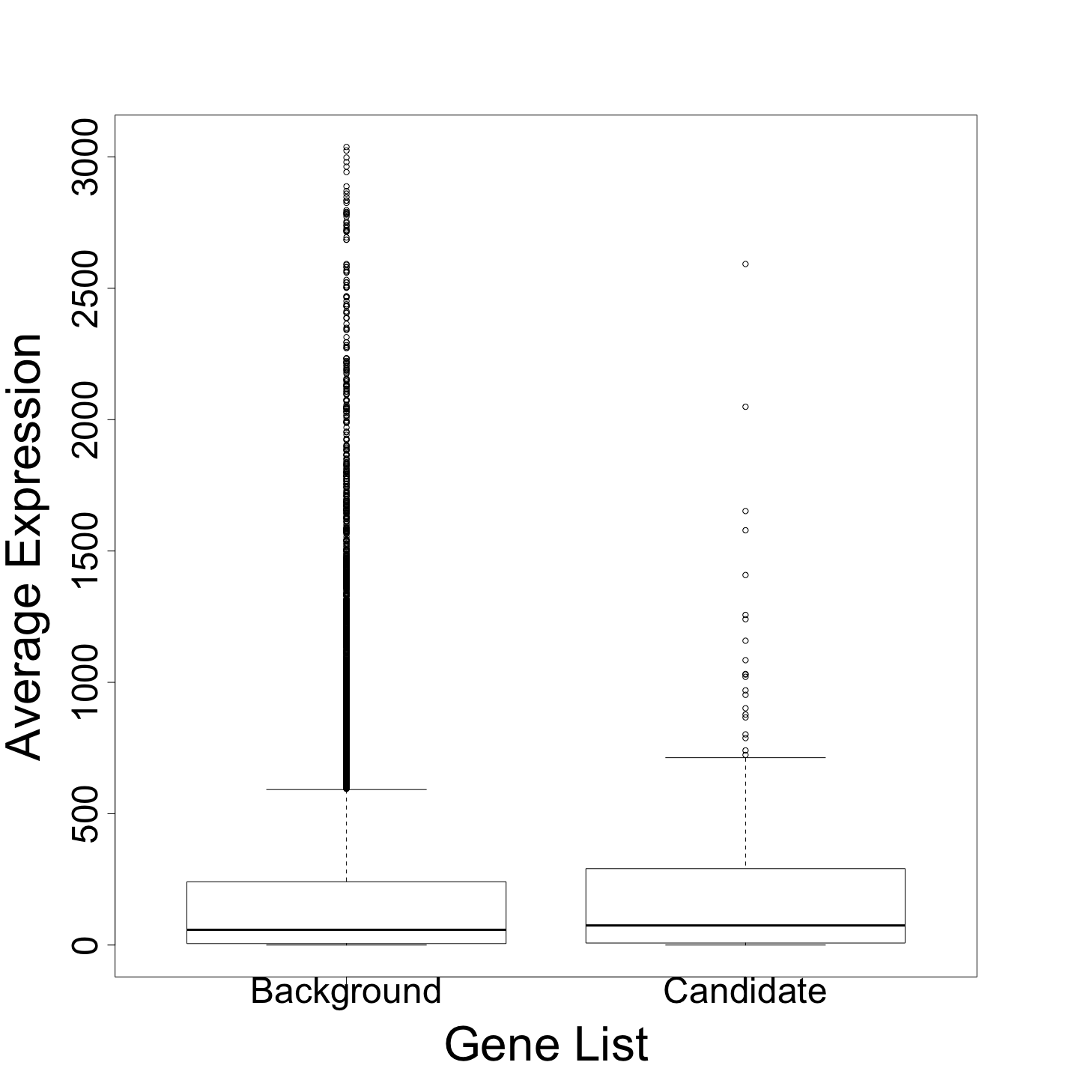


Note. Boxplots displaying the normalization of the leaf background gene set (Background) distribution to that of the phenolic candidate gene set (Candidate) through subsampling. Comparison of the distributions of the counts for the Background and Candidate gene lists before normalization for the highland to lowland (A; Candidate n=190, Background n=28748), highland to midland (C; Candidate n=190, Background n=28562), and midland to lowland (E; Candidate n=190, Background n=28644) comparisons are shown. The upper limits of these three comparisons were set at 8000 counts to clarify the distributions, which led to 119 loci being removed from each pairwise comparison. Comparison of the distributions of the counts for the Background and Candidate gene lists after normalization for the highland to lowland (B; Candidate n=175, Background n=11656), highland to midland (D; Candidate n=175, Background n=11656), and midland to lowland (F; Candidate n=175, Background n=11656) comparisons. Average Expression values are the average counts of each locus across all landraces.

Figure S6. Differentially expressed genes between highland and midland maize landraces encoding enzymes involved in general phenylpropanoid biosynthesis


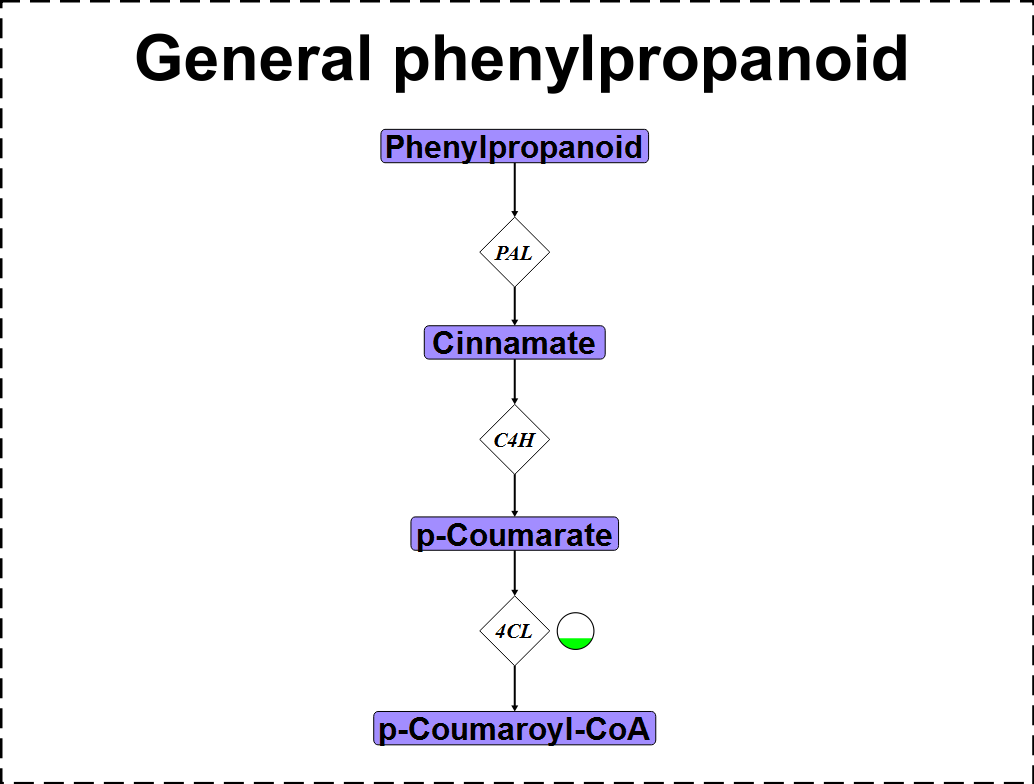


Note. The general phenylpropanoid biosynthesis pathway consisting of three enzymatic steps (white diamonds) and four metabolic intermediates (light purple rectangles). Differentially expressed genes encoding enzymes involved in the biosynthetic pathways are represented to the right or below the enzyme it encodes with a circle filled with either blue or green. Blue represents genes highly expressed in highland landraces while green means the gene was highly expressed in the midland landraces. The more full a circle the higher the log2fold change values. Since the greatest log2fold change observed in this study was 2.11 (an HCT gene identified in the highland vs. lowland comparison), we divided the log2fold change values for each differentially expressed gene by 2.11 to obtain a percentage. A completely full circle represents a log2fold change value of 2.11 while a half full circle represents a log2fold change of 1.055. All differentially expressed genes were significant at a 0.05 FDR rate using a Benjamini-Hochberg correction.

Figure S7. Differentially expressed genes between highland and midland maize landraces encoding enzymes involved in flavonoid biosynthesis


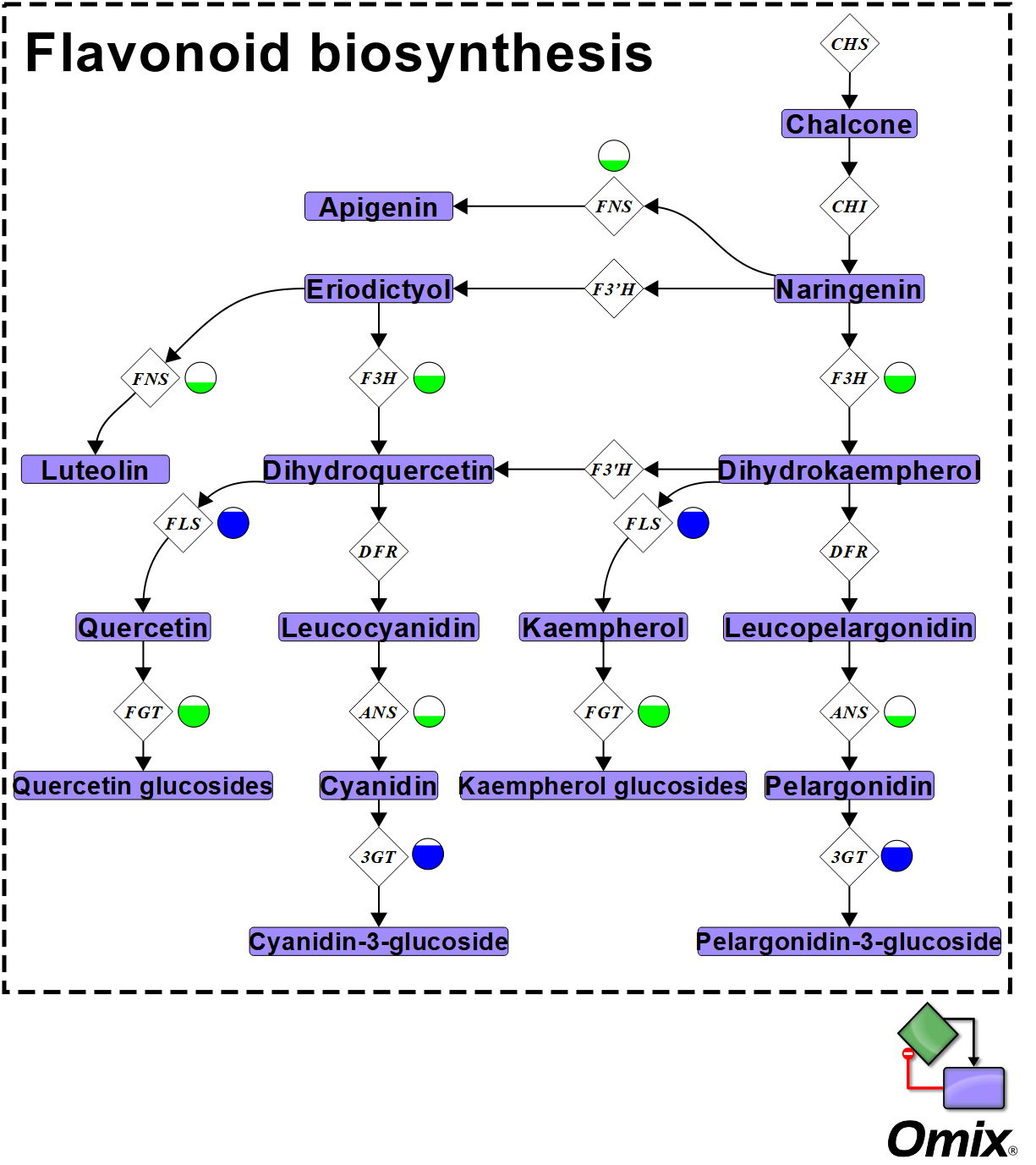


Note. The flavonoid biosynthesis pathway for maize including enzymatic steps for anthocyanin, flavone, and flavonol biosynthesis. Genes up-regulated in highland landraces are represented by blue and those up-regulated in midland landraces are represented by green. Full circles represent a log2fold change of 2.11 while half full circles represent log2fold change of 1.055. Multiple circles next to an enzyme represent multiple genes encoding that enzyme. The ANS circle is the expression level of the LDOX gene up-regulated in the midland population. The maysin portion of the pathway is not included because none of the genes involved were differentially expressed.

Figure S8. Differentially expressed genes between highland and midland maize landraces encoding enzymes involved in lignin biosynthesis


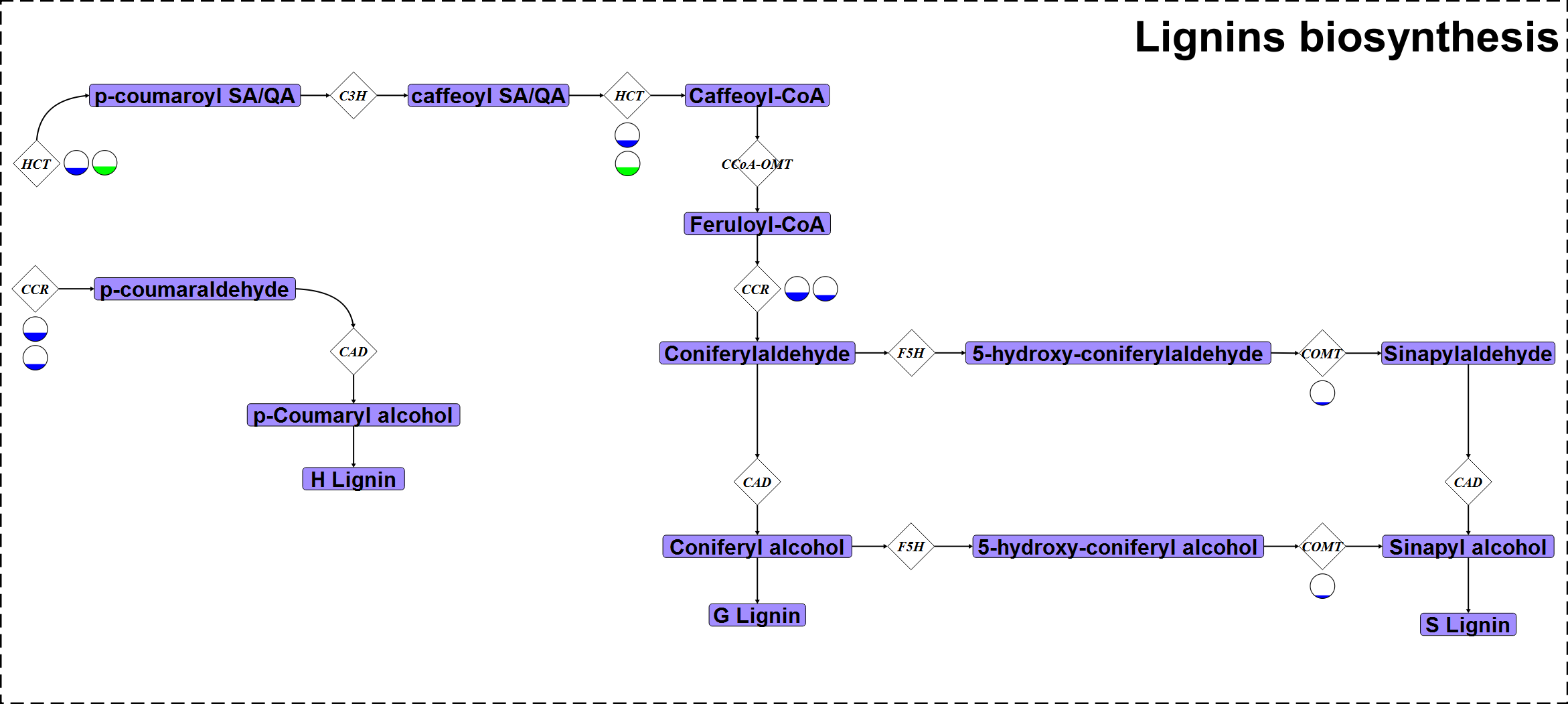


Note. The lignin biosynthesis pathway for maize including metabolic intermediates and enzymes. Blue represents increased expression in highland landraces while green represents increased expression in midland landraces. Full circles represent log2fold changes of 2.11 while half full circles represent log2fold changes of 1.055. Enzymatic steps with multiple circles represent multiple genes encoding enzymes of that type.

Figure S9. Differentially expressed genes between midland and lowland maize landraces encoding enzymes involved in general phenylpropanoid biosynthesis


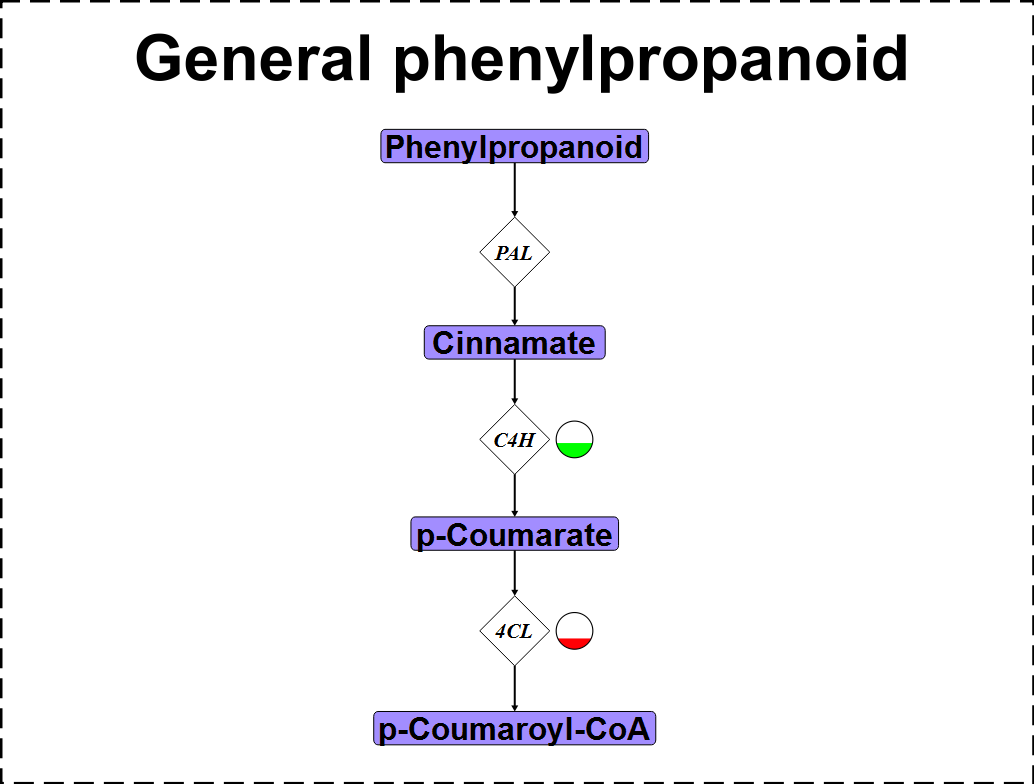


Note. The general phenylpropanoid biosynthesis pathway consisting of three enzymatic steps (white diamonds) and four metabolic intermediates (light purple rectangles). Differentially expressed genes encoding enzymes involved in a biosynthesis pathway are represented to the right or below the enzyme it encodes with a circle filled with either green or red. Green represents increased expression in the highland landraces while red means the gene was highly expressed in the lowland landraces. A completely full circle represents a log2fold change value of 2.11 while a half full circle represents a log2fold change of 1.055. Genes were considered differentially expressed when significant at a 0.05 FDR rate using a Benjamini-Hochberg correction.

Figure S10. Differentially expressed genes between midland and lowland maize landraces encoding enzymes involved in flavonoid biosynthesis


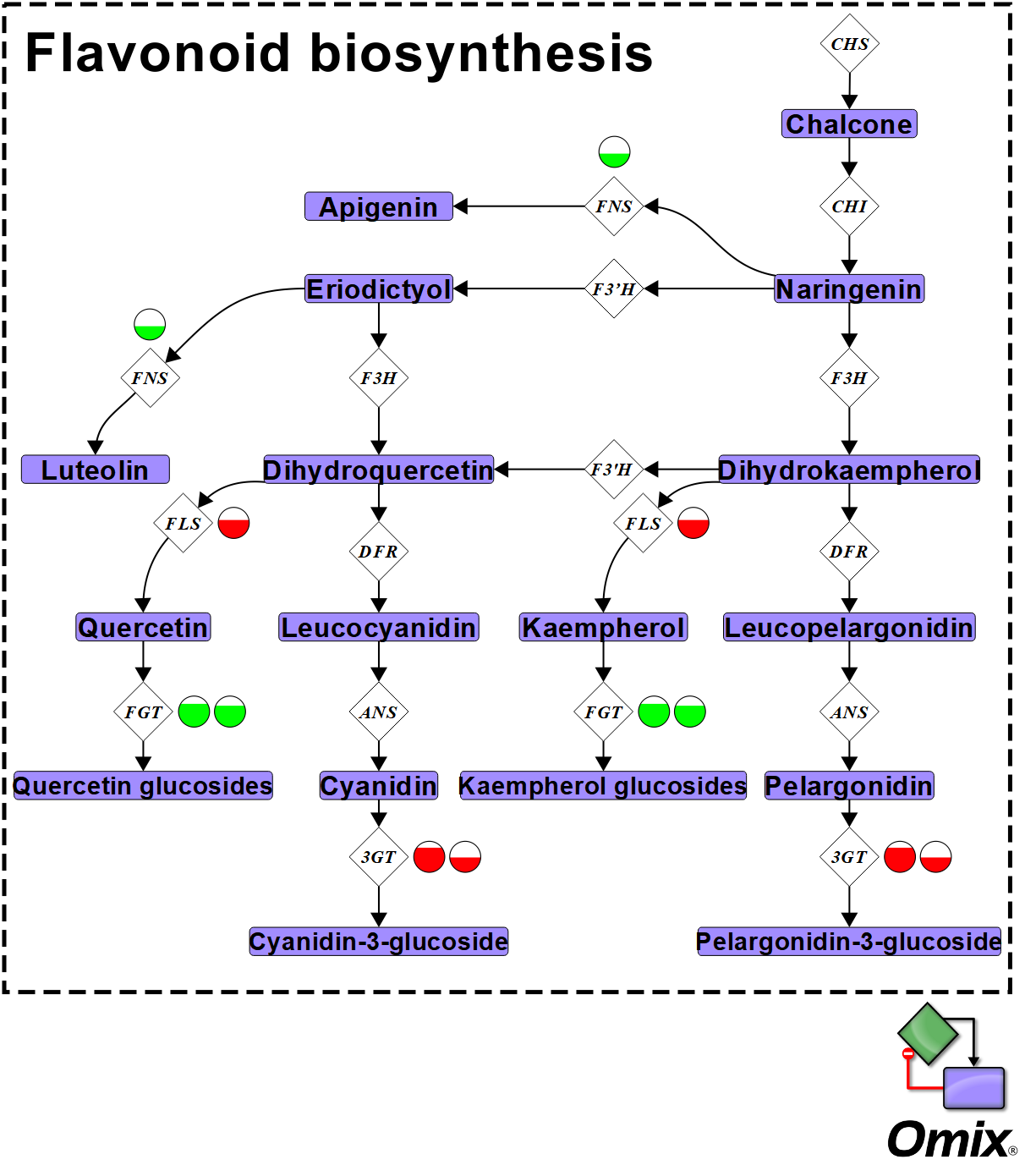


Note. The flavonoid biosynthesis pathway for maize. Genes up-regulated in midland landraces are represented by green and those up-regulated in lowland landraces are represented by red. Full circles represent a log2fold change of 2.11 while half full circles represent log2fold change of 1.055. Multiple circles next to an enzyme represent multiple genes encoding that enzyme. The maysin portion of the pathway is not included because none of the genes involved were differentially expressed. The maysin portion of the pathway is not included because none of the genes involved were differentially expressed.

Figure S11. Differentially expressed genes between midland and lowland maize landraces encoding enzymes involved in lignin biosynthesis


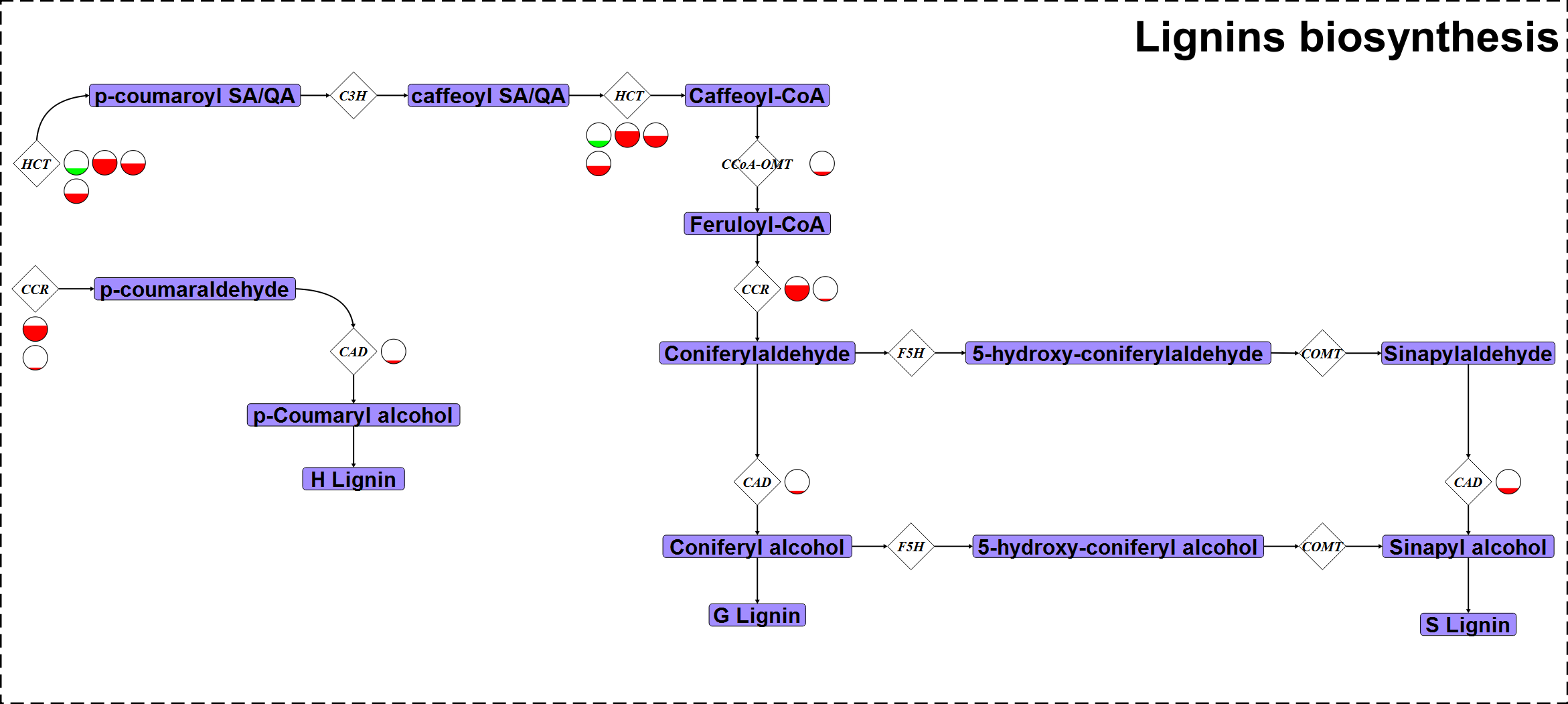


Note. The lignin biosynthesis pathway for maize including metabolic intermediates and enzymes. Full circles represent log2fold changes of 2.11 while half full circles represent log2fold changes of 1.055. Circles containing green represent increased expression in midland populations while red represents increased expression in lowland populations. Enzymatic steps with multiple circles represent multiple genes encoding enzymes of that type.
